# Partial RAG deficiency in humans induces dysregulated peripheral lymphocyte development and humoral tolerance defect with accumulation of T-bet^+^ B cells

**DOI:** 10.1038/s41590-022-01271-6

**Published:** 2022-07-28

**Authors:** Krisztian Csomos, Boglarka Ujhazi, Peter Blazso, Jose L. Herrera, Christopher M. Tipton, Tomoki Kawai, Sumai Gordon, Maryssa Ellison, Kevin Wu, Matthew Stowell, Lauren Haynes, Rachel Cruz, Bence Zakota, Johnny Nguyen, Michelle Altrich, Christoph B. Geier, Svetlana Sharapova, Joseph F. Dasso, Jennifer W. Leiding, Grace Smith, Waleed Al-Herz, Mayra de Barros Dorna, Olajumoke Fadugba, Eva Fronkova, Veronika Kanderova, Michael Svaton, Sarah E. Henrickson, Joseph D. Hernandez, Taco Kuijpers, Snezhina Mihailova Kandilarova, Elizaveta Naumova, Tomas Milota, Anna Sediva, Despina Moshous, Benedicte Neven, Tara Saco, Ravishankar Sargur, Sinisa Savic, John Sleasman, Gauri Sunkersett, Brant R. Ward, Masanobu Komatsu, Stefania Pittaluga, Attila Kumanovics, Manish J. Butte, Michael P. Cancro, Shiv Pillai, Eric Meffre, Luigi D. Notarangelo, Jolan E. Walter

**Affiliations:** 1 Division of Pediatric Allergy/Immunology, University of South Florida at Johns Hopkins All Children’s Hospital, St. Petersburg, FL USA; 2 Department of Pediatrics, University of Szeged, Szeged, Hungary; 3 Cancer and Blood Disorders Institute and Department of Surgery, Johns Hopkins All Children’s Hospital, St. Petersburg, FL USA; 4 Department of Orthopaedic Surgery, Johns Hopkins University School of Medicine, Baltimore, MD USA; 5 Department of Medicine, Division of Rheumatology, Emory University, Atlanta, GA USA; 6 Laboratory of Clinical Immunology and Microbiology, National Institute of Allergy and Infectious Diseases, NIH, Bethesda, MD USA; 7 Department of Pathology & Laboratory Medicine, Johns Hopkins All Children’s Hospital, St Petersburg, FL USA; 8 Eurofins Viracor Laboratories, Lee Summit’s, MO USA; 9 Immunology Outpatient Clinic, Vienna, Austria; 10 Belarusian Research Center for Pediatric Oncology, Minsk, Belarus; 11 Laboratory of Pathology, Center for Cancer Research, National Cancer Institute, National Institutes of Health, Bethesda, MD USA; 12 Department of Pediatrics, Faculty of Medicine, Kuwait University, Kuwait City, Kuwait; 13 Department of Pediatrics, Faculdade de Medicina da Universidade de São Paulo, São Paulo, Brasil; 14 Division of Pulmonary, Allergy and Critical Care, Perelman School of Medicine, University of Pennsylvania, Pennsylvania, PA USA; 15 Childhood Leukemia Investigation Prague, Department of Pediatric Hematology and Oncology, Second Faculty of Medicine, Charles University and University Hospital Motol, Prague, Czech Republic; 16 Allergy Immunology Division, Department of Pediatrics, The Children’s Hospital of Philadelphia, Philadelphia, PA USA; 17 Institute for Immunology, the University of Pennsylvania, Philadelphia, PA USA; 18 Department of Pediatrics, Division of Allergy, Immunology and Rheumatology, Stanford University, Stanford, CA USA; 19 Deptartment of Pediatric Immunology, Rheumatology and Infectious Diseases, Emma Children’s Hospital, Academic Medical Center, Amsterdam, Netherlands; 20 Department of Clinical Immunology, University Hospital Alexandrovska, Medical University, Sofia, Bulgaria; 21 Department of Immunology, Second Faculty of Medicine Charles University and University Hospital Motol, Prague, Czech Republic; 22 Université de Paris, Paris, France; 23 Pediatric Hematology-Immunology and Rheumatology Unit, Necker-Enfants Malades Université Hospital, Assistance Publique-Hôpitaux de Paris, Paris, France; 24 Laboratory of Genome Dynamics in the Immune System, INSERM UMR1163, Institut Imagine, Paris, France; 25 Laboratory of Immunogenetics of Pediatric Autoimmune Diseases, INSERM UMR1163, Institut Imagine, Paris, France; 26 Windom Allergy, Asthma and Sinus, Sarasota, FL USA; 27 Department of Immunology and Allergy, Sheffield Teaching Hospitals, Sheffield, UK; 28 Department of Clinical Immunology and Allergy, St James’s University Hospital, Leeds, UK; 29 National Institute for Health Research–Leeds Musculoskeletal Biomedical Research Centre and Leeds Institute of Rheumatic and Musculoskeletal Medicine, St James’s University Hospital, Leeds, UK; 30 Division of Allergy, Immunology and Pulmonary Medicine, Duke University School of Medicine, Durham, NC USA; 31 Cancer and Blood Disorder Institute, Johns Hopkins All Children’s Hospital, St. Petersburg, FL USA; 32 Division of Allergy and Immunology, Children’s Hospital of Richmond, Virginia Commonwealth University, Richmond, VA USA; 33 Department of Laboratory Medicine and Pathology, Mayo Clinic, Rochester, MN USA; 34 Division of Immunology, Allergy, and Rheumatology, Department of Pediatrics and Jeffrey Modell Diagnostic and Research Center, University of California, Los Angeles, Los Angeles, CA USA; 35 Department of Pathology, Perelman School of Medicine, University of Pennsylvania, Pennsylvania, PA USA; 36 Ragon Institute of Massachusetts General Hospital, Massachusetts Institute of technology and Harvard University, Cambridge, MA USA; 37 Department of Immunobiology, Yale University, New Haven, CT USA; 38 Section of Rheumatology, Allergy and Clinical Immunology, Yale School of Medicine, New Haven, CT USA; 39 Division of Allergy and Immunology, Massachusetts General Hospital for Children, Boston, MA USA

**Keywords:** Peripheral tolerance, Primary immunodeficiency disorders

## Abstract

The recombination-activating genes (*RAG*) 1 and 2 are indispensable for diversifying the primary B cell receptor repertoire and pruning self-reactive clones via receptor editing in the bone marrow; however, the impact of *RAG1*/*RAG2* on peripheral tolerance is unknown. Partial RAG deficiency (pRD) manifesting with late-onset immune dysregulation represents an ‘experiment of nature’ to explore this conundrum. By studying B cell development and subset-specific repertoires in pRD, we demonstrate that reduced RAG activity impinges on peripheral tolerance through the generation of a restricted primary B cell repertoire, persistent antigenic stimulation and an inflammatory milieu with elevated B cell-activating factor. This unique environment gradually provokes profound B cell dysregulation with widespread activation, remarkable extrafollicular maturation and persistence, expansion and somatic diversification of self-reactive clones. Through the model of pRD, we reveal a *RAG*-dependent ‘domino effect’ that impacts stringency of tolerance and B cell fate in the periphery.

## Abstract (web summary)

Patients with partial recombination-activating gene (*RAG*) deficiency (pRD) present variable late-onset autoimmune clinical phenotypes. Walter and colleagues identified a restricted primary B cell antigen receptor repertoire enriched for autoreactivity and clonal persistence in pRD. They described dysregulated B cell maturation with expansion of T-bet^+^ B cells revealing how *RAG* impacts stringency of tolerance and B cell fate in the periphery.

## Main

RAG 1 and 2 orchestrate the process of V(D)J recombination during the early stage of B cell development in the bone marrow (BM)^1^. In humans, pathogenic biallelic hypomorphic *RAG* variants decrease but do not fully abrogate the recombinase activity of the RAG proteins^2,3^ and result in restricted T cell antigen receptor (TCR)/B cell antigen receptor (BCR) repertoires and low-to-normal peripheral blood lymphocytes with variable autoantibody profiles^4,5^.

As the diverse pre-immune BCR repertoire forms, self-reactive, potentially harmful clones are also naturally generated^6^. To maintain self-tolerance, three central tolerance mechanisms (receptor editing, deletion and anergy) efficiently purge the majority of nascent self-reactive B cell clones^6,7^. As the *RAG1*/*RAG2* complex plays a direct role in receptor editing^8^, pRD could lower its efficiency allowing the inclusion of self-reactive clones in the peripheral B cell repertoire. Nevertheless, in normal circumstances, functional peripheral tolerance compensates for impaired central tolerance by suppressing autoreactive clones in the pre-immune B cell pool to prevent humoral autoimmunity^9^.

It is unclear whether and how defects in *RAG1*/*RAG2* impact peripheral B cell tolerance. Expansion of autoreactive B cells and spontaneous autoantibody production occur in mouse models of pRD, implying broken tolerance^10,11^. Patients manifesting with late-onset phenotype of pRD with immune dysregulation, termed ‘combined immune deficiency with granuloma/autoimmunity’ (CID-G/AI)^12^ are present with multiorgan autoimmune disease with high titers of serum autoantibodies, including those targeting cytokines^5^. The remnant recombinase activity only partially correlates with the clinical phenotype^13,14^ and the same *RAG* genetic variant, even within the same family, can result in a spectrum from asymptomatic to variable autoimmunity^15,16^, which may worsen with age and exposure to environmental antigens^3,5,17–22^. Accordingly, chronic Toll-like receptor (TLR) stimulation mimicking viral infection in a mouse model resulted in a broadening autoantibody profile^5^.

Collectively, these indicate that, although *RAG1* and *RAG2* are expressed in developing B lymphocytes in the BM, they may impact B cell development and tolerance filters indirectly in the periphery. Here, in a cohort of patients with pRD we describe impaired primary BCR repertoire formation with remarkable alterations in the composition of B cell subsets, along with widespread, promiscuous activation that favors extrafollicular destiny and expansion of poly/autoreactive B cell clones in the periphery. Our results shed light on the mechanisms underlying complex immune dysregulation induced by pRD that affects multiple tolerance checkpoints and B cell fate in the periphery.

## Results

### Genetic and clinical features of patients with pRD

**Extended Data Fig. 1:**
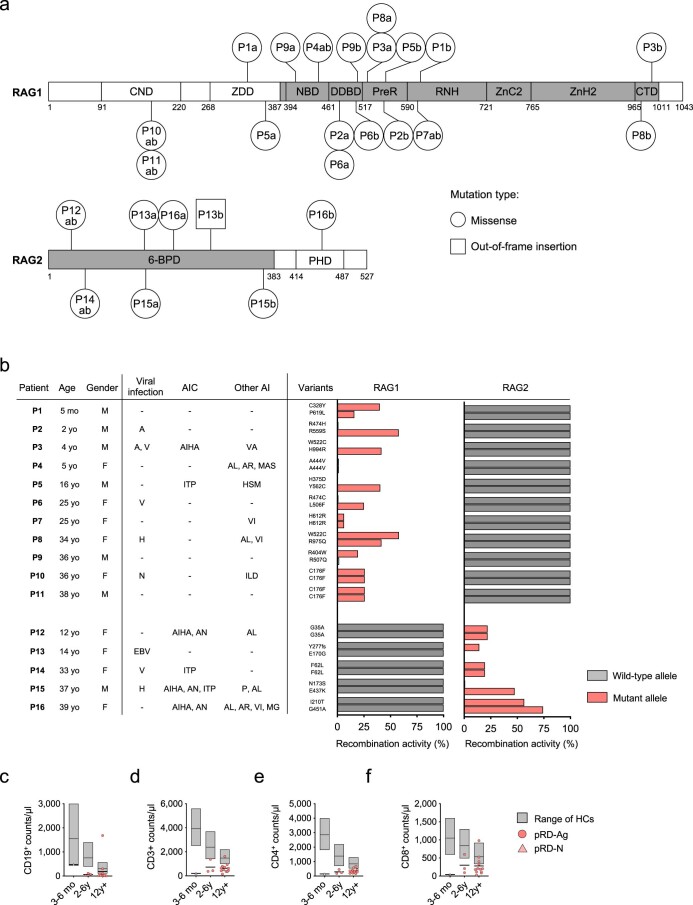
*RAG1/2* mutations, recombinase activity and clinical features. **(a) Distribution of mutations in the** ***RAG1/2*** **genes**. Mutations are indicated for each patient as patient ID and mutant alleles, with *a* and *b* separately for compound heterozygous or *ab* for homozygous variants. Core and non-core regions of the RAG proteins are denoted by gray and white, respectively. RAG1 domains: central non-core (CND), zinc dimerization (ZDD), nonamer-binding (NBD), dimerization and DNA-binding (DDBD), pre-RNase H (PreR), catalytic RNase H (RNH), zinc-binding (with cysteine and histidine zinc-binding residues (ZnC2 and ZnH2)) and carboxy-terminal (CTD). The RAG2 core region is a six-bladed propeller domain (6-BPD). Non-core RAG2 contains a plant homology domain (PHD). Numbers indicate amino acids at domain borders. **(b) Residual RAG recombinase activities by variants**. RAG recombinase activities are shown as percentage of wild-type variants for both alleles (see Supplementary Table 2 for details). Patient information with clinical presentations is shown (M: male, F: female, A: adenovirus, V: varicella, H: herpesvirus, EBV: Epstein-Bar virus, AIHA: autoimmune hemolytic anemia, ITP: immune/idiopathic thrombocytopenic purpura, AN: autoimmune neutropenia, VA: vasculitis, AL: alopecia, AR: arthritis, MAS: macrophage activation syndrome, HSM: hepatosplenomegaly, VI: vitiligo, ILD: interstitial lung disease, P: psoriasis, MG: myasthenia gravis. **(c-f) Lymphocyte counts in pRD patients**. Graphs show CD19^+^, CD3^+^, CD4^+^, CD8^+^ lymphocyte counts in the peripheral blood of pRD-N (n = 1) and pRD-Ag patients (n = 16) in age groups 3-6 month, 2-6 years and over 12 years, compared to published aged-matched healthy ranges. Values for HCs are presented as floating bars with medians and 10^th^ percentiles and 90^th^ percentiles shown obtained as 3-6 month (n = 81), 2-6 years (n = 90) and over 12 years (n = 90), from published data. Values for patients are depicted individually. Source data

The pRD cohort included a 5-month-old asymptomatic male (P1)^21^ and 15 patients with a CID or CID-G/AI phenotype (P2–16) (Supplementary Table 1). Eleven patients carried *RAG1* (7 compound heterozygous and 4 homozygous) and 5 patients carried *RAG2* (3 compound heterozygous and 2 homozygous) variants, for a total of 16 *RAG1* and 8 *RAG2* distinct mutant alleles (Extended Data Fig. 1a). Pathogenicity was assigned based on combined assessment of in vitro recombination activity assays^2,3^ and curated data obtained from ClinVar database and/or analysis following guidelines of American College of Medical Genetics and Genomics^23^ and the Association for Molecular Pathology (Extended Data Fig. 1b and Supplementary Table 2).

All patients displayed profound lymphopenia as compared to aged-matched healthy ranges^24^ (Extended Data Fig. 1c–f), except P13 who had chronic Epstein–Barr virus (EBV) and Cytomegalovirus (CMV) viremia with lymphoproliferation^25^. Asymptomatic P1 was considered to be antigen-naive (pRD-N), whereas symptomatic P2–16 were grouped as antigen-experienced patients (pRD-Ag). P1 remained CMV negative and showed no clinical or laboratory signs of infections until successful hematopoietic stem cell transplantation^21^. All patients with pRD-Ag had upper and/or lower respiratory tract infections, including bronchiectasis in eight cases. Seven patients had one or a combination of adenovirus or herpesvirus infections (EBV, CMV, HSV and varicella). Ten patients had autoimmunity and three patients had granulomas affecting lung, skin and/or spleen (Extended Data Fig. 1b).

### Diverse autoantibody profiles in patients with pRD

**Extended Data Fig. 2:**
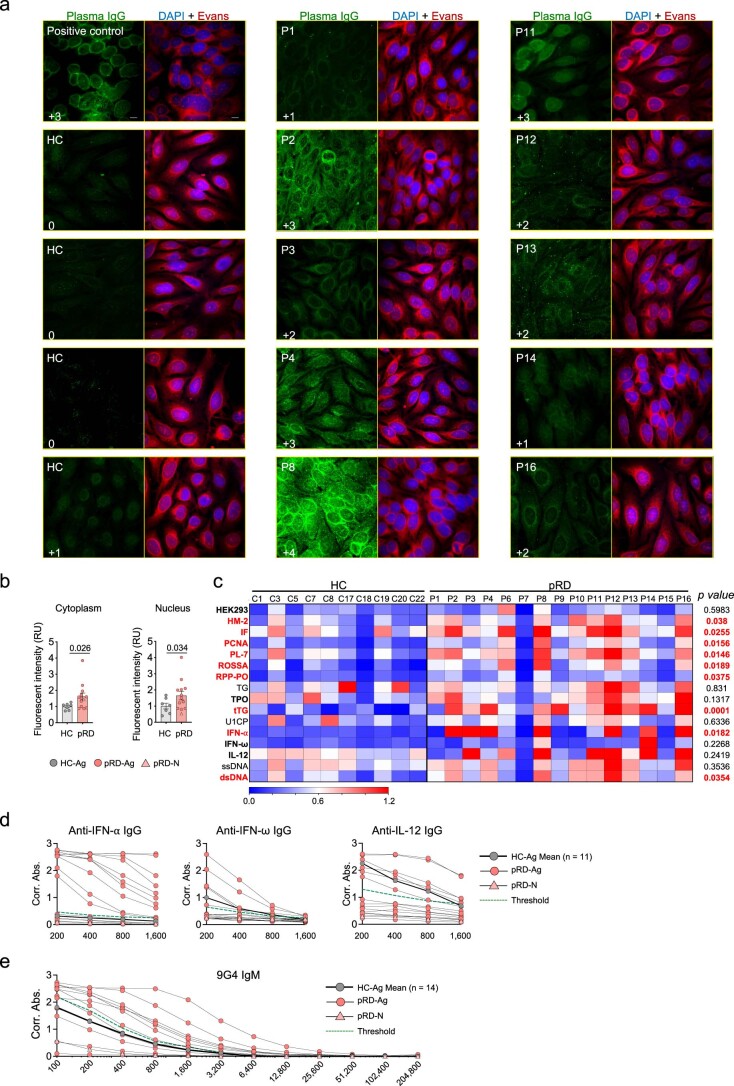
Autoantibody detection from plasma samples. **(a) Immunofluorescent analysis (IFA) of plasma samples on Hep2 slides using confocal microscopy**. Representative confocal microscopy images of Hep2 slides stained with HC-Ags (n = 7), pRD-N (n = 1) and pRD-Ags patients (n = 12) plasma samples and detected with anti-IgG (green). Staining for all samples were performed simultaneously in a single experiment with two technical replicates for each. Score is depicted in left bottom corner for each sample. Nucleus (DAPI, blue), cytoplasm (Evans Blue, red). Magnification: 60x. **(b) Quantification of cytoplasmic and nuclear Hep2 positivity**. Bars represent mean±s.e.m. with individual values from HC-Ags (n = 7), pRD-N (n = 1) and pRD-Ags patients (n = 12). Two-sided unpaired Student’s *t*-test. **(c) Heatmap of autoantigen reactivities**. Binding of plasma IgGs to a set of autoantigens (human embryonic kidney 293 cell protein extract, human M2 antigen, intrinsic factor, proliferating cell nuclear antigen, tissue transglutaminase, threonyl-tRNA Synthetase, Ro/SSA (52 kDa), thyroid peroxidase, U1-SnRNP C protein, thyroglobulin, ribosomal phosphoprotein P0, IFN-α, IFN-ω, IL-12, single and double stranded DNA were measured with ELISA in 400x dilution. Blank corrected absorbance values are shown by HC-Ags (n = 10) pRD-N (n = 1) and pRD-Ag (n = 14) subjects. Antigens, with z score above 2 in three or more patients were considered as common autoantigens and are labeled with bold (left). Statistical analysis was performed using two-sided unpaired Student’s *t*-test on Ag-experienced subjects (excluding pRD-N, P1). *P* values for differences between HC-Ags and pRD-Ag patients are shown (right) and the statistically significant differences are depicted by red. **(d) Anti-cytokine auto-Ab levels**. Binding of plasma IgGs to IFN-α, IFN-ω, IL-12 was measured with ELISA in 2-fold serial dilution (200–1,600-fold). **(e) IgM 9G4 auto-Ab levels**. Plasma 9G4 auto-Abs were measured with ELISA in 2x serial dilution (100 – 204,800-fold). Cut-off for positivity on **b** and **c** is indicated with green dashed line defined as z = 2 at each dilution. Mean values of HC-Ags for **c** and **d** (n = 11 or 4, respectively) are depicted with thick black line and dark gray circles, individual values for pRD-N (n = 1) and pRD-Ag (n = 14) patients are shown. ELISAs for **c-e** were performed with two technical replicates. Source data

We detected autoantibodies in 11 plasma samples of 13 patients tested by immunofluorescent analysis (IFA) on HEp-2 cell slides with various intensities (very bright, bright, intermediate and low positive; 1, 4, 4 and 2 patients, respectively), whereas healthy controls (HCs) showed no or low positive staining (Extended Data Fig. 2a). Staining patterns included homogeneous nuclear (P11 and P14), speckled cytoplasmic (P1, P2, P4, P8, P9, P12 and P13) and cytoplasmic reticular/mitochondrial (P3 and P16). Quantitative image analysis confirmed stronger cytoplasmic and nuclear staining in patients compared to HCs (Extended Data Fig. 2b,c, respectively). Eleven patients from 15 produced IgG autoantibodies against at least one specific antigen as tested by ELISA (defined as *z* score >2) and autoantibody levels against nine antigens were significantly higher in patients compared to HCs (Extended Data Fig. 2d). IgG autoantibodies to interferon (IFN)-α, IFN-ω and/or interleukin (IL)-12, a hallmark of pRD^5,26^, were present in ten patients with pRD-Ag (newly detected in four patients and previously published in six cases^5^) but not in pRD-N (Extended Data Fig. 2e). We also detected VH4-34-encoded IgM 9G4 antibodies that recognize polysaccharide antigens in red blood cells^27^ in eight patients (Extended Data Fig. 2f); hence, the presence of a wide range of IgM and IgG autoantibodies indicates a failed B cell tolerance in pRD.

### Dysregulated peripheral B cell maturation and activation

**Fig. 1:**
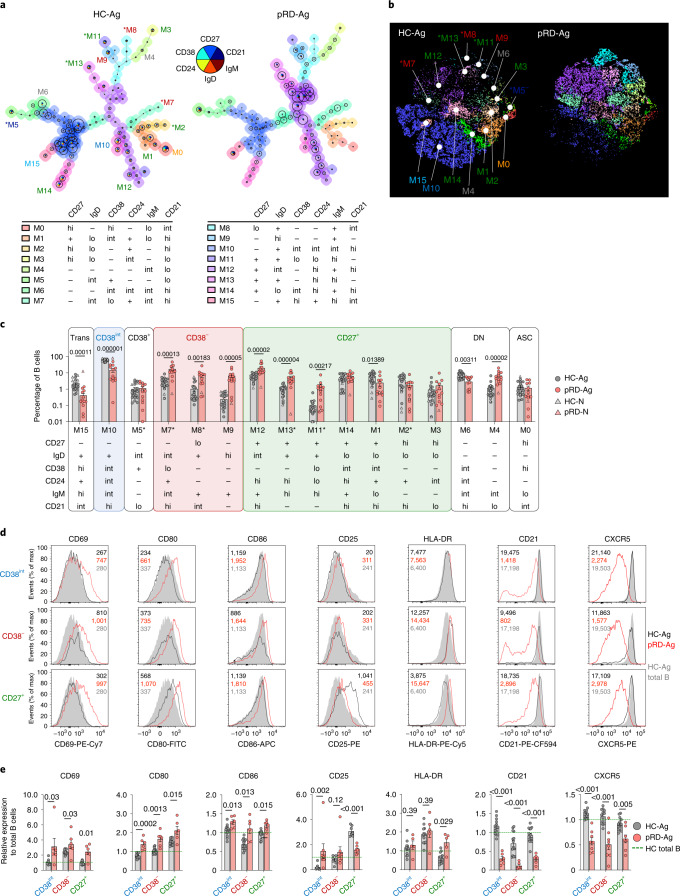
Immunophenotyping of peripheral blood B cells. **a**, Automated B cell subset identification by FlowSOM. Minimal spanning trees of a representative HC and a patient with pRD are shown. Metaclusters, corresponding to individual B cell subsets are indicated by the background color of the nodes, numbers (M0–15) and marker expression pattern. **b**, B cell subset composition. Metaclusters from concatenated live B cells of four HCs and four patients with pRD were projected onto *t*-SNE space and assigned as in **a**. **c**, Frequencies of the specific B cell subsets. Circles represent antigen-experienced donors, HCs (HC-Ag, *n* = 18) and patients with pRD (pRD-Ag, *n* = 13); triangles represent antigen-naive infant donors (HC-N, *n* = 4; pRD-N *n* = 1). Subsets comprising Trans, CD38^int^, CD38^+^, CD38^−^, CD27^+^, DN and ASC populations are depicted with different backgrounds. Statistical analyses were performed on individuals with antigens (HC-Ag versus pRD-Ag) (Mann–Whitney *U*-test with Holm–Šídák multiple comparison). Asterisks indicate new B cell subsets for **a**–**c**. **d**, Activation of peripheral blood B cells. Surface expression of CD69, CD25, HLA-DR, CD80, CD86, CD21 and CXCR5 in CD38^int^, CD38^**−**^ and CD27^+^ B cells is shown as histograms. Filled gray histograms depict the expression of each marker on the total B cells from an HC. Dark gray and red lines represent the expressions of each marker in the indicated compartments (CD38^int^, CD38^**−**^ or CD27^+^) from an HC and a patient with pRD, respectively. Geometric mean fluorescence intensities (gMFIs) are indicated for each HC (black) and pRD B cell subsets (red) and HC total B cells (gray). **e**, Expression of activation markers. gMFI of each marker from CD38^int^, CD38^**−**^ and CD27^+^ compartments were normalized by donors to the gMFIs of the total B cells from the HC(s) used in each experiment. Data are shown as mean ± s.e.m. with individual values. Therefore, changes in the expression by compartments in HC-Ag (gray, *n* = 14) and patients with pRD-Ag (red, *n* = 6–8) are shown as relative to 1 (green dashed line), which represents marker expression level in the total B cells of HCs. Data were analyzed by Mann–Whitney *U*-test. Source data

**Extended Data Fig. 3:**
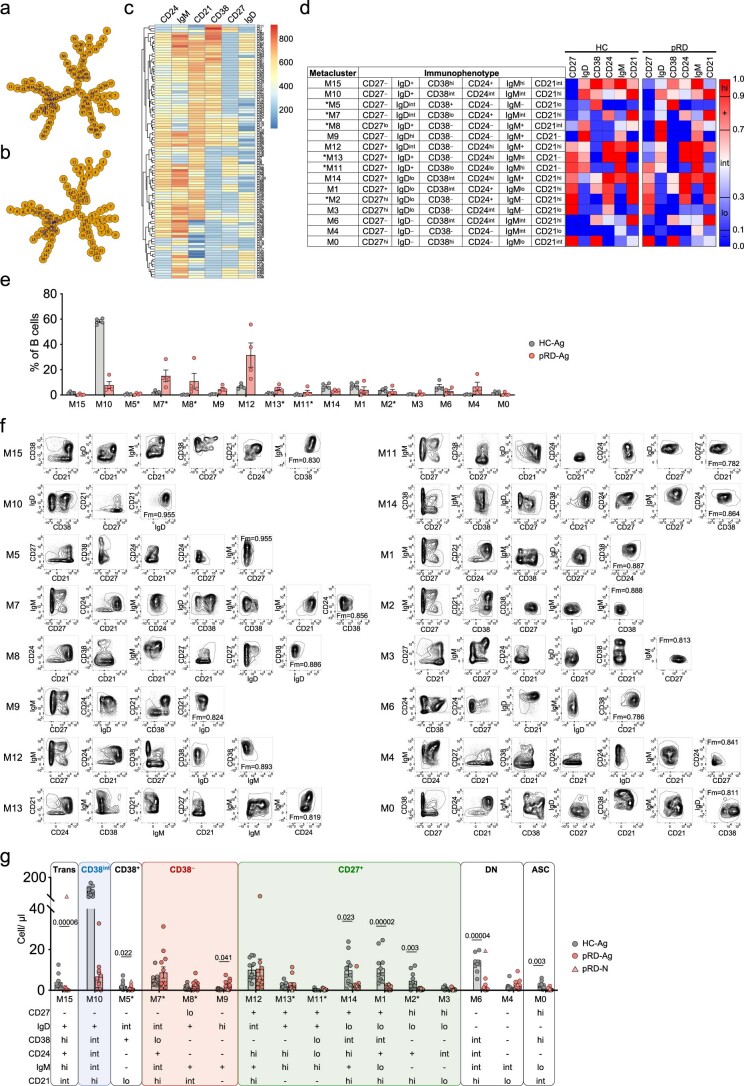
Automated unsupervised B cell subset identification by FlowSOM. **(a). Minimal spanning tree (MSP)**. MSPs were built as described in Methods to visualize similar nodes in branches and their relative connection to each other. Nodes are numbered as 1–100. **(b). Metaclustering**. Based on similarities, nodes were classified into 16 metaclusters (M0–15), each representing a unique B cell subset. **(c). Relative expression of surface markers**. Heat map represents the relative expression level of CD21, CD24, CD27, CD38, IgD and IgM on the B cells concatenated from four HC-Ags and four pRD-Ag patients by nodes. **(d) B cell subset assignment**. Metaclusters were assigned to B cell subsets as described in Methods. Novel subsets are depicted with asterisk. Subsets are organized according to possible developmental stages. Immunophenotypic definition of each subset is indicated. Heat map represents the normalized mean expression values of each surface markers for the HC-Ag (n = 4) and pRD-Ag patients (n = 4) separately corresponding to each metaclusters. Scale shows normalized value ranges for the expression of each marker defined as negative (−), low (lo), intermediate (int), positive (+) and high (hi). **(e) Subset frequencies by FlowSOM**. Frequencies of each metacluster from HC-Ags (n = 4) and pRD-Ag patients (n = 4) are shown as mean±s.e.m. with individual values. **(f) Subset identification by HyperFinder**. Two-dimensional sequential gating was constructed by HyperFinder FlowJo plugin using dual combinations of CD21, CD24, CD27, CD38, IgD and IgM parameters. Contour plots were generated on the data set concatenated from the four pRD-Ag patients and four age-appropriate HC-Ag. Fm-scores are inserted in the last plot of the sequential gating for each B cell subset. **(g) Cell number of the specific B cell subsets**. Data are shown as mean±s.e.m. with individual values from HC-Ag (n = 11), pRD-Ag (n = 11) and pRD-N (n = 1). Mann–Whitney *U* test with multiple comparison (Holm-Šídák method), P13 with lymphoproliferation was excluded from the analysis. Source data

To assess peripheral B cell subsets, we performed unsupervised high-dimensional analysis from the peripheral blood of patients with pRD (Extended Data Fig. 3a–f). We identified 16 distinct metaclusters (M0–15), each corresponding to a unique B cell population with a specific surface marker expression pattern (Fig. 1a). Visualization of the B cell compartment composition by the 16 metaclusters with *t*-distributed stochastic neighbor embedding (*t*-SNE) indicated remarkable reduction in M15 and M10, whereas the M7–9 and M11–13 populations were expanded in pRD when compared to HCs (Fig. 1b).

Quantitative analysis confirmed the profound reduction of M15 (CD27^−^IgD^+^CD38^hi^CD24^+^IgM^hi^CD21^int^ corresponding to transitional B cells) and M10 (CD27^−^IgD^+^CD38^int^CD24^int^IgM^hi^CD21^hi^, corresponding to mature resting naive B cells in healthy individuals^28^, referred to as ‘CD38^int^’) in pRD when compared to HCs (Fig. 1c).

Notably, three CD27^−^IgD^+^CD38^lo/−^ metaclusters, M7–9, were expanded in patients. Specifically, M7 and M8 were identified as new specific B cell subsets (CD27^−^IgD^int^CD38^lo^CD24^+^IgM^int^CD21^hi^ and CD27^lo^IgD^+^CD38^−^CD24^−^IgM^+^CD21^int^, respectively) as these populations have not been described in humans, neither in healthy nor pathological conditions (Fig. 1c). The third CD27^−^IgD^+^CD38^lo/−^ population (M9) was phenotypically identical to the recently described ‘activated naive’ B cells (CD27^−^IgD^hi^CD38^−^CD24^−^IgM^+^CD21^lo^)^28,29^. As M7–9 became the predominant fraction of the CD27^−^IgD^+^ compartment in pRD, we collectively referred to them as CD38^−^ in our further investigations.

Among CD27-expressing metaclusters (conventional memory compartment and collectively referred as CD27^+^) we identified M11–14 that expressed IgM, therefore representing non-switched memory (NSM) B cells. M12 corresponded to NSM resting cells (CD27^+^IgD^int^CD38^−^CD24^hi^IgM^+^CD21^hi^) and was expanded in pRD, whereas M14 represented ‘pre-switched memory B cells’ (CD27^+^IgD^lo^CD38^int^CD24^hi^IgM^+^CD21^hi^)^28^. In addition, we defined M11 and M13 as new NSM cells (CD27^+^IgD^+^CD38^−^CD24^hi^IgM^+^CD21^−^ and CD27^+^IgD^+^CD38^lo^CD24^lo^IgM^+^CD21^−^, respectively) that were significantly expanded in pRD (Fig. 1c). CD27^+^IgD^lo/−^IgM^lo/−^ switched memory (SM) fraction harbored the resting SM B cells (M1, CD27^+^IgD^lo^CD38^int^CD24^+^IgM^lo^CD21^hi^)^28^, which were significantly decreased in pRD and M2–3 (CD27^+^IgD^lo^CD38^−^CD24^+^IgM^−^CD21^hi^ and CD27^+^IgD^lo^CD38^−^CD24^int^IgM^−^CD21^lo^, respectively) (Fig. 1c). These alterations in the CD27^+^ populations resulted in the dominance of NSM over SM B cells in pRD when compared to HCs.

We identified M6 (CD27^−^IgD^−^CD38^int^CD24^int^IgM^int^CD21^hi^) and M4 (CD27^−^IgD^−^CD38^−^CD24^−^IgM^int^CD21^lo^), as CD27^−^IgD^−^ double negative (DN) subsets, corresponding to DN1 and DN2, respectively^28^. Notably, DN1:DN2 (M6:M4) ratio in pRD was skewed toward DN2 (M4) similarly to what is seen in systemic lupus erythematosus^30^ (Fig. 1c). Finally, a decrease in pRD M0 (antibody-secreting cells (ASCs), CD27^hi^IgD^−^CD38^hi^CD24^−^IgM^lo^CD21^int^) was also noticed, although the difference was not statistically significant.

**Extended Data Fig. 4:**
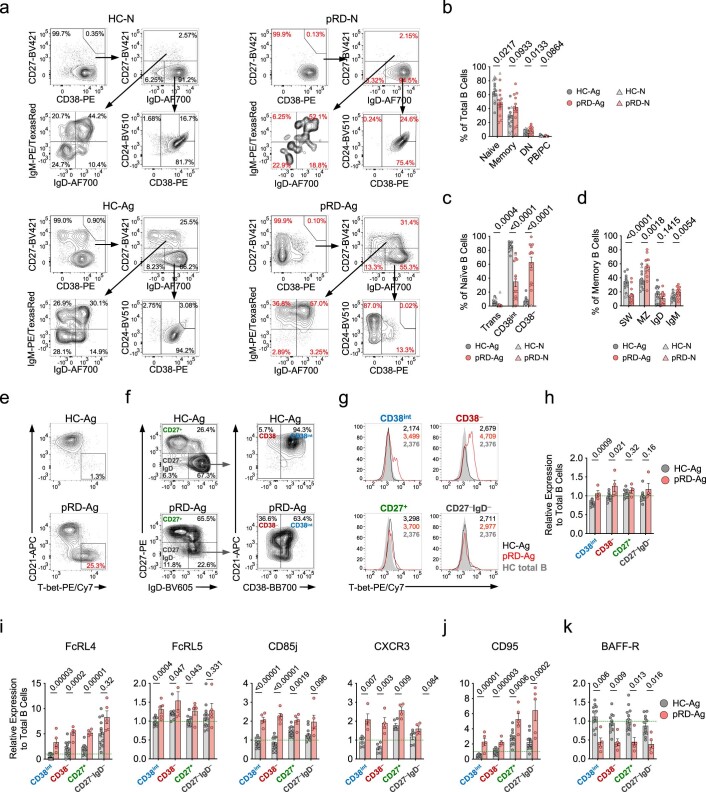
Extended B cell phenotyping. **(a) Fractioning B cells by conventional standard gating**. Plots show a representative HC-Ag, HC-N, pRD-Ag and the pRD-N subjects. **(b) Major B cell subset frequencies**. Frequencies of naive (CD27^−^IgD^+^), memory (CD27^+^), DN (CD27^−^IgD^−^) and plasmablast/plasmacell (CD27^hi^CD38^hi^) B cell populations are shown. **(c) Transitional, CD38**^**int**^ **and CD38**^**−**^ **subsets frequencies from CD27**^**−**^**IgD**^**+**^ **gate. (d) Memory B cell subsets frequencies from CD27**^**+**^ **gate**. For h-j statistical analyses were performed on Ag subjects (HC-Ag vs pRD-Ag) using Mann–Whitney *U* test with multiple comparison (Holm-Šídák method). Bars represent mean±s.e.m. with individual values from HC-Ag (n = 13), pRD-Ag (n = 13), HC-N (n = 3) and pRD-N (n = 1). **(e) Detection of T-bet**^**+**^ **B cells**. Gates show the CD21^low^T-bet^+^ B cells in a representative from HC-Ag and a pRD-Ag patient. Frequencies are indicted. **(f) Gating for CD38**^**int**^**, CD38**^**−**^**, CD27**^**+**^ **and DN (CD27**^**−**^**IgD**^**−**^**) B cells**. Plot show gatings from a representative HC-Ag and a pRD-Ag patient. **(g) T-bet expression in CD38**^**int**^**, CD38**^**−**^**, CD27**^**+**^ **and DN (CD27**^**−**^**IgD**^**−**^**) B cells**. Overlaid histogram plots depict the T-bet expression by CD38^int^, CD38^−^, CD27^+^ and DN (CD27^−^IgD^−^) B cells in a representative HC-Ag subject (black) and a pRD-Ag patient (red) in comparison to HC total B cell (filled gray). Expression levels are indicated as gMFI values. **(h) T-bet expression in CD38**^**int**^**, CD38**^**−**^**, CD27**^**+**^ **and CD27**^**−**^**IgD**^**−**^ **populations**. **Expression of FcRL4, FcRL5, CD85j and CXCR3 ABC-specific markers (i), CD95 (j) and BAFF-R (k) in CD38**^**int**^**, CD38**^**−**^**, CD27**^**+**^ **and CD27**^**−**^**IgD**^**−**^ **B cells**. Plots for **e-g** were obtained from a single experiment performed simultaneously on HC-Ags (n = 10) and pRD-Ag patients (n = 4). Data for **h** was obtained from a single experiment performed simultaneously on HC-Ags (n = 10) and pRD-Ag patients (n = 4). Data for **i-k** (except for CXCR3) was obtained from a single experiment performed simultaneously on HC-Ags (n = 13) and pRD-Ag patients (n = 5). Data for **i** CXCR3 panel was obtained from a single experiment performed simultaneously on HC-Ags (n = 6) and pRD-Ag patients (n = 4). Data for each graph are normalized and shown as in Fig. 1e and shown as mean±s.e.m. with individual values depicted. Statistical analyses were performed using two-sided unpaired Student’s *t*-test. Source data

In absolute counts, most B cell subsets were decreased in pRD compared to HCs, reflecting their B cell lymphopenia; however, M9 ‘activated naive’ B cells were significantly elevated (Extended Data Fig. 3g). Analysis of B cell subsets with conventional standard gating (Extended Data Fig. 4a) confirmed that transitional, mature resting naive and SM B cells, were reduced, whereas atypical CD38^−^ naive B cells and marginal zone-like B cells were greatly expanded in pRD (Extended Data Fig. 4b–d).

In addition, all three B cell subsets (CD38^int^, CD38^−^ and CD27^+^) defined above displayed increased activation status in pRD as assessed by the expression of CD69, CD80 and CD86 (Fig. 1d,e). We also found significantly elevated levels of CD25 and HLA-DR in the CD38^int^ and the CD27^+^ compartments of the patients, respectively (Fig. 1d,e). Decrease in the expression of CD21, indicating previous history of B cell activation^31^, was detected in the patients, offering further evidence of promiscuous B cell activation in pRD (Fig. 1d,e). Finally, we detected significantly lower expression of the follicle homing receptor, CXCR5, on each B cell subset of patients with pRD compared to HCs (Fig. 1d,e).

In summary, the B cell compartment in patients with pRD-Ag displayed remarkable activation and subset dysregulation with expansion of non-conventional B cells. Notably, asymptomatic P1 (pRD-N) did not show these changes, thus, dysregulated B cell maturation with promiscuous activation in pRD is likely a dynamic process that worsens with age and disease state, implying the role of environmental triggers.

### Expansion of non-conventional T-bet^+^ B cells

**Fig. 2:**
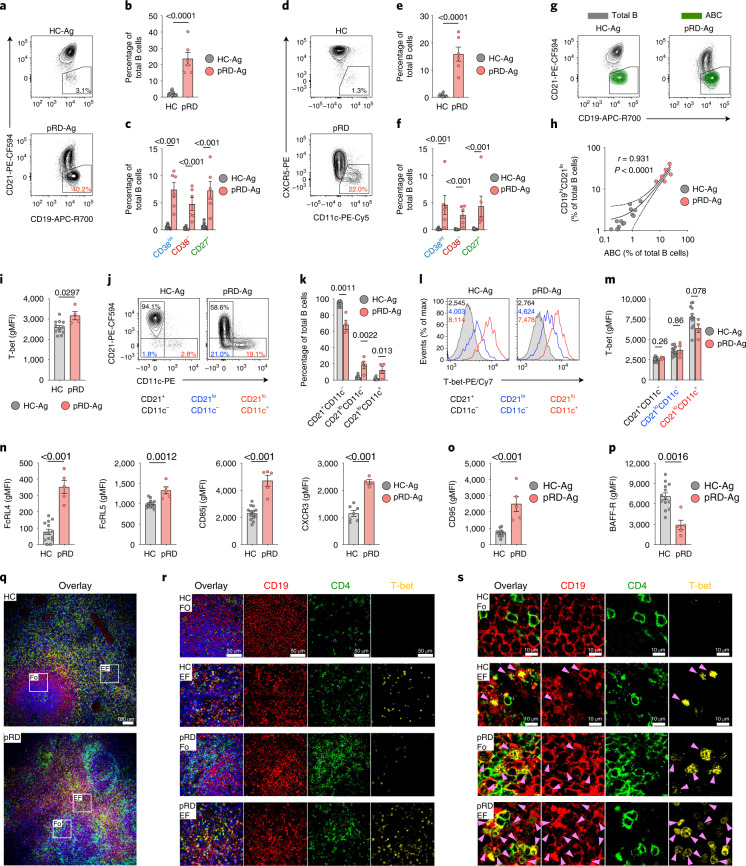
T-bet^+^ B cells. **a**, Detection of CD19^hi^CD21^lo^ B cells. **b**,**c**, Gating for CD19^hi^CD21^lo^ B cells in a representative HC-Ag and patient with pRD-Ag. Fraction of CD19^hi^CD21^lo^ B cells in total (**b**) and CD38^int^, CD38^**−**^ and CD27^+^ B cells (**c**) in HC-Ags (*n* = 14) and patients with pRD-Ag (*n* = 6). **d**, Detection of ABCs. Gating for ABCs in a representative HC-Ag and patient with pRD-Ag. **e**,**f**, Fraction of ABCs in total (**e**) and CD38^int^, CD38^**−**^ and CD27^+^ B cells (**f**) in HC-Ags (*n* = 14) and patients with pRD-Ag (*n* = 6). **g**, Distribution of ABCs in conventional and CD19^hi^CD21^lo^ B cells. Contour plots show total B cells (gray), gate depicts CD19^high^CD21^low^ B cells and ABCs are overlayed (green) in a representative HC-Ag and patient with pRD-Ag. **h**, Correlation between CD19^hi^CD21^lo^ B cells and ABCs. Linear regression line is shown with 95% confidence intervals, Pearson *r* and *P* values are shown. Data were obtained from HC-Ags (gray, *n* = 14) and patients with pRD-Ag (red, *n* = 6). **i**, T-bet expressions in total B cells are shown as gMFIs. **j**,**k**, Detection (**j**) and fraction (**k**) of CD21^+^CD11c^−^, CD21^lo^CD11c^−^ and CD21^lo^CD11c^+^ B cell populations. Gating is shown in a representative HC-Ag and patient with pRD-Ag. **l**,**m**, T-bet expression in CD21^+^CD11c^−^, CD21^lo^CD11c^−^ and CD21^lo^CD11c^+^ B cells in a representative HC-Ag and patient with pRD-Ag (**l**) and shown as gMFIs (**m**). **n**–**p**, Expressions of FcRL4, FcRL5, CD85j and CXCR3 (**n**), CD95 (**o**) and BAFF-R (**p**) in total B cells are shown as gMFIs. Data for **i**–**p** were obtained from HC-Ags (*n* = 7–14) and patients with pRD-Ag (*n* = 4–5). Data for **b**,**c**,**e**,**f**,**k**–**o** are shown as mean ± s.e.m. with individual values depicted. Statistical analyses were performed using two-sided unpaired Student’s *t*-test for data on **b**,**c**,**e**,**f**,**I–o** or Mann–Whitney *U-*test with multiple comparison (Holm–Šídák method) for **k**. **q**, Overlayed markers (CD19, CD4, T-bet and 4,6-diamidino-2-phenylindole (DAPI)) in a HC-Ags and patients with pRD-Ag. Magnification ×10. Follicular (Fo) and extrafollicular (EF) areas are indicated that were used for higher magnifications. **r**, T-bet expression by Fo and EF areas. Magnification ×60. **s**, T-bet expression in B cells by Fo and EF areas. Arrow shows T-bet^+^ B cells. Magnification ×60. Source data

As a clinically established hallmark of immune dysregulation^32^, we documented substantial expansion of CD19^hi^CD21^lo^ B cells in pRD (Fig. 2a,b), which were equally distributed among CD38^int^, CD38^−^ and CD27^+^ B cells (Fig. 2c). The CD11c^hi^CXCR5^lo^ population, resembling murine age-associated B cells (ABCs)^33^, was also present at a higher frequency in pRD and were equally enriched in the CD38^int^, CD38^−^ and CD27^+^ compartments (Fig. 2d–f). Although both HC and pRD ABCs contribute to a smaller fraction of total B cells than CD19^hi^CD21^lo^ cells, they fully overlap with the latter, indicating that ABCs are part of the CD19^hi^CD21^lo^ fraction (Fig. 2g), hence, they expand in parallel in pRD (Fig. 2h).

In addition to these surface markers, transcription factor T-bet serves an ultimate marker for ABCs^33,34^. T-bet was indeed expressed at a higher level in pRD B cells than in HCs (Fig. 2i and Extended Data Fig. 4e). Although CD21^lo^CD11c^−^ and CD21^lo^CD11c^+^ cells were more abundant in pRD than in HCs (Fig. 2j,k) and T-bet expression was increased toward the latter, it was equally elevated in HCs and pRD, indicating that T-bet is induced at similar level in the corresponding populations of healthy individuals or patients (Fig. 2l,m). By assessing T-bet in CD38^int^, CD38^−^, CD27^+^ and DN B cells, we found increased expression in earlier B cell stages (CD38^int^ and CD38^−^) in pRD, whereas its levels were comparable in CD27^+^ and DN B cells with those of HCs (Extended Data Fig. 4f–h) in pRD when measured.

In addition, FcRL4, FcRL5, CD85j and CXCR3 ABC-specific marker expressions were higher in total B cells (Fig. 2n) and CD38^int^, CD38^−^ and CD27^+^ populations (but not in DN cells) in pRD compared to HCs (Extended Data Fig. 4i). Of note, CD95 expression was increased, whereas B cell-activating factor receptor (BAFF-R) expression was decreased in total B cells (Fig. 2o,p) and in each individual subset (Extended Data Fig. 4j,k) in pRD compared to HCs.

**Extended Data Fig. 5:**
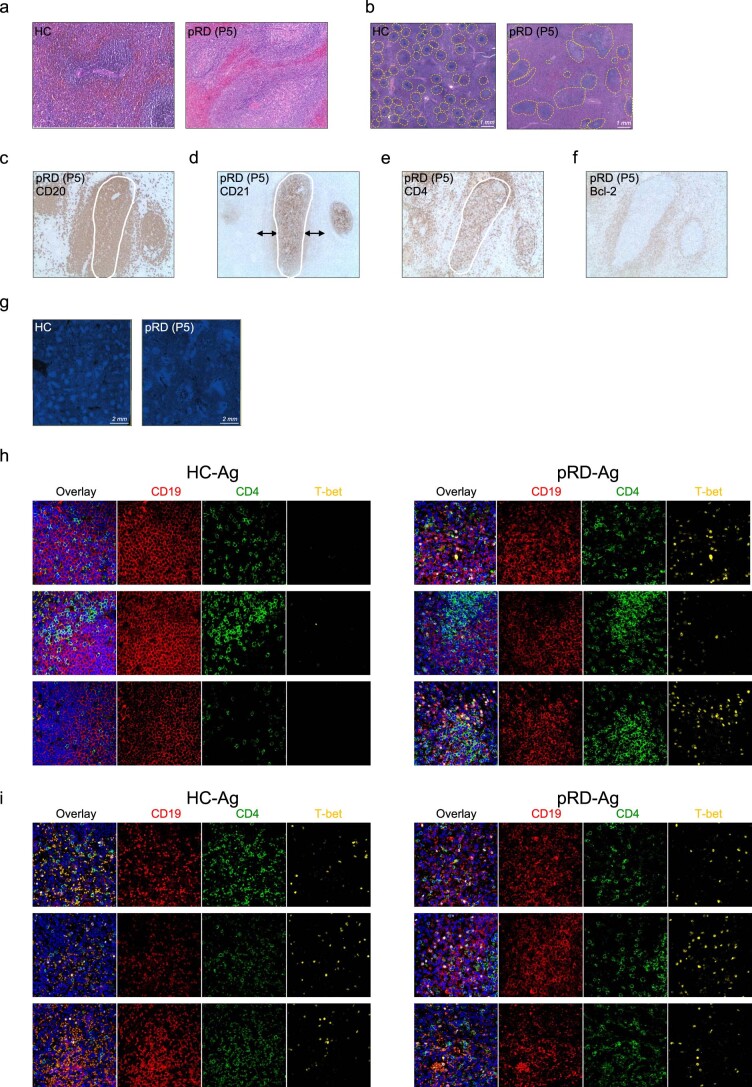
Spleen pathology. **(a)** Hematoxylin and eosin staining of spleen sections from a HC and a pRD-Ag subject. 40x magnifications. **(b)** Hematoxylin and eosin staining of spleen sections from a HC and a pRD-Ag subject. Follicles are depicted with yellow dashed line. 10x magnifications. **(c-f) Chromogenic staining for CD20 (c), CD21 (d), CD4 (e) and Bcl-2(f) in a pRD-Ag subject**. 50x magnifications. **(g) Cellular distribution shown by DAPI (nucleus) staining by confocal microscopy**. 10x magnification. **(h-i) Representative multi-color immunofluorescence images for T-bet expression in follicular (Fo) (h) and extrafollicular (EF) (i) areas**. Overlay shows CD19 (red), CD4 (green), T-bet (yellow) and DAPI (blue); and CD19 (red), CD4 (green) and T-bet (yellow) expressions are shown separately. 60x magnification. All images were obtained from the spleen sections of a single experiments performed simultaneously for a HC-Ag and a pRD-Ag (P5) subject. Source data

Examination of the spleen biopsy sample of a pRD patient (P5) confirmed white pulp hyperplasia and the presence of previously described non-necrotic epithelioid granulomas^35^, revealed remarkable reactive and giant follicles, marginal zone hyperplasia and periarteriolar T-zone hyperplasia, which scored high (12) in comparison to what observed in a previous cohort with common variable immunodeficiency and splenectomy^36^ (Extended Data Fig. 5a,b). Lymphoid hyperplasia was notable for expansion of B cells, especially CD21^lo^ cells outside the germinal centers (Extended Data Fig. 5c,d) along with follicular helper T (T_FH_) cell accumulation (Extended Data Fig. 5e,f). Compared to the HC, spleen follicles from P5 were hyperplastic and irregularly shaped and they occupied a larger proportion of the splenic tissue (Extended Data Fig. 5g). Notably, we detected T-bet^+^ B cells in the follicular areas of the patient, whereas they were absent in those of the HC (Fig. 2q–s and Extended Data Fig. 5h). Although T-bet^+^ B cells were detected in the extrafollicular regions of both P5 and the HC, they were more abundant in the patient (Fig. 2q–s and Extended Data Fig. 5i). Thus, expansion of T-bet^+^ B cells in the circulation, follicular and extrafollicular spaces is a unique feature of pRD and represents an additional marker of B cell dysregulation.

### Restricted BCR repertoire diversity

**Fig. 3:**
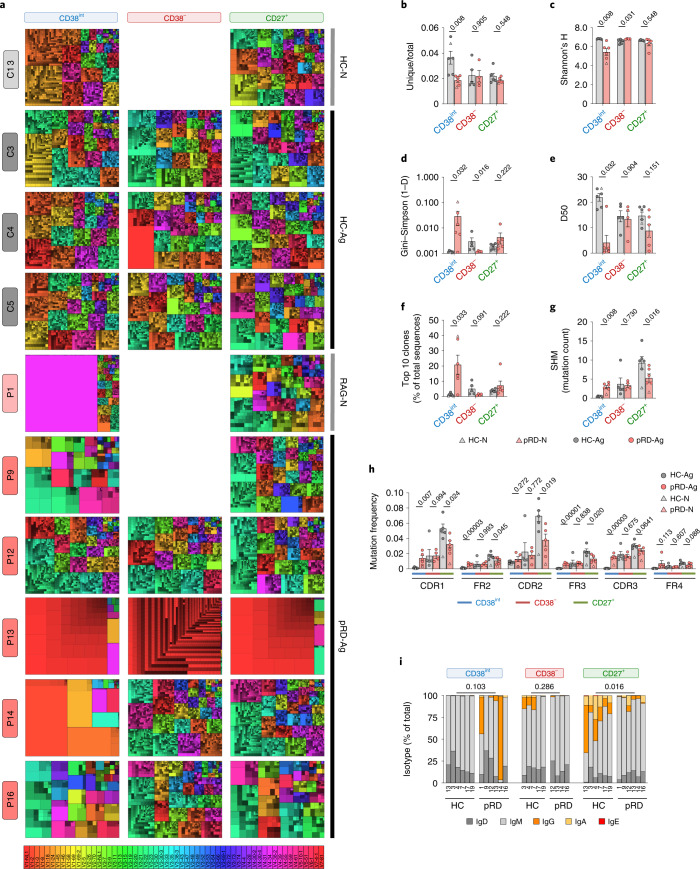
BCR repertoire characteristics in patients with pRD. **a**, Treemap representation of the diversity and clonality of immunoglobulin heavy-chain repertoires.Each rectangle represents an immunoglobulin heavy-chain clonotype and the size of rectangles is proportional to the relative frequency of each clone in the entire repertoire. Distinct clones sharing the same V_H_ gene are displayed using altered brightness of the same hue indicated by the color scale. Thick borders and colors assign clones into the corresponding V_H_ gene families. Each treemap represents the most abundant 2,500 clones. One antigen-naive healthy infant (HC-N), three representative antigen-exposed HCs (HC-Ag), the antigen-naive infant patient (pRD-N) and the antigen-exposed patients with pRD (pRD-Ag, *n* = 5) are shown. Labels on the left show participant IDs. Labels on the top indicate B cell compartments. No sufficient amount of CD38^**−**^ cells was obtained from the infant donors and P9 for sequencing. **b**, Ratio of unique and total sequence counts. **c**–**e,** Shannon’s H (**c**), Gini–Simpson (1 − D) (**d**) and Diversity 50 (D50) (**e**) diversity indices. **f**, Cumulative frequency of the ten most abundant clones. **g**, Frequency of SHM in the V segment. Bars represent mean ± s.e.m. for each group with the individual values indicated. **h**, Mutation distribution. Graph shows replacement mutation frequencies in CDR and FR regions in CD38^int^, CD38^−^ and CD27^+^ B cells. **i**, Isotype distribution. Stacked columns show the percentage of each Ig class in each repertoire by individuals and B cell compartments. Numbers depict participant IDs. Data for each analysis were obtained from HC-Ags (*n* = 5), HC-N (*n* = 1), pRD-Ag (*n* = 5) and pRD-N (*n* = 1). Statistical analyses were performed only on individuals with antigens (HC-Ag versus pRD-Ag) using a Mann–Whitney *U-*test. For **i**, unswitched (IgD + IgM) and switched (IgG + IgA + IgE) sequences were compared. Source data

**Extended Data Fig. 6:**
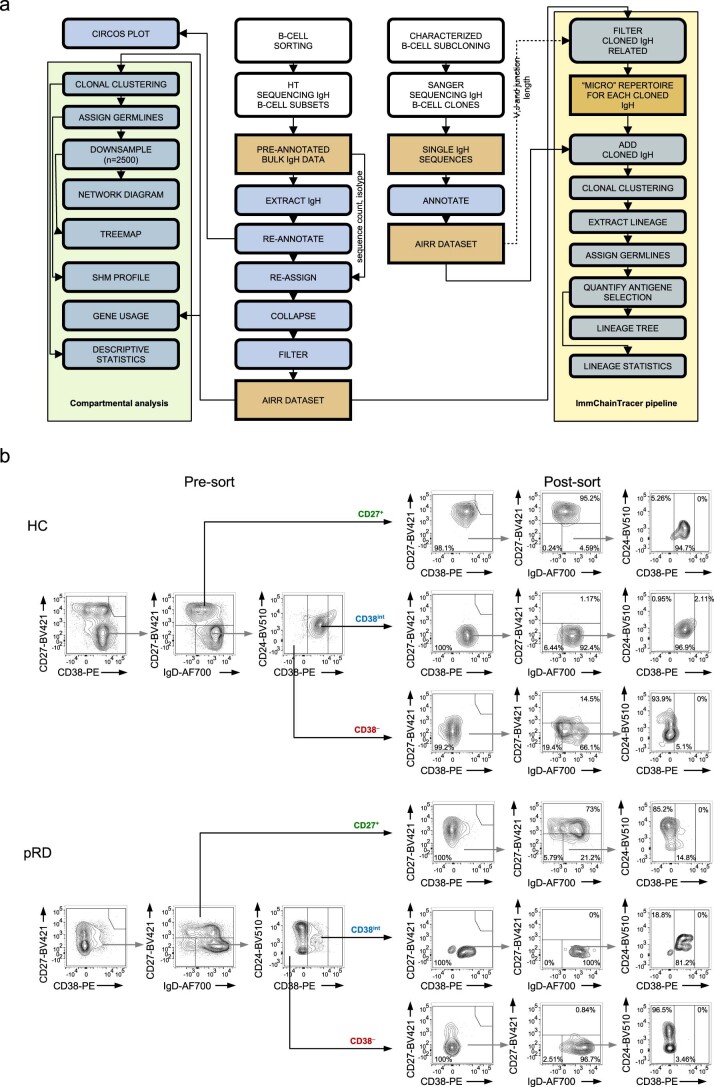
Repertoire analysis pipeline and B cell sorting. **(a) Repertoire analysis pipeline**. See Methods for details. **(b) Gating and sort purity of batch sorted CD38**^**int**^**, CD38**^**−**^ **and CD27**^**+**^ **B cells**. Gating is shown for a HC-Ag and a pRD-Ag subject analyzed parallel in the same experiment, and are representative of six independent experiment, HC-Ag (n = 6), pRD-Ag (n = 5) and pRD-N (n = 1).

**Extended Data Fig. 7:**
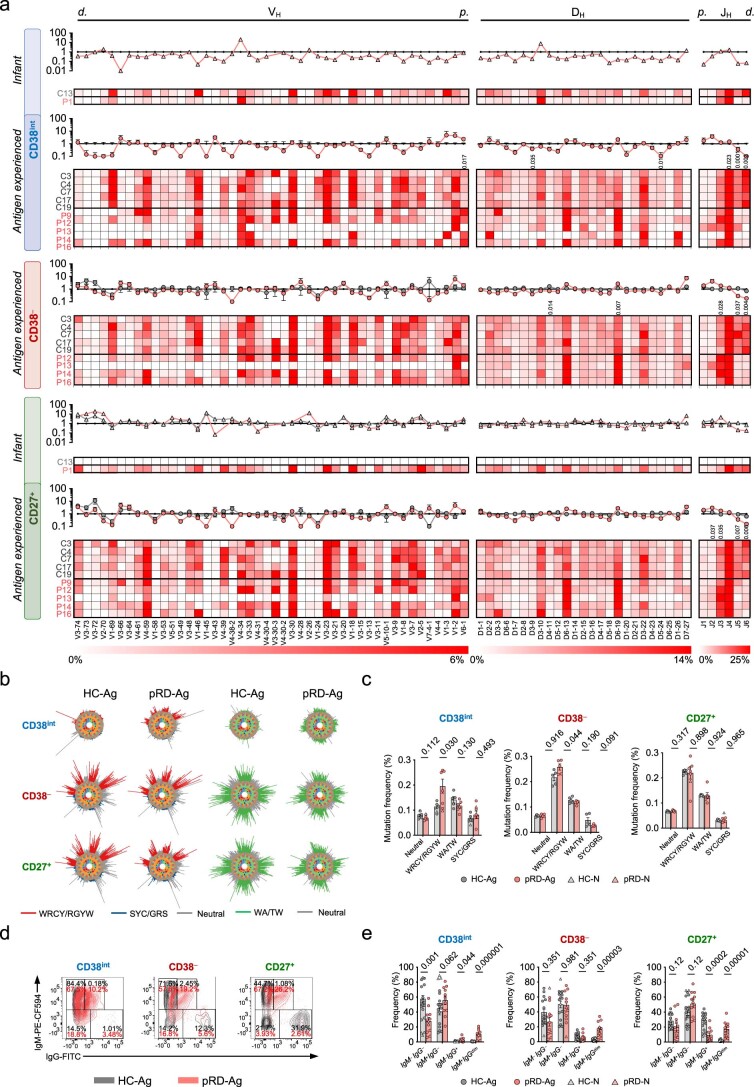
BCR repertoire. **(a) IGH-V-D-J gene usage from CD38**^**int**^**, CD38**^**−**^ **and CD27**^**+**^ **B cells**. Heat maps represent the frequency of each V_H_, -D_H_ and -J_H_ gene by subjects, HC-N (n = 1), HC-Ag (n = 5), pRD-N (n = 1), pRD-Ag (n = 5-6) shown as mean±s.e.m. with individual values. Subject IDs are depicted on the left side of each heat map; and compartments as CD38^int^, CD38^**−**^ and CD27^+^ B cells depicted with blue, red, and green on the left. Ig V_H_, -D_H_ and -J_H_ genes are listed on the bottom by their chromosomal order. Color scales represent gene frequencies for each family separately. *d*.: distal genes, *p*.: proximal genes. Statistical analysis was performed using two-sided unpaired *t*-test with multiple comparison (Holm-Šídák method) to compare V_H_, -D_H_ and -J_H_ gene usage between HC-Ag and pRD-Ag donors and P values are indicated when reached significance. Plot inserts shows changes in gene usages in CD38^**−**^ and CD27^+^ cell compartments compared to the values of HC-Ags in CD38^int^ compartment (horizontal black line) for HCs (gray) and pRD patients (red), infant donors (triangles), and antigen-experienced donors (circles). **(b) “Hedgehog” plots – targeted hot- and coldspot sequence motifs**. Frequencies of WRCY/RGYW (red), SYC/GRS (blue), WA/TW (green) and neutral (gray) mutations are visualized. **(c) Enumeration of different SHM hot- and coldspots in CD38**^**int**^**, CD38**^**−**^ **and CD27**^**+**^ **B cells**. Graph shows mutation frequency in different SHM hot- and coldspots from HC-N (n = 1), HC-Ag (n = 5), pRD-N (n = 1), pRD-Ag (n = 5) shown as mean±s.e.m. with individual values. **(d) IgG surface expression**. Contour plots show the surface expression of IgG and IgM of a representative HC-Ag (dark gray) and a pRD-Ag patient (red) overlayed from their CD38^int^, CD38^−^ and CD27^+^ B cells separately. **(e) Frequency of B cells based on IgM and IgG surface marker expression**. Graphs show frequency of IgM^+^ IgG^−^, IgM^+^ IgG^dim^, IgM^−^ IgG^+^, IgM^−^ IgG^−^ from CD38^int^, CD38^−^ and CD27^+^ B cells and shown as mean±s.e.m. with individual values. Statistical analysis was performed on Ag-experienced subjects using Mann–Whitney *U* test. Data for **d-e** was obtained from HC-N (n = 3), HC-Ag (n = 15), pRD-N (n = 1), pRD-Ag (n = 11). Source data

The prominent alterations in the subset distribution and activation of B cells in pRD prompted us to evaluate the BCR repertoire composition in sorted CD38^int^, CD38^−^ and CD27^+^ populations (Extended Data Fig. 6a,b and Supplementary Table 3). As visualized on treemaps, CD38^int^ B cells from pRD expressed oligoclonal repertoires with prominent expansion of 1 to 5 specific V_H_ gene families (Fig. 3a). These expanded VH families were usually dominated by a few expanded unique clones. In contrast, CD38^−^ and CD27^+^ repertoires were more similar to those of HCs except P13, whose repertoires were dominated VH1-2 genes. Restricted diversity with oligoclonality of the CD38^int^ subset in pRD was further supported by the low ratio of unique to total sequences, lower Shannon’s H and increased Gini–Simpson indices in the CD38^int^ BCR repertoires of the patients, compared to HCs (Fig. 3b–d). Of note, fewer than 5% of the unique sequences accounted for 50% of the total CD38^int^ repertoires in patients P1, 9, 13, 14 and 16 (Fig. 3e) and the most common ten clones comprised a substantial fraction of their CD38^int^ repertoires (mean ± s.e.m., 17.13 ± 6.071), in contrast to HCs (mean ± s.e.m., 1.45 ± 0.39) (Fig. 3f). Together, these data demonstrate decreased diversity and expansion of certain clones in the CD38^int^ repertoire of patients with pRD, whereas their CD38^−^ and CD27^+^ compartments seemed to be substantially more diverse. Regarding V_H_, D_H_ and J_H_ gene segment utilization, we found diversified repertoires in the patients (except in P13) (Extended Data Fig. 7a). In addition, despite the high plasma titer of IgM-type 9G4 antibodies in patients, we did not find elevated frequency of V_H_4–34-carrying clones in the pRD-Ag repertoires; however, it was expanded in the patient with pRD-N (20.92%). As previously published^14^, distal J_H_5 and J_H_6 gene segments were significantly less frequent in all three compartments repertoires reflecting the *RAG* activity impairment in the patients (Extended Data Fig. 7a).

Hence, in connection to their altered phenotype and activation of pRD B cells we identified severe diversity restrictions in the pre-immune repertoire of the patients.

### Aberrant somatic diversification

Next, we identified a uniformly increased level of somatic hypermutation (SHM) in the CD38^int^ repertoire of patients with pRD-Ag despite that these cells should express the germline version of their V segments with nearly no mutations, as found in the HCs (Fig. 3g). This finding indicates that although CD38^int^ B cells in patients with pRD were phenotypically defined as mature naive resting B cells (IgD^+^CD21^+^CD24^int^CD27^−^CD38^int^), some may represent Ag-experienced B cells and cannot be considered bone fide naive cells. Of note, continuum expression of CD27 did not segregate naive and memory B cells with high confidence in pRD (Extended Data Fig. 3g; CD27-IgD plots). Notably, although the patient with pRD-N already acquired some SHM in the CD38^int^ compartment, it did not reach the level seen in patients with pRD-Ag, suggesting that the unusually early-onset SHM of CD38^int^ B cells is a progressive phenomenon in pRD. Notably, although the CD38^−^ compartment represent phenotypically naive B cells (IgD^+^CD27^−^), they displayed a similarly elevated level of SHM whether they were obtained from HCs or patients with pRD, suggesting that despite their different abundance in the peripheral blood of HCs and pRD, they share comparable mutational diversification characteristics. Although SHM was elevated in the CD38^int^ and CD38^−^ compartments of patients, its level remained lower in their CD27^+^ repertoire compared to HCs, indicating impaired mutational diversification and affinity maturation in pRD. As expected, SHM levels in the CD27^+^ repertoire of both the infant patient and the age-matched control (pRD-N and HC-N, respectively) were similarly lower than in Ag-experienced individuals. SHM frequency was substantially higher in the complementarity-determining regions (CDRs) than in the framework regions (FRs) of the pRD CD38^int^ B cells, suggesting antigen-driven selection (and remained close to zero in the CDRs and FRs of HCs) (Fig. 3h). This pattern was preserved in the CD38^−^ and CD27^+^ compartments; however, SHM frequency in the latter did not reach that of corresponding HCs, further confirming the absence of proper memory response in pRD (Fig. 3h). Of note, mutations were more frequent in the activation-induced cytidine deaminase (AID) WRCY/RGYW hotspot of the CD38^int^ and CD38^−^ compartments in pRD than in HCs (Extended Data Fig. 7b,c).

Regarding immunoglobulin class-switch recombination (CSR), we detected an abundance of switched transcripts in CD38^int^ repertoires in pRD although with substantial inter-individual variability, whereas it remained negligible in HCs (Fig. 3i). This implies the unforeseen inclusion of antigen-experienced B cells in the phenotypically naive compartment in pRD. In connection to this, we detected unusual co-presence of IgM and IgG on the surface of the patients’ B cells (Extended Data Fig. 7d,e). On the other hand, CSR was significantly lower in the CD27^+^ repertoires of patients compared to HCs (Fig. 3i).

In summary, we found somatic diversification in the CD38^int^ compartment and subpar SHM and CSR in the CD27^+^ compartment of the patients with pRD; hence, these findings provide additional evidence for early widespread and dysregulated B cell activation in pRD, with predisposition to impaired humoral effector function, consistent with our previous findings of decreased fraction and number of SM B cells.

### Defective tolerance that worsens with age

**Extended Data Fig. 8:**
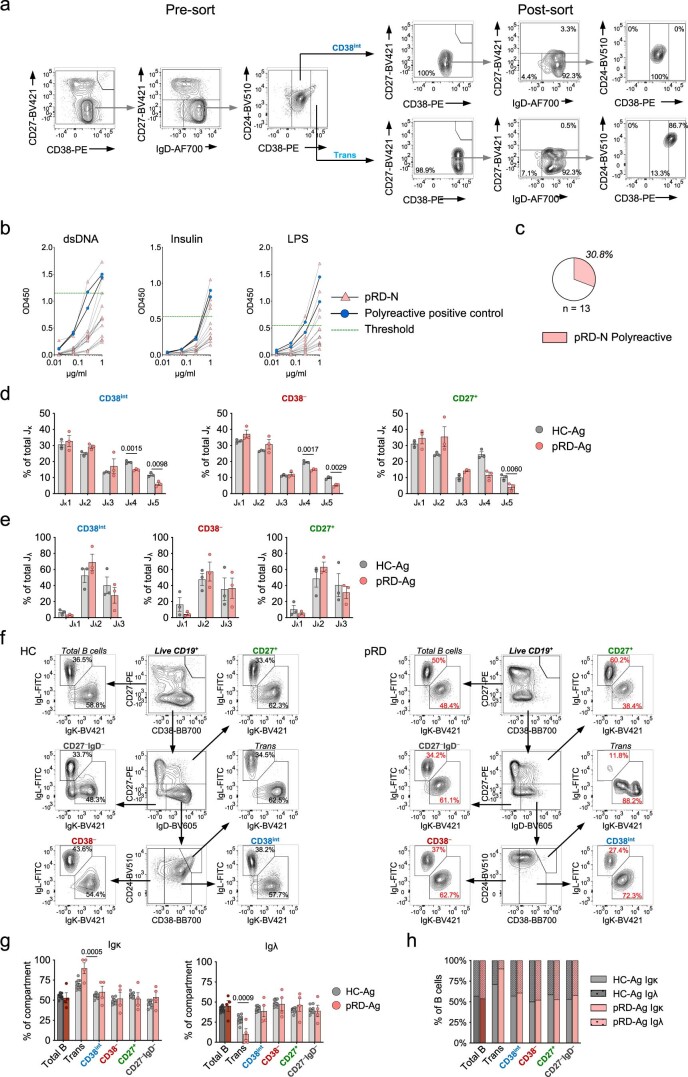
Impaired central B cell tolerance. **(a) Gating and sort purity of index single-cell sorted new emigrant transitional and CD38**^**int**^ **B cells**. Pre-sort plots show the original composition of B cells in the samples before single-cell sort. Post-sort plots show the phenotype of the 46 or 94 new emigrant or CD38^int^ B cells index single-cell sorted into 96-well plate. Gating is shown for a representative HC-Ag subject, experiment was performed for HC-Ag (n = 3), pRD-Ag (n = 5) and pRD-N (n = 1). **(b) Binding of Abs from new emigrant transitional B cells to dsDNA, insulin and LPS**. Abs cloned from new emigrant transitional B cells of pRD-N patient (P1) were tested for anti-dsDNA, insulin and LPS reactivity in serial dilution. Thick black lines with blue circles show binding of the two positive controls used in each assay to determine threshold for positive reactivity (mean of positive controls minus 2 SD at 1.0 µg/ml). Threshold for positive reactivity is shown with green dashed line. **(c) Frequency of polyreactive new emigrant transitional B cell clones**. The frequency of non-polyreactive (open area) and polyreactive (filled area) clones is summarized in pie chart. Abs were considered polyreactive when they recognized at least two of the three antigens tested. The total number of clones tested is indicated. **(d-e). Frequency of J**_**κ**_ **(d) and J**_**λ**_ **(e) light chain gene segments in repertoires**. Data are shown as mean±s.e.m. with individual values for each gene segments in CD38^int^, CD38^−^ and CD27^+^ B cell repertoires of pRD patients (n = 3) and HCs (n = 3). Statistical analysis was performed using Two-sided unpaired *t*-test with multiple comparison. **(f) Gating for kappa and lambda light chain expression**. Representative plots are shown for a HC-Ag and pRD-Ag patient. **(g) Kappa and lambda light chain expression**. Data are shown as mean±s.e.m. with individual values. Statistical analysis was performed using two-sided unpaired Student’s *t*-test. **(h) Ratios of kappa:lambda light chain expression**. Graph shows the relative ratio of kappa:lambda light chain expression as means. Data for **f-h** was obtained from a single experiment performed simultaneously on HC-Ags (n = 12) and pRD-Ag (n = 4) subjects. Source data

Frequencies of the polyreactive clones in new emigrant (NE) transitional and CD38^int^ B cell compartments are measures of the efficiency of central and peripheral B cell tolerance, respectively^6^. Thus, we assessed the abundance of polyreactive clones by analyzing the reactivity of recombinant antibodies cloned from single-sorted NE and CD38^int^ B cells (Extended Data Fig. 8a). Due to the scarcity of circulating NE cells in the patients with pRD-Ag, we managed to assess the central tolerance efficiency only in the asymptomatic infant (P1). We detected remarkably higher frequency of polyreactive clones compared to published age-matched individuals with functional central tolerance^37^ (Extended Data Fig. 8b,c). We also found decreased usage of distal J_κ_ gene segments (J_κ_4 and 5) in the patients compared to HCs (Extended Data Fig. 8d), (but not in the distal J_λ_ gene segments (J_λ_3); Extended Data Fig. 8e). Besides impaired V(D)J recombination, this observation also illustrates defective receptor editing providing additional evidence for impaired central tolerance in pRD. In contrast, we did not find significant disturbance in the proportion of κ and λ light-chain expression (another potential marker of receptor editing) in either of the B cell compartments of the patients, except transitional cells (Extended Data Fig. 8f–h).

**Fig. 4:**
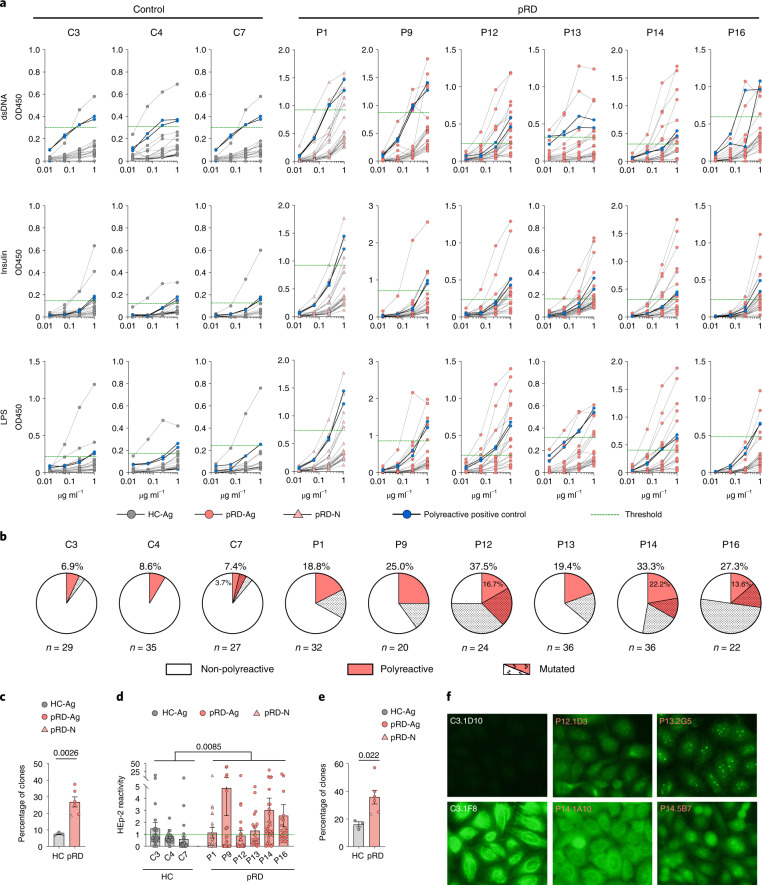
Impaired peripheral B cell tolerance in patients with pRD. **a**, Polyreactivity of CD38^int^ B cells. Antibodies cloned from CD38^int^ B cells were tested for anti-double-stranded DNA (dsDNA), insulin and lipopolysaccharide (LPS) reactivity in serial dilution. Binding of 20 randomly selected clones is shown on each graph. Thick black lines with blue circles show binding of the two positive controls used in each assay to determine the threshold for positive reactivity (mean of positive controls minus 2 s.d. at 1.0 µg ml^−1^). Threshold for positive reactivity is shown by a green dashed line. **b**, Frequency of polyreactive clones by individuals. Pie charts depict the frequency of non-polyreactive (white) and polyreactive (red) clones for each individual and the percentage of polyreactive clones are shown. The fraction of sequences carrying at least two mutations compared to their corresponding germline versions is depicted by dotted patterns. **c**, Frequency of polyreactivity. **d**, HEp-2 reactivity of CD38^int^ B cell clones. Cloned antibodies were tested in 5.0 µg ml^−1^ concentration for anti-HEp-2 cell line lysate with the two positive controls used in each assay. Blank corrected absorbance values were normalized with the mean of positive controls minus 2 s.d. and values above 1 (green dashed line) were considered positive for HEp-2 reactivity. **e**, Frequency of HEp-2 reactivity. For **c**–**e** data are shown as mean ± s.e.m. with individual values and statistical analyses performed on individuals with antigens (HC-Ag versus pRD-Ag) using a two-sided unpaired Student’s *t*-test. Antibodies were cloned and tested from HC-Ags (*n* = 3), pRD-N (*n* = 1) and patients with pRD-Ag (*n* = 5). The total number of B cell clones tested is indicated for each individual in **b**. **f**, HEp-2 immunofluorescence. Cloned antibodies were used in HEp-2 immunofluorescence assays to show target antigen distribution. Representative images for negative and positive stainings are shown from one HC-Ag and three pRD-AG individuals. Individual and antibody clone IDs are shown. Magnification ×16. Source data

Next, we found elevated fraction of polyreactive clones in the CD38^int^ compartment in all patients compared to HCs, demonstrating a uniform peripheral tolerance defect in pRD (Fig. 4a–c and Supplementary Table 3). We noted significant difference (*P* = 0.031; Student’s *t*-test) in the frequency of polyreactive clones between children (*n* = 3) and adults (*n* = 3) with pRD (21.1 ± 2.0% mean ± s.e.m.; range 18.8–25.0% and 32.7 ± 3.0% mean ± s.e.m.; range 27.3–37.5%, respectively). Of note, a fraction of the analyzed clones from P12, P14 and P16 carried mutations in their V segment, indicating ongoing somatic diversification and therefore, cannot be considered as resting mature B cells. Nevertheless, the proportion of polyreactivity in unmutated clones was still uniformly higher in all patients compared to HCs, confirming the profound peripheral tolerance defect. HEp-2 reactivity of the expressed clones confirmed peripheral tolerance impairment in pRD (Fig. 4d,e). In addition, HEp-2 cell-based IFA revealed distinct binding of the polyreactive clones (cytoplasmic fibrillar filamentous (P12.1D3), multiple nuclear dots (P13.2G5), cytoplasmic dense speckled (P14.1A10) and homogenous nucleolar (P14.5B7)), suggesting that although they are polyreactive, they bear preferential self-specificities (Fig. 4f).

In summary, these data demonstrate that B cell tolerance is defective in pRD, it worsens with age and likely with chronic antigen exposure.

### Diversification and persistence of polyreactive clones

**Fig. 5:**
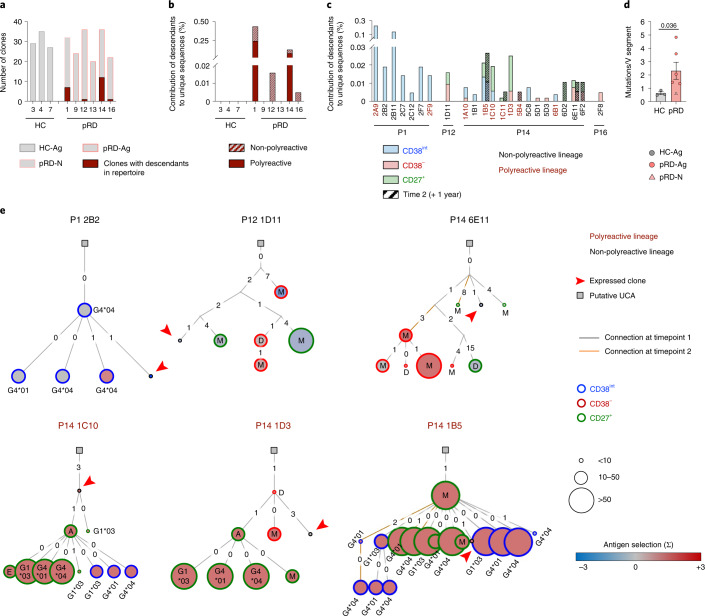
Expansion and diversification of polyreactive clones. **a**, Clones with descendants in total repertoire. Graph shows the number of the in vitro expressed clones by donors (gray bars). Number of clones with identified descendants in total repertoire are depicted with deep red. Data are shown from HC-Ags (*n* = 3), pRD-N (*n* = 1) and patients with pRD-Ag (*n* = 5) with individual IDs indicated. **b**, Contribution of descendants to repertoire. Fraction of the descendants of clones in total repertoires are shown by individuals. Descendants of the polyreactive and non-polyreactive clones are depicted with filled and striped bars, respectively. **c**, Compartmental distribution of the descendants. The contribution of the descendants of each clone to the total repertoires are shown in the indicated individuals by CD38^int^ (blue); CD38^**−**^ (red) and CD27^+^ (green) compartments. Clone IDs are shown; deep red and black represents polyreactive and non-polyreactive clones, respectively. Striped bars for P14 represent clones identified in repertoires obtained 1 year later after isolation of original clones. Clones with shared descendants in the two time points are indicated with bold. **d**, Single nucleotide mutations in clones. Average number of mutations in the V segment by individuals are shown as mean ± s.e.m. for HCs (*n* = 3), pRD-Ag (*n* = 5) and pRD-N (*n* = 1) with individual values depicted. Statistical analyses were performed on individuals with antigens (HC-Ag versus pRD-Ag) using a Mann–Whitney *U*-test. **e**, Phylogenic lineage trees of expanded clonotypes. Immunoglobulin trees represent expansion of individual clonotypes by their SHM profile. Putative unmutated common ancestors (UCAs) are depicted by squares, arrowhead indicates the original in vitro expressed sequence, descendants are shown by CD38^int^ (blue), CD38^**−**^ (red) or CD27^+^ (green) compartments and the color of the nodes indicates antigen selection strength (blue to deep red). Size of the nodes represent sequence abundance. The number of the nucleotide difference between adjacent sequences and the isotype of each sequence are shown. Lineages derived from polyreactive or non-polyreactive CD38^int^ clones are depicted with deep red or black, respectively. Orange line indicates descendants from second time point for P14. Source data

Because our findings imply that polyreactive B cell clones escape both central and peripheral tolerance checkpoints and survive, we aimed to evaluate their trajectories and determine whether they reach effector compartments. Therefore, we traced each expressed sequence and their related descendants in the CD38^int^, CD38^−^ and CD27^+^ repertoires. Notably, we identified related clones in the CD38^int^ BCR repertoires of P1, P12, P14, P16 (*n* = 24.8 ± 5.4 clones per donor) (Fig. 5a). Two of these patients (P1 and P14) had persistent polyreactive and non-polyreactive clones that contributed to the entire sequence pool at a remarkable level (Fig. 5b). As expected, no related clones were identified in the naive or effector compartments of the HCs. In contrast, we detected simultaneous presence of related clones in CD38^int^, CD38^−^ and CD27^+^ compartments of three patients with pRD-Ag, whereas sister clones were only present in the CD38^int^ compartment of the individual with pRD-N (Fig. 5c). Furthermore, longitudinal analysis of relatedness revealed durable presence of three distinct clones in P14 (1B5, 1C11 and 6E11) 1 year apart, with higher abundance in CD38^−^ or CD27^+^ compartments at the second time point. Collectively, these data indicate that certain polyreactive (and non-polyreactive) CD38^int^ clones persist chronically in pRD.

The rate of SHM in the V region of the patients’ clones was increased compared to those of HCs (Fig. 5d) (similarly to the full CD38^int^ repertoire; Fig. 3g), reflecting activation due to potential antigen exposure. Mutation frequency was higher in patients with pRD-Ag, whereas it remained similar in the infant patient to that of HCs. Constructing phylogenic immunoglobulin lineage trees of related clones (Fig. 5e) revealed remarkably expanded B cell lineages that consisted of 7.59 ± 11.03 (mean ± s.d., range 1–48) individual sequences in the patients. Of note, several descendants expressed switched transcripts such as IgG1*03, IgG4*01, IgG4*04, IgA and IgE, which were more abundant among those of polyreactive clones, suggesting a potential role for polyreactivity in the accelerated somatic diversification. In connection with accumulating SHMs, we detected positive antigen selection in several lineages that was also more frequent in those originated from polyreactive CD38^int^ clones. Notably, largely expanded individual clones likely harbored sequences with stronger antigen selection values and reached the CD27^+^ compartment (P14 1C10, 1B5 and 1D3).

These data provide experimental evidence for the prominent B cell clonal expansion with notable intralineage somatic diversification by CSR and SHM in pRD. Descendants of several clones were simultaneously present in the CD38^−^ or CD27^+^ compartments, revealing intercompartmental expansion and persistence. Together, these findings demonstrate how polyreactive lineages persist, expand and diversify in pRD after escaping inefficient peripheral tolerance mechanisms.

### Intralineage diversification and clonal connectivity

**Fig. 6:**
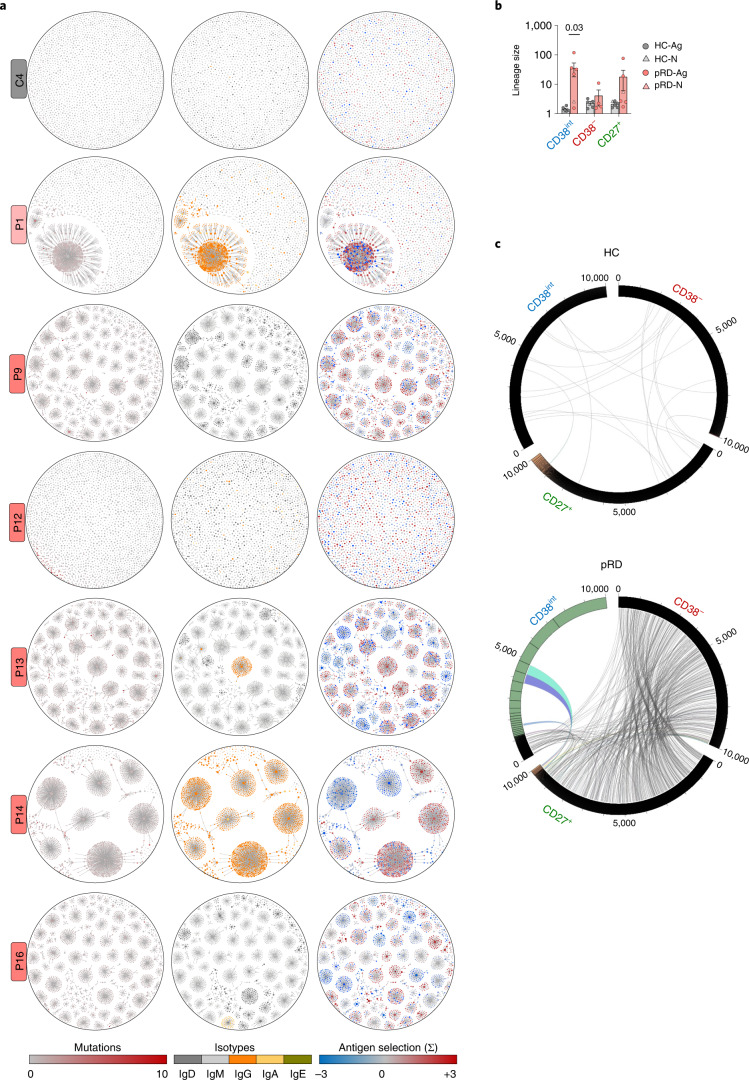
Clonal connectivity and diversification. **a**, Clonal expansion with somatic hypermutation, isotype switches and antigen selection in CD38^int^ compartment. Repertoire data were randomly downsampled to 2,500 unique sequences from CD38^int^ compartments of each donor and plotted on lineage network diagrams. Nodes represents unique sequences, where the size of the nodes is proportional to the abundance of the given sequence in the repertoire on a logarithmic scale and lines depict clonal relatedness. Therefore, nodes in each cluster belong to the same clonotype and differ in mutations in their V segments. Accumulation of mutations (left), isotypes (middle) and antigen selection values (right) are shown for each clone as indicated below the diagrams. One representative adult HC-Ag, the infant pRD-N and the patients with pRD-Ag (*n* = 5) are shown, labels on the left indicate individual IDs. **b**, Clonotype size (intra-compartmental clonal connectivity). Average number of clonal connections by repertoires (CD38^int^, CD38^**−**^ and CD27^+^) Data are shown as mean ± s.e.m. for HC-N (*n* = 1), HC-Ag (*n* = 5), pRD-N (*n* = 1) and pRD-Ag (*n* = 5). Mann–Whitney *U*-test was performed on individuals who were Ag-experienced. **c**, Clonal sharing between CD38^int^, CD38^−^ and CD27^+^ repertoires. The 10,000 randomly selected sequences from each repertoire were arranged on circular plots from a representative HC-Ag and patients with pRD-Ag. Numbers indicate sequence IDs; segmentation of rings indicates clonal size. Clones belonging to the same lineages in distinct compartments are connected depicting clonal connections. Colored connection lines indicate lineages accounting for sequences found in the largest clones up to 20% of total sequences. Plots are representative of a HC-Ag and pRD-Ag. Data from additional investigated individuals are shown in Extended Data Fig. 9c. Source data

**Extended Data Fig. 9:**
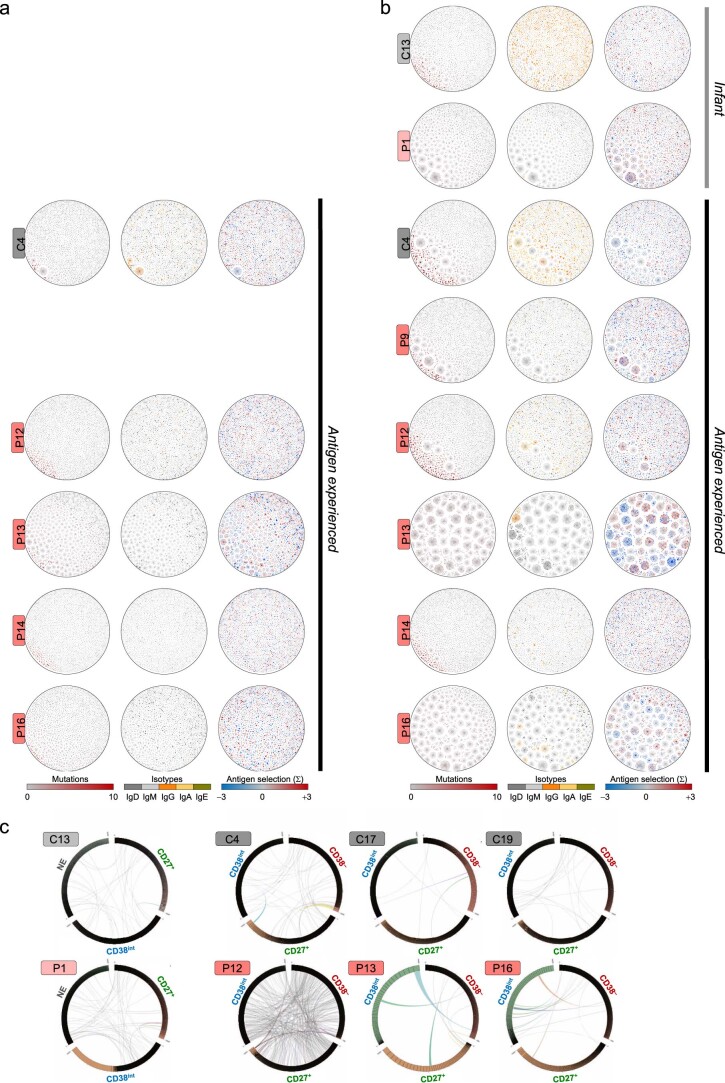
Clonal correlations and diversification. **(a,b) Clonal expansion with somatic hypermutation, isotype switches and antigen selection in CD38**^**−**^ **and CD27**^**+**^ **B cell compartments**. Lineage network diagrams were generated for CD38^−^ **(a)**, and CD27^+^ B cell **(b)** compartments as described in Fig. 6a and Methods. One representative HC-Ag is shown from n = 5, and all other sequenced subjects are shown (HC-N, n = 1, pRD-N n = 1 and pRD-Ag n = 5). **(c) Clonal sharing between CD38**^**int**^**, CD38**^**−**^ **and CD27**^+^ **repertoires**. Clonal relatedness is shown as in Fig. 6c for the all the additional investigated subjects not shown in Fig. 6c. Note that no sufficient amount of CD38^−^ cells was available from either infant donor, however, repertoire analysis was possible from new emigrant transitional cells (NE), therefore, circular visualization plots were generated for the infant subjects on the NE, CD38^int^ and CD27^+^ compartments.

Prompted by the evidence that polyreactive B cells clonally expand, accumulate in CD38^−^ and CD27^+^ compartments and show intralineage diversification, we analyzed clonal trajectories at the full BCR repertoire level. In line with the previously observed oligoclonality (Fig. 3a–f), we detected remarkable clonal expansion in CD38^int^ compartments of five patients (Fig. 6a,b), whereas those of HCs showed very uniform networks with each node of similar size, reflecting no intraclonal expansion. Notably, clonal expansion in pRD was not homogenous as indicated by lineages with numerous different related sequences (as opposed to lineages with single large nodes) and diversification occurred by SHM and CSR as already observed at the single-clone level (Fig. 5e). The symmetric structure of the expanded lineages with radial projections of the diversified sister clones in patients indicates neutral drift-like clonal microevolution and likely the absence of specific antigen-driven positive selection; however, in some cases asymmetric branches containing enlarged nodes extruded from the core structure. These likely represent selective expansion of particular sequences due to possible positive selection pressure. Indeed, linking antigen selection strength values to the sequences revealed positive selections associated to several asymmetric branches with enlarged clonal size. In contrast, clonal expansion was less pronounced in the CD38^−^ and CD27^+^ compartments and mainly observed only in P13 and P16 (Fig. 6b and Extended Data Fig. 9a,b). Investigation of clonal sharing revealed intercompartmental lineage connections with promiscuous clonal overlap between CD38^int^, CD38^−^ and CD27^+^ compartments in pRD, whereas the connections remained near zero in HCs (Fig. 6c and Extended Data Fig. 9c).

### Enhanced B cell activation signals

**Fig. 7:**
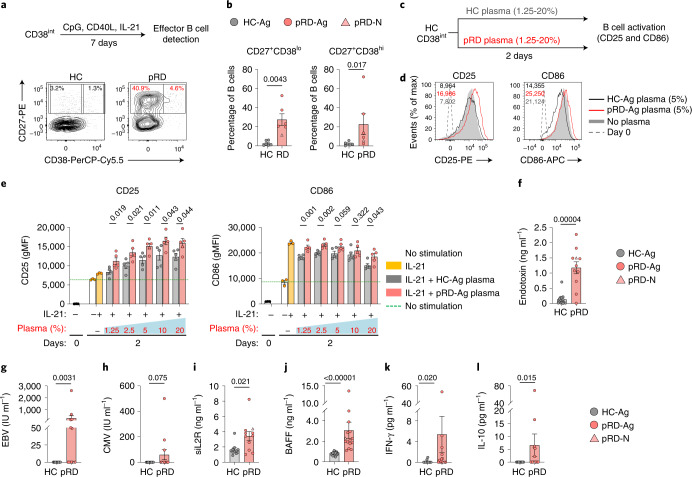
B cell activation signals. **a**, In vitro CD38^int^ B cell differentiation. Sorted CD38^int^ B cells were activated with CpG, CD40L and IL-21. Differentiated cells (CD27^+^CD38^lo^ and CD27^+^CD38^hi^) were quantified on day 7. Experimental design is shown and contour plots are representative of an individual with HC-Ag and a patient with pRD-Ag from six independent experiment. **b**, Enumeration of effector cells. The proportion of CD27^+^CD38^lo^ and CD27^+^CD38^hi^ from HC-Ags (*n* = 6), pRD-N (P1) (*n* = 1) and patients with pRD-Ag (*n* = 5) are shown as percentage of total live B cells in the culture. **c**, Experimental design for testing B cell activation by plasma. Sorted CD38^int^ HC B cells were cultured in vitro in the presence of IL-21 and plasma from either a patient with pRD-Ag (*n* = 5) or independent HC-Ag (*n* = 5) and analyzed on day 2 for CD25 and CD86 expression. **d**, Activation of sorted CD38^int^ B cells with plasma. Surface expression of CD25 and CD86 are shown as overlaid histogram plots depicting representative samples of HC-Ag plasma-stimulated, patient with pRD-Ag plasma-stimulated and unstimulated HC. Dashed line indicated marker expression level on day 0. Expression levels are indicated as gMFIs. **e**, Enumeration of B cell activation. Graphs show expression of CD25 and CD86 at day 0 and 2 days of stimulation with HC-Ags (*n* = 5) or patients with pRD-Ag (*n* = 6) plasma at the indicated plasma concentration in culture medium. Data for **d**,**e** were obtained from a single experiment performed on all plasma samples simultaneously. **f**, Plasma endotoxin levels. HC-Ags (*n* = 13), pRD-N (P1) (*n* = 1) and pRD-Ags (*n* = 10). **g**,**h**, Plasma virus levels. Epstein–Barr nuclear antigen and CMV titers were detected in the plasma of HC-Ags (*n* = 15), pRD-N (P1) (*n* = 1) and pRD-Ags (*n* = 12). **i**, Plasma soluble IL-2R levels. HC-Ags (*n* = 11), pRD-N (P1) (*n* = 1) and pRD-Ags (*n* = 10). **j**, Plasma BAFF levels. HC-Ags (*n* = 20), pRD-N (P1) (*n* = 1) and pRD-Ags (*n* = 14). **k**,**l**, Plasma IFN-γ and IL-10. HC-Ags (*n* = 11), pRD-N (P1) (*n* = 1) and pRD-Ags (*n* = 14). Data are shown as mean ± s.e.m. with individual values depicted on each graph. For statistical analysis Mann–Whitney *U*-test was performed for **b**; and two-sided unpaired Student’s *t*-test was used for **e**–**l** to compare patients with pRD-Ag to individuals with HC-Ag. Source data

Their activated phenotype and prominent expansion imply increased propensity of pRD CD38^int^ B cells for differentiation to effector cells. Indeed, CD38^int^ B cells from patients with pRD-Ag differentiated at higher frequency into CD27^+^CD38^lo^ or CD27^hi^CD38^hi^ in vitro than those of HCs (Fig. 7a,b). Notably, HC CD38^int^ B cells became more activated when cultured with patient’s plasma in vitro, as detected by increased expression of CD25 and CD86 (Fig. 7c–e) compared to HCs. Thus, plasma from patients with pRD represents a unique pathogenic microenvironment that induces rapid and promiscuous B cell activation.

We detected significantly higher endotoxin concentrations in the patients’ plasma compared to HCs (Fig. 7f) and 6 of 13 and 3 of 13 patients tested positive for EBV and CMV, respectively (Fig. 7g,h). The presence of these foreign antigens was associated with highly elevated soluble IL-2R plasma levels in pRD reflecting lymphocyte activation and inflammation (Fig. 7i). In addition, we found higher plasma levels of BAFF in pRD than in HCs (Fig. 7j). Of note, we also measured detectable levels of IFN-γ (Fig. 7k) and IL-10 (Fig. 7l) in the plasma samples of several patients while they remained undetectable in HCs. These data together indicate that pRD establishes a unique milieu that favors promiscuous B cell activation.

### Robust T_FH_ cell function

**Fig. 8:**
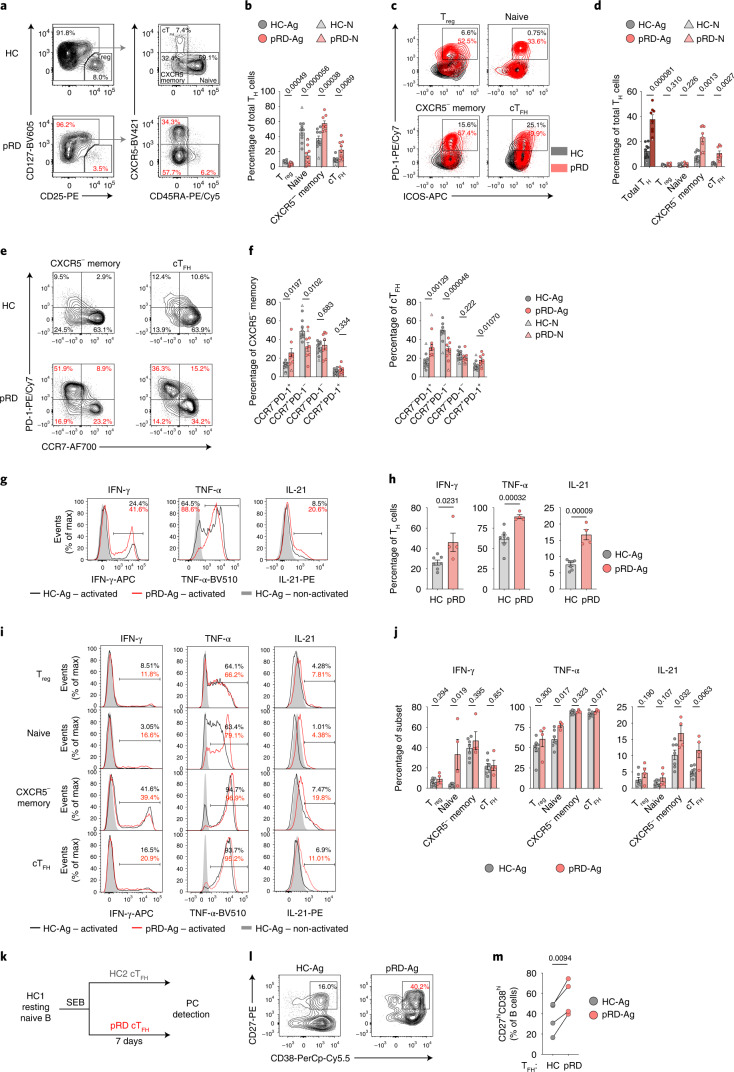
Characterization of T_H_ cells. **a**, Gating for T_reg_, cT_FH_, CXCR5^−^ memory and naive T_H_ cells. T_H_ subsets are shown from a live lymphocyte CD4^+^ gate in representative HC-Ag and a patient with pRD-Ag. **b**, Frequencies of T_H_ subsets. **c**, PD-1^+^ICOS^+^ cells in T_H_ subsets. Overlayed plots show PD-1^+^ICOS^+^ cells by subsets in a representative HC-Ag and an individual with pRD-Ag. **d**, Proportion of PD-1^+^ICOS^+^ T_H_ cells. Graphs show frequencies of PD-1^+^ICOS^+^ cells in total T_H_ cells and by subsets. **e**, Correlation of PD-1 and CCR7 expression. Fraction of CCR7^−^PD-1^+^, CCR7^+^PD-1^−^, CCR7^−^PD-1^−^ and CCR7^+^PD-1^+^ are shown in CXCR5^−^ memory and cT_FH_ cells in a representative HC-Ag and a patient with pRD-Ag. **f**, CXCR5^−^ memory and cT_FH_ populations fractioned by PD-1 and CCR7 expression. Data for **b**,**d**,**f** are shown as mean ± s.e.m. with individual values from HC-N (*n* = 1), HC-Ags (*n* = 10), pRD-N (*n* = 1) and pRD-Ags (*n* = 8). **g**, In vitro IFN-γ, TNF-α and IL-21 production by total T_H_ cells. Data are shown as overlaid histogram plots depicting a representative stimulated individual with HC-Ag and a patient with pRD-Ag; and an unstimulated HC-Ag from live lymphocyte CD4^+^ gate. **h**, Frequencies of IFN-γ, TNF-α and IL-21 positive T_H_ cells. **i**, In vitro IFN-γ, TNF-α and IL-21 production by T_H_ subsets. Data are shown as in **g** by T_H_ subsets. **j**, Frequency of IFN-γ, TNF-α and IL-21 positive cells by T_H_ subsets. Data for **h** and **j** are shown as mean ± s.e.m. with individual values from HC-Ags (*n* = 7) and pRD-Ags (*n* = 4). Data for **g**–**j** were obtained from four independent experiments. For statistical analysis Mann–Whitney *U*-test with Holm–Šídák multiple comparison were performed for **b** and two-sided unpaired Student’s *t*-test for **d**,**f**,**h**,**j** to compare HC-Ag and pRD-Ag. **k**, Approach to assess cT_FH_ function. **l**, Detection of PCs. PCs were identified from the live lymphocyte gate in a representative HC-Ag and an individual with pRD-Ag. **m**, Proportion of PCs in culture. PC frequencies induced by HC-Ag (*n* = 4) or pRD-Ag (*n* = 4). cT_FH_ cells are shown as individual values and statistical analyses were performed using two-sided paired Student’s *t*-test. Data for **l**,**m** were obtained from four independent experiments. Source data

**Extended Data Fig. 10:**
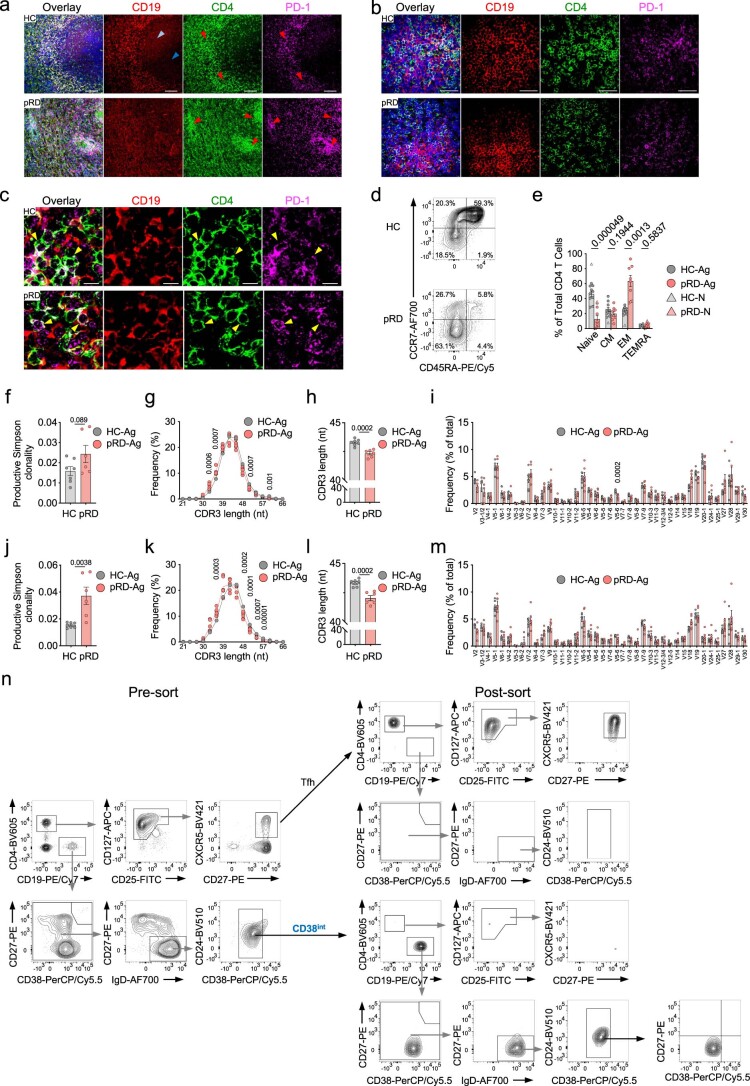
T cells. **(a) Representative multi-color immunofluorescence image of spleen sections**. Overlayed markers are shown as CD19 (red), CD4 (green), PD-1 (violet), and DAPI (blue) staining performed simultaneously in a single experiment for spleen sections from a HC and a pRD patient. Red arrow: T cell region, light blue arrow: GC light zone, dark blue arrow: GC dark zone. Magnification: 20x. **(b) PD-1 expression**. Magnification: 60x. **(c) PD-1 expression**. Magnification: 60x, yellow arrow depicts Tfh cells (CD4^+^PD-1^+^). **(d) Gating for naive, CM, EM TEMRA T**_**H**_ **cell subsets. (e) Enumeration of T cell subsets**. Data were obtained from HC-N (n = 1), HC-Ags (n = 10), pRD-N (n = 1) and pRD-Ags (n = 8) subjects. **(f) Productive Simpson clonality of cTfh TCR-Vβ repertoires. (g) CDR3 length distribution in cTfh TCR-Vβ repertoires**. Means of HCs and pRD patients are depicted as a black and red line, respectively. Individual values are shown for the patients. nt – nucleotides. **(h) CDR3 length of cTfh TCR-Vβ repertoires. (i) Differential usage of V genes in cTfh TCR-Vβ repertoires**. Relative frequency of TCR-Vβ family usages is shown. **(j) Productive Simpson clonality of Treg TCR-Vβ repertoires. (k) CDR3 length distribution in Treg TCR-Vβ repertoires**. Means of HCs and pRD patients are depicted as a black and red line, respectively. Individual values are shown for the patients. nt – nucleotides. **(l) CDR3 length of Treg TCR-Vβ repertoires. (m) Differential usage of V genes in Treg TCR-Vβ repertoires**. Relative frequency of TCR-Vβ family usages is shown. All TCR-Vβ repertoire data were obtained on HC-Ag (n = 7) and pRD-Ag (n = 6) subjects. **(n) Gating strategy for sorted CD38**^**int**^ **B and cTfh cells**. Gating and sort purity of batch sorted CD38^int^ B cells and cTfh cells for co-culture assay shown on a representative HC-Ag subject. Four independent experiments were performed with one HC-Ag (n = 4) and one pRD-Ag (n = 4) subjects in each. Data for e-f h-j and l-m are shown as mean±s.e.m. with individual values. Statistical analysis was performed using two-sided unpaired Student’s *t-*test for panel **f-h** and **j-l**, or Mann–Whitney *U* test with Holm-Šídák multiple comparison for panel **e, i** and **m**. Source data

Like B cells, T cells are also dysregulated in pRD and are characterized by decreased count, skewed toward activated memory cells and, specifically, suppressor capacity of regulatory T (T_reg_) cells is defective^4,38^., We confirmed decreased T_reg_ cell frequency and naive-memory ratio; and found remarkable expansion of circulating T_FH_ (cT_FH_) cells in our pRD cohort (Fig. 8a,b). Notably, all these T helper (T_H_) cell subsets were still normal in the asymptomatic patient with pRD-N. Of note, PD-1^+^ T_H_ cells were abundant in the spleen of a patient with pRD and uniformly distributed compared to an HC (Extended Data Fig. 10a–c). Assessment of naive, central memory, effector memory (EM) and terminally differentiated effector memory T (TEMRA) cells confirmed a significant decrease in the proportion of naive cells and revealed the strong expansion of EM T_H_ cells in pRD (Extended Data Fig. 10d,e). Of note, the proportion of PD-1^hi^ICOS^hi^ cells was elevated in each of the investigated T_H_ subsets of the patients (Fig. 8c,d) and the cT_FH_ cells displayed an activated phenotype (Fig. 8c,d). This was further confirmed by the lower expression level of quiescence marker, CCR7, in the PD-1^+^ fraction of the cT_FH_ (and memory) cells in pRD (Fig. 8e,f). These phenotypic alterations of pRD cT_FH_ cells were associated with reduced diversity of their TCR-Vβ repertoire (Extended Data Fig. 10f) with an elevated proportion of shorter CDR3 length (Extended Data Fig. 10g,h) when compared to HCs, whereas TCR-V_β_ gene usage was preserved (Extended Data Fig. 10i). Of note, the same alterations were observed in the TCR-V_β_ repertoire of the pRD T_reg_ cells (Extended Data Fig. 10j–m).

By assessing inflammatory and T_FH_ cell-specific cytokine-producing capacity of T_H_ cells in vitro we found elevated IFN-γ, TNF-α and IL-21 production in the patients (Fig. 8g,h). To determine whether increased cytokine production of total T_H_ cells merely results from the abundant presence of effector cells (cT_FH_ and memory) in pRD or whether these cells indeed possess elevated cytokine-producing capacity, we measured cytokine production on presorted T_H_ populations. Notably, IFN-γ and TNF-α production of effector T_H_ cells (cT_FH_ and memory) was comparable to those of HCs (Fig. 8i,j). Elevated IFN-γ and TNF-α production of naive T_H_ cells observed in pRD may reflect the inclusion of early effector-committed cells. In contrast, IL-21 production was found to be significantly elevated in the cT_FH_ and memory cells of patients with pRD, indicating their potential role in B cell activation (Fig. 8i,j). Indeed, by assessing resting mature naive B cell-activating capacity of sorted cT_FH_ cells (Extended Data Fig. 10n and Fig. 8k), we found that pRD cT_FH_ cells induced more robust B cell activation than those of HCs, as reflected by increased presence of plasma cells in the culture (Fig. 8l,m). Hence, pRD is associated with elevated frequencies of hyperactivated T_H_ and cT_FH_ cells that may promote the breach in B cell tolerance and the expansion of autoreactive B cells and may contribute the induction of T-bet^+^ B cells in pRD as demonstrated recently^39^.

## Discussion

Our central finding on B cells obtained from a large cohort of patients with pRD includes that (1) the formation of the primary immunoglobulin repertoire is severely restricted; (2) both the central and peripheral tolerance filters are impaired, allowing the accumulation of poly/autoreactive B cells in the primary immunoglobulin repertoire; and (3) nascent B cells undergo rapid activation, expansion and somatic diversification; and clonally related descendants persist and accumulate in the effector repertoires. Together, these events render the antibody repertoire of patients with pRD defective in their ability to efficiently clear foreign antigens but maintain reactivity to self.

First, we revealed severe diversity restriction with increased clonality in the primary repertoire and impaired somatic diversification in the effector compartment in pRD. A previous assessment of the repertoire of total B cells from peripheral blood of patients with pRD-CID demonstrated preserved diversity and proper somatic diversification^14^. The contradiction between the two studies is most likely based on the different approaches. Lee et al. performed analysis on total blood representing B cell subsets by their in vivo distribution, reflecting on mainly memory cells given their predominance over naive cells in pRD. Therefore, diversity, CSR and SHM could be seemingly normal, but features of the primary repertoire remains blurred. In contrast, by investigating B cell subsets separately, we documented restricted primary BCR repertoire with early CSR/SHM along with impaired somatic diversification in memory cells. These repertoire abnormalities accumulate in insufficient production of protective high affinity antibodies and inadequate humoral immune response in pRD. Indeed, impaired clearance of foreign antigens is demonstrated by persisting viral load, recurrent chronic infections^4^ and likely microbiota translocation due to intestinal inflammation and defective microbiota containment^40^. Hence, compromised adaptive immunity in pRD leads to the accumulation of foreign and/or microbiota-derived antigens, pathogen and damage-associated molecular pattern (PAMP/DAMP) molecules, exposing the immune system to persistent and abundant antigen load.

The second part of our findings delineated B cell tolerance impairments in pRD. Although, the role of *RAG1*/*RAG2* genes in receptor editing has been established^7,8^, our study quantitatively reports defective central tolerance in pRD. We also identified the peripheral tolerance impaired in all individuals with pRD investigated, implying an indirect role of the *RAG1*/*RAG2* genes in the process. Furthermore, patients with pRD-Ag accumulated more polyreactive B cells than the asymptomatic pRD-N infant, demonstrating the plasticity of peripheral tolerance and how it may worsen with age in pRD.

Regarding peripheral tolerance, plasma BAFF level seems to act as a major checkpoint, given that its excess rescues autoreactive B cells and promotes autoimmunity^41–43^. The high plasma BAFF level is likely resulted from B cell lymphopenia and inflammation in pRD. Progressive B cell lymphopenia in pRD is a direct consequence of impaired RAG function and may worsen with age^44^. In addition, BAFF expression further increases in the presence of type I IFNs, IFN-γ, IL-10 and granulocyte colony-stimulating factor (G-CSF) as well as by the activation of TLRs^45^. Therefore, chronic inflammation secondarily to impaired RAG function is likely an additional trigger to impair peripheral tolerance through BAFF. In addition, *RAG1*/*RAG2* genes play critical roles in the development^46^, repertoire and proper function of T_reg_ cells^38^, a key factor of peripheral B cell tolerance^37^. Hence, impaired T_reg_ cells also represent a defective peripheral B cell tolerance checkpoint in pRD. In addition, abnormal T_FH_ cell function may also contribute to the selection and affinity maturation of specific, high affinity class-switched autoantibody-producing B cells such as those targeting cytokines^5^. As pRD is associated with the expansion and overactivation of cT_FH_ cells it may contribute to the humoral autoimmunity beyond B cell-intrinsic components. These findings imply that targeted anti-BAFF, CTLA4-Ig and/or T_FH_ therapies may represent options for treatment of clinical autoimmune diseases in pRD.

The third component of our findings identified widespread promiscuous B cell activation that is likely the direct consequence of the two aforementioned phenomena. While in steady state, the population size of the antigen-specific B cells for any particular antigen is very small, a repertoire enriched for polyreactive clones allows for widespread antigen recognition and capture. Indeed, we have demonstrated that antibodies cloned from B cells (a surrogate of their BCRs) recognize a broad variety of antigens (such as DNA, protein and LPS) in pRD. Hence, nascent B cells expressing polyreactive BCRs are likely constantly exposed to PAMPs/DAMPs, which they can capture, internalize and deliver to TLRs. Co-signaling through BCRs and TLRs can synergistically trigger and sustain polyreactive B cell hyperactivation^47–49^. In addition, the inflammatory milieu secondary to impaired antigen clearance can also facilitate B cell activation by an antigen-independent manner via both the canonical and the non-canonical NF-κB pathways. In addition, widespread bystander T_H_ cell interactions may also serve as a potential driver, given that T_FH_ cells from patients had elevated capacity to induce B cell activation in vitro.

We propose that the combination of widespread B cell activation with progressive B cell lymphopenia creates a distinct immune phenotype in pRD. The premature activation of nascent B cells accelerates their development, rapidly bypassing early stages as indicated by the shrinking size of transitional and resting mature naive compartments, similarly as seen in a single case of a patient with pRD^50^ and also in severe SARS-CoV-2 infections^51^. Activation is also illustrated by the expansion of activated naive and other IgD^+^CD27^−^CD38^−^ naive subsets along with NSM compartments. These alterations seem to be a unique feature of pRD compared to other primary immune regulatory disorders and may serve as a biomarker for pRD. It also raises the question as to whether patients with pRD would benefit from monitoring and targeting persistent antigen load (such as virus infection and translocated microbiota) to predict and prevent widespread B cell activation that may escalate to clinical autoimmunity.

Another consequence of the widespread activation is reflected by the unusual early somatic diversification of the immunoglobulin loci, indicating exposure to antigens and/or cytokines that trigger AID activity^52,53^. SHM and CSR are the gold standards for distinguishing antigen-experienced B cells from naive cells and trump gating based on surface markers. Thus, although the CD38^int^ subset phenotypically represented resting mature naive B cells in pRD, at least a fraction of them cannot be considered bone fide naive cells. A similar phenomenon has been described in systemic lupus erythematosus, where altered chromatin accessibility and gene expression profile with AID induction were identified in phenotypically naive B cells^54,55^. This suggests that dysregulation of surface marker expression may occur frequently under pathological conditions and, therefore, does not accurately distinguish antigen-naive and antigen-experienced B cells.

Nevertheless, we found that widespread B cell activation is associated with a unique B cell fate and somatic diversification in pRD. Analyzing the trajectories of individual B cell clones in distinct repertoires of patients with pRD, we identified persisting polyreactive and non-polyreactive B cell clones with intercompartmental clonal connections, which undergo stochastic somatic diversification and acquire strong positive antigenic selection in selected cases. This may explain the presence of high affinity class-switched autoantibodies, such as those targeting IFN-α.

Activation of B cells with polyreactive BCRs recognizing highly repetitive motifs may drive these cells toward extrafollicular destiny. Indeed, CXCR5^lo^CD11c^hi^T-bet^+^ autoimmune-prone ABCs, with potential extrafollicular origin^34,56,57^, are expanded in pRD. These cells may stem from exaggerated TLR engagement due to the chronic exposure to pathogens from recurrent infections^33,57–59^. ABCs in mouse models or human diseases has been associated with humoral autoimmunity^33,56,58^, hence, shifting B cell maturation toward ABC fate provides an additional aspect of tolerance defect. In addition, dominance of potential extrafollicular B cell fate over the germinal center (GC) results in less efficient antibody affinity maturation and CSR rendering antigen clearance and microbiota containment impaired; and exaggerating immune dysregulation. Indeed, although the memory compartment is expanded, its impaired SHM and CSR indicates improper affinity maturation. Finally, activated memory B cells with increased HLA-DR expression and ABC fate may attain the ability to present antigens to naive T cells with high efficiency as professional antigen presenting cells in a TLR-dependent manner^57^. Hence, dysregulated B cells may contribute to T cell dysregulation and T_FH_ cell expansion in pRD.

In summary, our findings support the concept that adequate RAG function during early B cell development is critical to establish functional B cell tolerance and govern normal B cell development in the periphery. We demonstrated that impaired RAG function results in restricted primary B cell repertoire and impaired tolerance. Together these two events lead to inefficient humoral immunity with persistent antigenic load and a mature naive B cell pool enriched with polyreactive clones. On this ground, environmental triggers (such as chronic infections and microbiota translocation) along with intrinsic factors (such as elevated BAFF, reduced T_reg_/T_FH_ cell ratio and inflammatory cytokine milieu) gradually provoke profound B cell dysregulation with time that includes widespread B cell activation, developmental skewing, accumulation of T-bet^+^ cells and expansion of autoreactive clones. Therefore, our study demonstrates roles of *RAG1*/*RAG2* genes in regulating tolerance and governing B cell fate in the periphery that points beyond their well-understood role in V(D)J recombination and receptor editing.

## Methods

### Human samples

Sixteen patients with pRD and 27 healthy individuals were enrolled in this study. All were recruited according to protocols approved by local Institutional Review Boards (IRBs) as follows: USF-Pro00035468 (PI, J.E.W.), USF-Pro00025693 (PI, J.E.W.), JHMI-IRB00175372 (PI, J.E.W.), JHMI-IRB00097062 (PI, J.W.L.) and JHMI-IRB00097938 (PI, J.W.L.). Inclusion criteria for patients with pRD were carrying confirmed hypomorphic homozygous or compound heterozygous *RAG1*/*RAG2* mutations and displaying CID or CID-G/AI phenotype. Exclusion criteria for HCs were symptoms of cold, temperature above 37.8 °C and being treated with antibiotics or antivirals in the past 2 weeks. All protocols followed local ethics recommendations and informed consent was obtained. No compensation was provided. Peripheral blood samples from individuals with pRD and HCs were obtained via standard phlebotomy using Na-Hep or EDTA blood collection tubes and processed or frozen within 48 h. Paraffin-embedded spleen tissue blocks from P5 and an aged-matched HC were provided by B.N. and H.L.M., respectively.

### Autoantibody, anticytokine antibody and unmutated VH4-34 (9G4) antibody detection

Autoantibodies to protein antigens from plasma samples were detected with standard in-house developed ELISAs. Briefly, 96- or 384-well ELISA plates (MaxiSorp; Thermo Fisher Scientific) were coated with 1 or 2 μg ml^−1^ recombinant human proteins or 10 μg ml^−1^ human embryonic kidney 293 cell (HEK293) protein extract representing broad cellular autoantigen mixture in PBS by incubation overnight at 4 °C. Recombinant antigens were purchased from Surmodics (human M2 antigen, intrinsic factor, proliferating cell nuclear antigen, threonyl-tRNA synthetase, Ro/SSA-52 Kda, thyroid peroxidase, U1-SnRNP C protein, thyroglobulin and ribosomal phosphoprotein P0), BioLegend (IFN-α) or Thermo Fisher Scientific (IL-12 and IFN-ω). Human recombinant tissue transglutaminase protein was kindly gifted by L. Fesus (University of Debrecen). To detect anti-DNA antibodies, plates were coated with 10 μg ml^−1^ DNA (Sigma-Aldrich) in PBS. Plates were then washed in PBS-Tween 0.05%, blocked by incubation with the same buffer supplemented with 2% BSA, washed and then incubated with 1:100, 1:200, 1:400 and 1:800 dilutions of plasma samples from patients or controls for 2 h at room temperature. Plates were thoroughly washed and then incubated with HRP-conjugated Fc-specific polyclonal goat anti-human IgG Ab (Sigma-Aldrich) for 1 h at room temperature. After washing, HRP substrate was added (TMB; Thermo Fisher Scientific), the reaction was terminated with sulfuric acid (Sigma-Aldrich) and absorbance was measured at 450 nm in an ELISA reader. Results are expressed as the blank corrected absorbances and *z* scores (corrected absorbance minus mean of HCs divided by s.d. of the HC values). Unmutated VH4-34 (9G4) IgM antibody was detected by coating the ELISA plate with 9G4-purified rat anti-idiotypic antibody (IGM Biosciences) and using HRP-conjugated Fc-specific polyclonal goat anti-human IgM antibody (Thermo Fisher Scientific).

### Cytokine, endotoxin, soluble IL-2 receptor and virus (EBV and CMV) quantification from plasma

IFN-γ, IL-10, BAFF (R&D Systems) and endotoxin (Lifespan Bioscience) plasma concentrations were measured using ELISAs. sIL-2R plasma level was measured by Luminex assay (Thermo Fisher Scientific). EBV and CMV virus titers were determined by Viracor Eurofins.

### Cell preparation, flow cytometry and cell sorting

Peripheral blood mononuclear cells were isolated from peripheral blood samples by using Ficoll-Paque PLUS density gradient centrifugation (GE Healthcare Life Sciences). B cells were enriched with positive magnetic separation using anti-CD20 magnetic beads (Miltenyi Biotec). Cells were stained with live–dead discriminator (Fixable Viability Dye eFluor 780, Thermo Fisher Scientific), Fc receptors were blocked using Human TruStain FcX (BioLegend) and then surface antibody stained. When appropriate, surface-stained cells were fixed, permeabilized and followed by intracellular staining. Cells were analyzed and/or sorted with a FACSAria II (BD Bioscience) using FACSDiva software v.10. Fluorescence-activated cell sorting data were visualized with FlowJo software v.10.7.1 (TreeStar) and, when indicated, further analyzed by FlowSOM (v.2.5), DownSample (v.3.0), *t*-SNE (v.2.0) and HyperFinder (v.0.6.2) FlowJo plugins.

### Unsupervised high-dimensional clustering for B cell subset identification and quantification

Unsupervised high-dimensional analysis was performed using FlowSOM plugin^60^ to achieve objective B cell subset identification and quantification from the patients with pRD. Briefly, flow cytometry data obtained from four patients (P9, P12, P14 and P16) and four age-appropriate HCs (C1, C3, C5 and C7) (inclusion criteria of data were having at least 5,000 live CD19^+^ cells and acquisition performed with identical flow cytometry panel) for CD21, CD24, CD27, CD38, IgD and IgM parameters were simultaneously used. First, full datasets were randomly downsampled to 5,000 live CD19^+^ cells from each donor using DownSample FlowJo plugin. Next donors were concatenated and then each cell of the generated B cell pool was assigned by a self-organizing map in a 10 × 10 grid, resulting 100 different nodes. Then, minimal spanning trees (MSPs) were built from the nodes in each donor to visualize similar nodes in branches and their relative connection to each other (Extended Data Fig. 3a). To identify B cell populations, nodes were classified into metaclusters that revealed 16 different metaclusters each representing a unique B cell subset (Extended Data Fig. 3b). To associate these metaclusters to B cell subsets, first, the mean values of each marker (Extended Data Fig. 3c) were min–max normalized in each metacluster for all donors to rescale value range of 0–1. Next, the means of each value were calculated and value ranges for the expression of each marker were defined as 0–0.1, 0.1–0.3, 0.3–0.7, 0.7–0.9 and 0.9–1.0 corresponding to ‘negative’, ‘low’, ‘intermediate’, ‘positive’ and ‘high’ expression levels, respectively (Extended Data Fig. 3d). In the last step, each metacluster was assigned to a unique B cell subset based on the expression pattern of the six markers and the current advances of human B cell immunophenotyping^28^. Relative frequencies of each subset by donors were also obtained from the FlowSOM analysis (Extended Data Fig. 3e).

### *t*-distributed stochastic neighbor embedding

A *t*-SNE algorithm was used for visualizing the multidimensional flow dataset in a dimension-reduced data space to compare B cell subset distribution in pRD compared to HCs. *t*-SNE analysis was performed on the concatenated dataset defined in the FlowSOM analysis with the same parameters (CD21, CD24, CD27, CD38, IgD and IgM) using default settings.

### Establishing two-dimensional sequential gating strategy for B cell subset identification using HyperFinder

To extend immunophenotyping to the entire patient cohort a two-dimensional sequential gating was constructed using HyperFinder FlowJo plugin. Briefly, HyperFinder was applied on the concatenated dataset defined in the FlowSOM analysis with the same parameters (CD21, CD24, CD27, CD38, IgD and IgM) for searching all combinations of available parameters to find a two-dimensional convex polygon gating strategy that most accurately distinguishes the target B cell subset from the background B cell populations. Optimal sequential gatings for each of the 16 target B cell subsets were automatically calculated by maximizing Fm-score. This approach accurately recapitulated FlowSOM analysis on the test dataset (P9, P12, P14, P16, C1, C3, C5 and C7) as demonstrated with high F-measures for each B cell subset (0.84 ± 0.06 mean ± s.d., range 0.68–0.96). Therefore, the constructed sequential gatings were utilized on the patients with pRD (14 pRD-Ag and one pRD-N) along with 20 HCs (16 HC-Ag and 4 HC-N) to evaluate their immunophenotype (Extended Data Fig. 3f).

### Immunohistochemistry

Immunohistochemistry was performed using anti-CD20 (L26 mouse monoclonal primary antibody, Roche Diagnostics, 0.3 μg ml^−1^), anti-CD21 (2G9 mouse monoclonal primary antibody, Cell Marque Tissue Diagnostics, 0.5 μg ml^−1^), anti-CD4 (SP35 rabbit monoclonal primary antibody, Roche Diagnostics, 2.5 μg ml^−1^) and anti-BCL2 (I24 mouse monoclonal primary antibody, Roche Diagnostics, 2.62 μg ml^−1^) antibodies on a Leica DM LB2 clinical brightfield microscope.

### Multicolor immunofluorescence staining

Human spleen samples were fixed in formalin, embedded in paraffin and sectioned at 5 μm. After deparaffinization, antigen retrieval was performed to unmask the antigenic epitope with 1× Tris-EDTA, pH 8.5, buffer (Sigma-Aldrich E1161) in a decloaking chamber (Biocare Medical). Sections were blocked for 1 h at room temperature with TBS (0.025% Triton X-100, 1% BSA and 0.3 M glycine), then incubated with the following monoclonal antibodies at 1:100 dilution in TBS (0.025%Triton X-100 and 1% BSA) overnight at 4 °C: anti-CD19 antibody coupled to AlexaFluor488 (ab196468, Abcam); anti-CD4 antibody coupled to AlexaFluor555 (ab280849, Abcam); anti-T-bet antibody coupled to AlexaFluor647 (ab225198, Abcam); or anti-PD-1 antibody coupled to AlexaFluor647 (ab201825, Abcam). The samples were then mounted with Fluoromount-G (0100-01 Southern Biotech) and imaged under a confocal microscope with a resonant scanning disk (Nikon A1R, Nikon Instruments) with Z-sectioning (0.5 μm), using Nikon NIS-Elements AR Analysis 4.40 software.

### Indirect fluorescent antibody assay

For the detection of autoantibodies against cellular antigens, we performed IFA on HEp-2 slides (human epithelial cells, Bio-Rad) following the manufacturer’s instructions. Briefly, plasma samples (25 μl) were added to the substrate for 20 min. Then, substrates were rinsed with PBS for 10 min and incubated with 25 μl fluorescein-conjugated (FITC) antiserum for 20 min at room temperature. Slides were then washed with PBS and counterstained with Evans blue dye in PBS for 10 min first and with DAPI for additional 10 min. The slides were finally washed in PBS, drained and mounted with coverslip. For optimal visualization of the topographic distribution of the immunofluorescence (pattern) and quantification (titer) of the autoantibodies present in the plasma samples, confocal microscopy was performed. Nuclear binding of autoantibodies was determined from three-dimensional reconstructed confocal images as a fraction of the sum of fluorescence intensity contained in the DAPI-positive area by total DAPI-positive area per field. Cytoplasmic binding was quantified as the fraction of the sum of fluorescence intensity in Evans blue-positive area by total Evans blue-positive area per field. Eight high-resolution confocal images per sample (×60 lenses; 1,024 × 1,024 pixels; Nikon A1R; Nikon Instruments) with thin Z-sectioning (0.1 μm) were captured and analyzed with Nikon NIS-Elements AR Analysis 5.21 software. Evaluation of the staining pattern (nucleus, homogenous, rim, speckled, nucleolar and spindle; cytoplasm, mitochondrial and smooth muscle) and fluorescent intensity grade (+4, brilliant apple green; +3, bright apple green; +2, clearly distinguishable positive; +1, lowest specific distinguishable from background and 0, negative) was performed according to the manufacturer’s instructions.

### In vitro cell cultures

For the naive B cell differentiation assay, 5 × 10^4^ sorted resting naive B cells (CD19^+^CD27^−^IgD^+^CD24^int^CD38^int^IgM^+^) (purity >98%) were seeded in a 96-well culture plate in lymphocyte medium (RPMI 1640 complete medium (100 μl per well) supplemented with 10% heat-inactivated fetal bovine serum and activated with CpG ODN 2006 (400 μg ml^−1^; InvivoGen), recombinant human CD40L (1 μg ml^−1^; R&D Systems) and recombinant human IL-21 (50 ng ml^−1^; BioLegend). After 7 d cells were stained and analyzed for memory B cells (live CD19^+^CD27^+^CD38^lo-int^) and plasma cells (live CD19^+^CD27^+^CD38^hi^) by flow cytometry.

For activation of naive B cells with human plasma, 5 × 10^4^ sorted resting naive B cells (CD19^+^CD27^−^IgD^+^CD24^int^CD38^int^IgM^+^) (purity >98%) from an HC were cultured in vitro in 96-well culture plate in 100 μl per well complete RPMI 1640 or ImmunoCult Human B Cell Expansion medium (StemCell) supplemented with recombinant human IL-21 (10 ng ml^−1^; BioLegend) and either plasma from a patient with pRD or independent HC plasma (1.25, 2.5, 5, 10 and 20%). Cells were analyzed on day 2 for surface expression of activation markers (CD25 and CD86) by flow cytometry. For the detection of IFN-γ, TNF-α and IL-21 produced by T_H_ cells, frozen peripheral blood mononuclear cells were thawed and 10^6^ cells per well were plated in a 12-well culture plate and then rested overnight at 37 °C in a CO_2_ incubator. Cells were stimulated with phorbol 12-myristate 13-acetate (PMA) and ionomycin in the presence of brefeldin A and monensin (eBioscience Cell Stimulation Cocktail 500×; Thermo Fisher Scientific) for 4 h then surface stained, followed by intracellular staining for cytokines and detected by flow cytometry.

For cT_FH_ and naive B cell co-culture, 5 × 104 sorted resting naive B cells (CD19^+^CD27^−^IgD^+^CD24^int^CD38−IgM^+^) (purity >98%) from an HC were co-cultured with 2.5 × 104 cT_FH_ cells sorted either from an independent HC or patient with pRD in a round-bottom 96-well culture plate in complete RPMI 1640 medium (100 μl per well) supplemented with 10% heat-inactivated fetal bovine serum. The cells were mixed and cultured in the presence of endotoxin-reduced staphylococcal enterotoxin B (SEB) at 1 μg ml^−1^ for 7 d then plasma cells (live CD19^+^CD27^+^CD38^hi^) were detected among total B cells by flow cytometry.

### Monoclonal antibody cloning

NE transitional (CD27^−^IgD^+^CD38^hi^CD24^hi^IgM^hi^CD21^lo^CD10^+^) and resting mature naive (CD27^−^IgD^+^CD38^int^CD24^int^IgM^+^CD21^+^CD10^−^) B cells were single sorted into dry 96-well PCR plates. RNA from single B cells was reverse-transcribed in the original 96-well plate in 10.5-μl reactions containing 150 ng random hexamer primer (pd(N)6, Amersham Pharmacia Biotech), 0.5 μl dNTP-Mix (10 mM each nucleotide), 1 μl 0.1 M dithiothreitol, 0.5% v/v NP40, RNase inhibitors (0.8 U RNAsin, Promega) and 40 U Superscript II reverse transcriptase (Invitrogen) at 42 °C for 55 min. Immunoglobulin heavy-chain, immunoglobulin λ-chain or immunoglobulin κ-chain transcripts were amplified by two rounds of PCR, restriction sites were introduced by the second PCR. PCR reactions contained 0.25 μM primers, 1 U Hotstar Taq DNA polymerase (QIAGEN) and 3.5 μl complementary DNA for first PCR in 20 μl or 3.5 μl first PCR product for second PCR in 25-μl reactions. Each round of PCR was performed for 50 cycles at 94 °C for 30 s, 57 °C (heavy chain/κ-chain) or 60 °C (λ-chain) for 30 s, 72 °C for 55 s (first PCR) or 45 s (second PCR). Primer sequences are listed in Supplementary Table 5. All PCR products were purified, sequenced and analyzed by IMGT/V-QUEST as described at BCR repertoire analysis. Products were directly cloned into expression vectors containing human IgG1, κ-chain or λ-chain constant regions. Plasmids were sequenced to select clones with inserts identical to the original PCR product and used for monoclonal antibody production.

### Monoclonal antibody production and purification

The 293A HEK fibroblasts were cultured in DMEM supplemented with 10% ultra-low IgG FCS (GIBCO) and co-transfected with 5 μg of heavy- and light-chain-encoding plasmid DNA using polyethylenimine (PEI). Between 8–12 h after transfection, cells were washed with serum-free DMEM and cultured in DMEM supplemented with 1% Nutridoma SP (Roche). Supernatants were collected after 10 d of culture. For self-reactivity HEp-2 IFAs, antibodies were purified on protein G Sepharose (Amersham Pharmacia Biosciences).

### Poly- and self-reactivity tests

Recombinant antibodies were tested with ELISA at concentrations of 1, 0.25, 0.0625 and 0.0156 µg ml^−1^ for anti-dsDNA, insulin and LPS reactivity with two positive controls used in each assay to determine threshold for positive reactivity (defined as mean of positive controls minus 2 s.d. at 1 µg ml^−1^ antibody concentration). Antibodies were considered polyreactive when they recognized at least two of the three antigens tested. Recombinant antibodies were also tested for anti-HEp-2 cell line lysate reactivity with ELISA at a concentration of 5 µg ml^−1^. Blank corrected absorbance values were normalized with the mean of positive controls minus 2 s.d. and values above 1 were considered positive for HEp-2 reactivity. For indirect immunofluorescence, assay recombinant antibodies were incubated in 200 µg ml^−1^ concentration in HEp-2 cell-coated slides (MBL International) and detected according to the manufacturer’s instructions.

### BCR repertoire analysis

For preparation of the input heavy-chain sequences, bulk, pre-annotated heavy- and light-chain sequence data were generated by iRepertoire from the messenger RNA extracts of sorted B cells (25,000–50,000 cells per sample). To be consistent throughout our studies, we extracted all raw nucleotide sequences from these datasets and reannotated using IMGT/HighV-QUEST (program v.3.4.17, reference directory release, 201915-3)^61^. Unless specified otherwise, all further data processing steps were performed using algorithms of the Immcantation framework^62^, Tidyverse system (H. Wickham and G. Grolemund, R for Data Science) and the R language (v.3.6.3) (Extended Data Fig. 6a). IMGT annotated data were first converted to a standard adaptive immune receptor repertoire (AIRR) rearrangement format with ‘MakeDb.py’ script of Change-O package (v.1.0.0)^63^. Copy numbers and isotypes were brought back to these repertoire datasets from the original iRepertoire annotations. For subsequent steps, only the CDR1-FWR4 region was kept everywhere. Separate records of identical sequences were collapsed into one unique record for each sequence summing their copy numbers. To reduce the potential bias originating from the errors of reverse transcription, amplification and sequencing, we utilized the following strategy: sequences with fewer than five copies were pulled out into a separate, low-copy pool retaining only a high-copy subset. Each low-copy sequence was matched to every high-copy sequence. If the Hamming distance between a low-copy sequence and a high-copy counterpart was not higher than one, then the low-copy sequence became merged into the high-copy pool by adding its copy number to the closest high-copy sequence. All other unmatched, low-copy sequences were discarded. Finally, all non-functional sequences were removed from the merged pool. Single-clone heavy-chain sequences of previously in vitro characterized, functional monoclonal antibodies of the individuals were annotated with IMGT in the same way as iRepertoire data.

For the treemap, to visualize diversity, we plotted the relative frequencies of the most abundant 2,500 clones on treemaps categorized by 58 V_H_ gene families. Divergent color hues were assigned to different V_H_ genes unified across all repertoires. Distinct clones sharing the same V_H_ gene were displayed using altered brightness of the same hue. Treemaps were plotted with the ‘treemap’ function from treemap R package^64^.

For gene usage, V, D and J gene usages were quantified by calling the ‘countGenes’ R function (Alakazam v.1.0.2 package) on repertoires.

For the SHM profile, SHM levels were measured with the ‘observedMutations’ function (SHazaM v.1.0.2) for each sequence and the mean of these values was used. The 5-mer nucleotide motif mutability probability values or SHM-targeting model in each compartment was calculated by ‘createTargetingModel’ function and visualized as linear or circular (‘hedgehog’) bar graphs using the ‘plotMutability’ function (SHazaM v.1.1.0). Descriptive indices (D50, Shannon–Wiener and Gini–Simpson) were calculated implementing standard statistical formulas in R.

For Bayesian estimation of antigen-driven selection (BASELINe), an established method was followed to quantify antigen selection^65–67^. In each sequence dataset (repertoires, random downsampled subsets or clonotype lineages), first the posterior probability density function for every single sequence was calculated by using ‘calcBaseline’. Mean selection strength (mean Sigma), the 95% confidence intervals and *P* values were determined with ‘summarizeBaseline’ function (both from SHazaM v.1.0.2 package). Sigma values were color-coded using a blue–gray–red palette (negative–zero–positive) to demonstrate selection strength of single sequences on corresponding figures.

For unsupervised clonal clustering of heavy-chain sequences, initially, we attempted to apply the ‘findThreshold’ method (SHazaM v.1.0.2 package) as a part of the automatic grouping of closely related clonotype sequences but it failed in a proportion of the patients with pRD. Hence, we developed an algorithm for this task to keep the main concept of detecting and working with a distance threshold similar to the mentioned procedure. Briefly, Hamming distances of nearest neighbors of each heavy-chain sequences were calculated by the ‘distToNearest’ R function (SHazaM v.1.0.2 package) using sequence length normalization (mode, ‘ham’; normalize, ‘len’; and first, ‘FALSE’). The histogram representing these distances in the repertoire was smoothed and after the first peak, the position of the first non-declining value instead of the previously used local minimum was determined. Subsequently, this position as the distance threshold was used to generate clonotype clusters utilizing the ‘DefineClones.py’ script (–mode gene–act set–norm len–model ham). Putative closest germline sequences were added to the prepared and clonally grouped data by ‘CreateGermlines.py’ algorithm (-g dmask–cloned).

For identification of the relatives of cloned heavy-chain sequences (ImmChainTracer pipeline), we assembled a pipeline (ImmChainTracer) that collects close relatives of in vitro characterized and IMGT annotated, single-clone, naive B cell-derived heavy-chain sequences in specified repertoires. The Hamming distance threshold was determined on the full target repertoire for subsequent clonotype clustering. V, J gene and junction length properties of the input single-cell DNA sequence were applied as filter criteria to exclude the majority of non-relevant sequences and to reduce the size of the target repertoire substantially making a pre-filtered ‘mini repertoire’. Input DNA sequence data in question were added to this reduced AIRR dataset. Clonal clustering in this combined, pre-filtered AIRR dataset grouped all related sequences around the input sequence. All sorted members of the queried B cell lineage were revealed by the extraction and merging of such groups (clonotypes) of sequences from all compartments of the individual.

For clonal connectivity within compartments (lineage network diagrams), phylogenesis of related B cells inside clonotype clusters was inferred by the DNA parsimony approach in ‘dnapars’ program (PHYLIP v.3.697 software package J. Felsenstein, ‘PHYLIP, Phylogeny Inference Package’ (v.3.2)) embedded in the Alakazam module (v.1.0.2 package) and represented as graphs or trees using the igraph (v.1.2.5) R library (G. Csardi and T. Nepusz). Network diagrams were plotted as a multilineage graph set consisting of 2,500 unique, randomly selected, clonally grouped sequences, where nodes represent distinct havy-chain sequences (single clones) and edges bind only related sequences (clones). Size of the nodes refer to the abundance of corresponding clones on a logarithmic scale. Different coloring schemes of the nodes were used to demonstrate compartment affiliation, mutation burden, isotype or antigen selection strength.

For clonal connectivity among compartments (circular visualization plots), first, CD38^int^, CD38^−^ and CD27^+^ (or NE in case of infant donors) repertoire data were randomly downsampled to 10,000 sequences. Abundance of each sequence and clonal connections between related clones in different compartments were visualized using circular visualization plots using an already published algorithm^29^.

### TCR repertoire analysis

Annotated TCR-Vβ sequence data were generated by Adaptive Biotechnologies from the DNA extracts of 25,000-50,000 sorted Treg and cTfh cells. Data for graphical representation of TCR-V_β_ gene rearrangements, CDR3 length and Simpson clonality index were generated and extracted using the Adaptive Immunoseq Analyzer 3.0 software. All fasta data files generated for the analyses are available in the NCBI submission portal under accession code PRJNA746291.

### Statistical analysis

Statistical analyses were performed using GraphPad Prism (v.9) or R software. Statistical analyses were two-sided. For two groups, statistical analyses were performed using unpaired Student’s *t*-tests. A Mann–Whitney *U*-test was used when data did not follow normal distribution according to the Shapiro–Wilk test. For more than two groups, statistical analyses were performed using a Mann–Whitney *U*-test with multiple comparison (Holm–Šídák method). In all graphs each dot represents one individual and results are shown as the mean ± s.e.m. Correlations between two variables were assessed using nonparametric Spearman correlation tests. *P* values were considered significant at *P* < 0.05 and are shown on each graph when reaching statistical significance between groups. Each assay was carried out with as many patients as were available. No statistical methods were used to pre-determine sample sizes.

### Reporting summary

Further information on research design is available in the Nature Research Reporting Summary linked to this article.

## Online content

Any methods, additional references, Nature Research reporting summaries, source data, extended data, supplementary information, acknowledgements, peer review information; details of author contributions and competing interests; and statements of data and code availability are available at 10.1038/s41590-022-01271-6.

## Supplementary information

Supplementary Information Supplementary Tables 1–5.

Reporting Summary

## Data Availability

BCR repertoire sequencing data that support the findings of this study have been deposited in the NCBI submission portal under accession code (PRJNA746291). iRepertoire pre-processed data files (iRepertoire_preprocessed.zip), data files further processed by our analysis pipeline (prepared_AIRR_HC.zip and prepared_AIRR_pRD.zip) and the individual heavy-chain variable sequences cloned from single B cells (prepared_AIRR_single_clone_IgH_sequences.zip) are available on GitHub (https://github.com/blazsop/pRD-data). All additional data needed to evaluate the conclusions in this study are present in the main text or Supplementary Information. TCR repertoire sequencing data are available under accession code PRJNA746291. Source data are provided with this paper.

## Source data

Source Data Fig. 1 Statistical Source Data.
Source Data Fig. 2 Statistical Source Data.
Source Data Fig. 2 Image Source Data.
Source Data Fig. 3 Statistical Source Data.
Source Data Fig. 4 Statistical Source Data.
Source Data Fig. 4 Image Source Data.
Source Data Fig. 5 Statistical Source Data.
Source Data Fig. 6 Statistical Source Data.
Source Data Fig. 7 Statistical Source Data.
Source Data Fig. 8 Statistical Source Data.
Source Data Extended Data Fig. 1 Statistical Source Data.
Source Data Extended Data Fig. 2 Statistical Source Data.
Source Data Extended Data Fig. 2 Image Source Data.
Source Data Extended Data Fig. 3 Statistical Source Data.
Source Data Extended Data Fig. 4 Statistical Source Data.
Source Data Extended Data Fig. 5 Image Source Data.
Source Data Extended Data Fig. 7 Statistical Source Data.
Source Data Extended Data Fig. 8 Statistical Source Data.
Source Data Extended Data Fig. 10 Statistical Source Data.

## Code availability

Custom analysis scripts are available on GitHub (https://github.com/blazsop/airrmine/).
