# Supplementary material for: Partial RAG deficiency in humans induces dysregulated peripheral lymphocyte development and humoral tolerance defect with accumulation of T-bet+ B cells
Source: Nat Immunol. 2022 Jul 28;23(8):1256–72. doi: 10.1038/s41590-022-01271-6 (PMC9355881; doi:10.1038/s41590-022-01271-6)
Supplement: Supplementary file 2 — Reporting Summary [file 41590_2022_1271_MOESM2_ESM.pdf]

nature portfolio

Corresponding author(s): Jolan E. Walter, Krisztian Csomos

Last updated by author(s): Jun 13, 2022

Reporting Summary

Nature Portfolio wishes to improve the reproducibility of the work that we publish. This form provides structure for consistency and transparency in reporting. For further information on Nature Portfolio policies, see our Editorial Policies and the Editorial Policy Checklist.

Statistics

For all statistical analyses, confirm that the following items are present in the figure legend, table legend, main text, or Methods section.

n/a	Confirmed	
☐	☒	The exact sample size (*n*) for each experimental group/condition, given as a discrete number and unit of measurement
☐	☒	A statement on whether measurements were taken from distinct samples or whether the same sample was measured repeatedly
☐	☒	The statistical test(s) used AND whether they are one- or two-sided<br>*Only common tests should be described solely by name; describe more complex techniques in the Methods section.*
☐	☒	A description of all covariates tested
☐	☒	A description of any assumptions or corrections, such as tests of normality and adjustment for multiple comparisons
☐	☒	A full description of the statistical parameters including central tendency (e.g. means) or other basic estimates (e.g. regression coefficient) AND variation (e.g. standard deviation) or associated estimates of uncertainty (e.g. confidence intervals)
☐	☒	For null hypothesis testing, the test statistic (e.g. *F*, *t*, *r*) with confidence intervals, effect sizes, degrees of freedom and *P* value noted<br>*Give P values as exact values whenever suitable.*
☒	☐	For Bayesian analysis, information on the choice of priors and Markov chain Monte Carlo settings
☒	☐	For hierarchical and complex designs, identification of the appropriate level for tests and full reporting of outcomes
☐	☒	Estimates of effect sizes (e.g. Cohen's *d*, Pearson's *r*), indicating how they were calculated

Our web collection on statistics for biologists contains articles on many of the points above.

Software and code

Policy information about availability of computer code

Data collection	Flow cytometry data were collected on a FACSAria II (BD Biosciences) with FACSDiva software version 10. Immunohistochemistry was performed on a Leica DM LB2 clinical brightfield microscope (Leica Microsystems Inc.) using Leica Application Suite V4.9 software. Confocal microscopy images were performed on Nikon NIS-Elements AR Analysis 4.40 software.<br>ELISA plates were read on an EnSpire Workstation (PerkinElmer) (EnSpire software version 4.13.3005.1482)<br>B cell receptor (BCR) sequencing data was generated, preprocessed, and provided by iRepertoire.<br>Custom analysis scripts are available on GitHub (https://github.com/blazsop/airrmine/).
Data analysis	The analysis of flow cytometry data was performed using FlowJo software (version 10.7.1) and FlowJo plugins including FlowSOM (v2.5), tSNE (v2.0), Hyperfinder (v0.6.2) and DownSample (v3.0). Data representation and statistical analysis was performed using Graphpad Prism (version 9.1.0). BCR repertoire analysis was performed using IMGT/HighV-QUEST (version: 3.4.17, reference directory release: 201915-3) and R (version 4.0.4, 2021-02-15) on a x86_64-pc-linux-gnu (64-bit) platform. T cell repertoire data was analyzed with Adaptive Immunoseq Analyzer 3.0 software.

For manuscripts utilizing custom algorithms or software that are central to the research but not yet described in published literature, software must be made available to editors and reviewers. We strongly encourage code deposition in a community repository (e.g. GitHub). See the Nature Portfolio guidelines for submitting code & software for further information.

Data

Policy information about availability of data

All manuscripts must include a data availability statement. This statement should provide the following information, where applicable:

- Accession codes, unique identifiers, or web links for publicly available datasets
- A description of any restrictions on data availability
- For clinical datasets or third party data, please ensure that the statement adheres to our policy

BCR and TCR repertoire sequencing data that support the findings of this study have been deposited in the NCBI submission portal under accession code PRJNA746291. iRepertoire pre-processed data files (iRepertoire_preprocessed.zip), data files further processed by our analysis pipeline (prepared_AIRR_HC.zip and prepared_AIRR_pRD.zip) and the individual IgH variable sequences cloned from single B cells (prepared_AIRR_single_clone_IgH_sequences.zip) are available on GitHub (https://github.com/blazsop/pRD-data). All additional data needed to evaluate the conclusions in this study are present in the main text or Supplementary Information. Pathogenicity of RAG mutations was assessed based on data obtained from ClinVar database: https://www.ncbi.nlm.nih.gov/clinvar/

Field-specific reporting

Please select the one below that is the best fit for your research. If you are not sure, read the appropriate sections before making your selection.

☒ Life sciences ☐ Behavioural & social sciences ☐ Ecological, evolutionary & environmental sciences

For a reference copy of the document with all sections, see nature.com/documents/nr-reporting-summary-flat.pdf

Life sciences study design

All studies must disclose on these points even when the disclosure is negative.

Sample size

This study is purely explorative performed on a cohort of patients carrying hypomorphic pathogenic RAG1 or 2 gene variants and presenting with combined immune deficiency with granuloma / autoimmunity phenotype. This is a rare disease with an estimated frequency of 1:150,000 of the human general population. Therefore, no statistical methods were conducted for sample-size calculation prior to patient enrollment due to the nature of the study and patients. All designed experiments were performed on the patient lymphocyte or plasma samples depending on sample availability. In all experiments data were generated on sample sizes with at least 4 patients (and healthy control) samples per group, representing biological replicates.

Data exclusions

Flow cytometry data were excluded from the experiment and statistical analysis when cell abundance was less than 1,000 cells in target gate. Chronic clinical viremia with high viral load of EBV and CMV caused marked B cell proliferation in P13 not reflecting intrinsic B cell characteristic in this patient. Therefore, this single data point was excluded from data representation and analysis when we analyzed B cell number in peripheral blood of the pRD patients (Extended data Figure 4).

As infant subjects (both healthy and patient) display different immunophenotypic features and BCR repertoire characteristics compared to antigen experienced subjects, these data points were excluded from all statistical analysis presented in the study. However, for representation and comparison with antigen experienced subjects, data from infants were included in all figures with different symbols when available. All details are described in corresponding legends for each figure.

Sample exclusion: blood samples were not used if not processed within 48 hours of blood collection.

Replication

Biological replicates were used in the study for flow cytometry immunophenotyping, in vitro cultures and BCR repertoire analysis based on patient sample availabilities as detailed in each figure legend .

For the generation of recombinant antibodies, cloning was performed in duplicates from each sorted B cells. Only clones representing identical Ig heavy chain DNA sequences (and light chain sequences) were used for antibody expression and reactivity tests. All attempts for replication were successful.

ELISA assays were performed with duplicates and with serial dilutions of the plasma samples. All attempts for replication were successful.

In vitro functional assays on lymphocytes were performed with two or three replicates when applicable as indicated in corresponding figure legends. All attempts for replication were successful.

Replication of BCR repertoire sequencing from the same subject was not feasible in this study. Therefore, it was performed one time from each subject and data from different subjects represents biological replicates by study groups. P14 is an exception as BCR repertoire sequencing was performed at two different time points on this subject as indicated in legend of Figure 5.

Randomization

Randomization was not relevant to our study due to the nature of the disease and patient subjects. All available patients were included in the study and used for experiments if met the enrollment criteria described in Methods.
For all experiments, aged range-matched healthy donor samples were used in parallel with the patient samples to ensure accuracy of the assay and serve as data for healthy controls in the statistical analysis.
Exclusion criteria for healthy subjects are specified in Methods.

Blinding	No blinding was applied due to the nature of the study performed on rare patient subjects (only ex vivo experiments were performed. Researchers were involevd in obtaining patient blood samples, organizing and receiving sample shipments, performing and analyzing experiments, hence, blinding was not applicable)

Behavioural & social sciences study design

All studies must disclose on these points even when the disclosure is negative.

Study description	*Briefly describe the study type including whether data are quantitative, qualitative, or mixed-methods (e.g. qualitative cross-sectional, quantitative experimental, mixed-methods case study).*
Research sample	*State the research sample (e.g. Harvard university undergraduates, villagers in rural India) and provide relevant demographic information (e.g. age, sex) and indicate whether the sample is representative. Provide a rationale for the study sample chosen. For studies involving existing datasets, please describe the dataset and source.*
Sampling strategy	*Describe the sampling procedure (e.g. random, snowball, stratified, convenience). Describe the statistical methods that were used to predetermine sample size OR if no sample-size calculation was performed, describe how sample sizes were chosen and provide a rationale for why these sample sizes are sufficient. For qualitative data, please indicate whether data saturation was considered, and what criteria were used to decide that no further sampling was needed.*
Data collection	*Provide details about the data collection procedure, including the instruments or devices used to record the data (e.g. pen and paper, computer, eye tracker, video or audio equipment) whether anyone was present besides the participant(s) and the researcher, and whether the researcher was blind to experimental condition and/or the study hypothesis during data collection.*
Timing	*Indicate the start and stop dates of data collection. If there is a gap between collection periods, state the dates for each sample cohort.*
Data exclusions	*If no data were excluded from the analyses, state so OR if data were excluded, provide the exact number of exclusions and the rationale behind them, indicating whether exclusion criteria were pre-established.*
Non-participation	*State how many participants dropped out/declined participation and the reason(s) given OR provide response rate OR state that no participants dropped out/declined participation.*
Randomization	*If participants were not allocated into experimental groups, state so OR describe how participants were allocated to groups, and if allocation was not random, describe how covariates were controlled.*

Ecological, evolutionary & environmental sciences study design

All studies must disclose on these points even when the disclosure is negative.

Study description	*Briefly describe the study. For quantitative data include treatment factors and interactions, design structure (e.g. factorial, nested, hierarchical), nature and number of experimental units and replicates.*
Research sample	*Describe the research sample (e.g. a group of tagged Passer domesticus, all Stenocereus thurberi within Organ Pipe Cactus National Monument), and provide a rationale for the sample choice. When relevant, describe the organism taxa, source, sex, age range and any manipulations. State what population the sample is meant to represent when applicable. For studies involving existing datasets, describe the data and its source.*
Sampling strategy	*Note the sampling procedure. Describe the statistical methods that were used to predetermine sample size OR if no sample-size calculation was performed, describe how sample sizes were chosen and provide a rationale for why these sample sizes are sufficient.*
Data collection	*Describe the data collection procedure, including who recorded the data and how.*
Timing and spatial scale	*Indicate the start and stop dates of data collection, noting the frequency and periodicity of sampling and providing a rationale for these choices. If there is a gap between collection periods, state the dates for each sample cohort. Specify the spatial scale from which the data are taken*
Data exclusions	*If no data were excluded from the analyses, state so OR if data were excluded, describe the exclusions and the rationale behind them, indicating whether exclusion criteria were pre-established.*
Reproducibility	*Describe the measures taken to verify the reproducibility of experimental findings. For each experiment, note whether any attempts to repeat the experiment failed OR state that all attempts to repeat the experiment were successful.*
Randomization	*Describe how samples/organisms/participants were allocated into groups. If allocation was not random, describe how covariates were controlled. If this is not relevant to your study, explain why.*
Blinding	*Describe the extent of blinding used during data acquisition and analysis. If blinding was not possible, describe why OR explain why blinding was not relevant to your study.*

Did the study involve field work? ☐ Yes ☐ No

Field work, collection and transport

Field conditions	*Describe the study conditions for field work, providing relevant parameters (e.g. temperature, rainfall).*
Location	*State the location of the sampling or experiment, providing relevant parameters (e.g. latitude and longitude, elevation, water depth).*
Access & import/export	*Describe the efforts you have made to access habitats and to collect and import/export your samples in a responsible manner and in compliance with local, national and international laws, noting any permits that were obtained (give the name of the issuing authority, the date of issue, and any identifying information).*
Disturbance	*Describe any disturbance caused by the study and how it was minimized.*

Reporting for specific materials, systems and methods

We require information from authors about some types of materials, experimental systems and methods used in many studies. Here, indicate whether each material, system or method listed is relevant to your study. If you are not sure if a list item applies to your research, read the appropriate section before selecting a response.

Materials & experimental systems

n/a	Involved in the study
☐	☒ Antibodies
☐	☒ Eukaryotic cell lines
☒	☐ Palaeontology and archaeology
☒	☐ Animals and other organisms
☐	☒ Human research participants
☒	☐ Clinical data
☒	☐ Dual use research of concern

Methods

n/a	Involved in the study
☒	☐ ChIP-seq
☐	☒ Flow cytometry
☒	☐ MRI-based neuroimaging

Antibodies

Antibodies used

Flow cytometry:
Anti-human-CCR7, A-700 (Biolegend, Clone: G043H7, Cat# 353243, Lot#: B265544, Dil: 1:100)
Anti-human-CD10, PE-Cy7 (Biolegend, Clone: HI10a, Cat# 312214, Lot#: B267333, Dil: 1:100)
Anti-human-CD11c, PE-Cy5 (Biolegend, Clone: 3.9, Cat# 301610, Lot#: B291986, Dil: 1:80)
Anti-human-CD127, BV605 (Biolegend, Clone: A019D5, Cat# 351333, Lot#: B202926, Dil: 1:100)
Anti-human-CD19, APC-R700 (BD Biosciences, Clone: HIB19, Cat# 564977, Lot#: 0330615, Dil: 1:50)
Anti-human-CD19, PE/Cy5 (Biolegend, Clone: HIB19, Cat# 302216, Lot#: B263542, Dil: 1:200)
Anti-human-CD21, APC (Biolegend, Clone: Bu32, Cat# 354906, Lot#: B284909, Dil: 1:60)
Anti-human-CD21, PE-CF594 (Biolegend, Clone: Bu32, Cat# 354922, Lot#: B266487, Dil: 1:125)
Anti-human-CD24, BV510 (Biolegend, Clone: ML5, Cat# 311126, Lot#: B256187, Dil: 1:250)
Anti-human-CD25, PE (Biolegend, Clone: BC96, Cat# 302606, Lot#: B296102, Dil: 1:25)
Anti-human-CD27, BV421 (Biolegend, Clone: O323, Cat# 302824, Lot#: B301442, Dil: 1:100)
Anti-human-CD27, PE (Biolegend, Clone: O323, Cat# 302808, Lot#: B236590, Dil: 1:100)
Anti-human-CD38, PerCP-Cy5.5 (Biolegend, Clone: HIT2, Cat# 303421, Lot#: B168290, Dil: 1:250)
Anti-human-CD38, FITC (Biolegend, Clone: HB-7, Cat# 356610, Lot#: B178147, Dil: 1:100)
Anti-human-CD38, PE (Biolegend, Clone: HIT2, Cat# 303506, Lot#: B273752, Dil: 1:250)
Anti-human-CD38, BB700 (BD Biosciences, Clone: HIT2, Cat# 566445, Lot#: 0220490, Dil: 1:166)
Anti-human-CD4, FITC (Biolegend, Clone: RPA-T4, Cat# 300506, Lot#: B175118, Dil: 1:200)
Anti-human-CD45RA, PE-Cy5 (Biolegend, Clone: HI100, Cat# 304110, Lot#: B204601, Dil: 1:250)
Anti-human-CD69, PE-Cy7 (Biolegend, Clone: FN50, Cat# 310912, Lot#: B259693, Dil: 1:50)
Anti-human-CD80, FITC (Biolegend, Clone: 2D10, Cat# 305205, Lot#: B273868, Dil: 1:80)
Anti-human-CD86, APC (Biolegend, Clone: IT2.2, Cat# 305412, Lot#: B173001, Dil: 1:50)
Anti-human-CD138, BV605 (Biolegend, Clone: MI15, Cat# 356520, Lot#: B284321, Dil: 1:100)
Anti-human-CXCR5, PE (Biolegend, Clone: J252D4, Cat# 356904, Lot#: B283577, Dil: 1:200)
Anti-human-CXCR5, BV421 (Biolegend, Clone: J252D4, Cat# 356920, Lot#: B252332, Dil: 1:500)
Anti-human-HLA-DR, PE-Cy5 (Biolegend, Clone: L243, Cat# 307607, Lot#: B283127, Dil: 1:500)
Anti-human-ICOS, APC (Biolegend, Clone: C398.4A, Cat# 313509, Lot#: B248382, Dil: 1:200)
Anti-human-IgD, A-700 (Biolegend, Clone: IA6-2, Cat# 348229, Lot#: B222292, Dil: 1:125)
Anti-human-IgD, BV605 (Biolegend, Clone: IA6-2, Cat# 348232, Lot#: B277503, Dil: 1:250)
Anti-human-IgG, BB515 (BD Biosciences, Clone: G18-145, Cat# 564581, Lot#: 8303637, Dil: 1:500)
Anti-human-IgG, FITC (Biolegend, Clone: HP6017, Cat# 409310, Lot#: B272569, Dil: 1:250)
Anti-human-IgM, PE-CF594 (BD Biosciences, Clone: G20-127, Cat# 562539, Lot#: 0098775, Dil: 1:100)
Anti-human-IgM, PerCP-Cy5.5 (Biolegend, Clone: MHM-88, Cat# 314511, Lot#: B268270, Dil: 1:250)
Anti-human-PD-1, PE-Cy7 (Biolegend, Clone: EH12.2H7, Cat# 329918, Lot#: B298289, Dil: 1:80)
Anti-human-IFNy, PE-Cy7 (Biolegend, Clone: B27, Cat# 506518, Lot#: B182253, Dil: 1:125)
Anti-human-IL-10, PE-Cy7 (Biolegend, Clone: JES3-9D7, Cat# 501419, Lot#: B308621, Dil: 1:100)
Anti-human-TNF-a, PerCP-Cy5.5 (Biolegend, Clone: MAb11, Cat# 502926, Lot#: B171068, Dil: 1:200)
Anti-human-IL-21, APC (Biolegend, Clone: 3A3-N2, Cat# 513007, Lot#: B256205, Dil: 1:100)

Anti-human-CD3, BB515 (BD Biosciences, Clone: UCHT1, Cat# 564466, Lot#: 6315656, Dil: 1:100)
Anti-human-IFN-γ, APC Biolegend, Clone: B27, Cat#: 506510, Lot# B237406, Dil: 1:200)
Anti-human-TNF-, BV510 (Biolegend, Clone: Mab11, Cat#: 502949, Lot# B339729, Dil: 1:200)
Anti-human-IL-21, PE (Biolegend, Clone: 3A3-N2, Cat#: 513004, Lot# B335103, Dil: 1:100)
Anti-human-CD4, BV605 (Biolegend, Clone: RPA-T4, Cat#: 300556, Lot# B289706, Dil: 1:200)
Anti-human-CD19, PE-Cy7 (Biolegend, Clone: HIB19, Cat#: 302216, Lot# B172415, Dil: 1:200)
Anti-human-CD25, FITC (Biolegend, Clone: BC96, Cat#: 302604, Lot# B339583, Dil: 1:100)
Anti-human-T-bet, PE-Cy7 (Biolegend, Clone: 4B10, Cat#: 644824, Lot# B331455, Dil: 1:100)
Anti-human-FcRL4, PE (Biolegend, Clone: 413D12, Cat#: 340203, Lot# B248797, Dil: 1:50)
Anti-human-FcRL5, APC (Biolegend, Clone: 509f6, Cat#: 340305, Lot# B309350, Dil: 1:50)
Anti-human-CD85j, APC (Biolegend, Clone: GHI/75, Cat#: 333719, Lot# B279700, Dil: 1:50)
Anti-human-CXCR3, Alexa-Fluor-700 (Biolegend, Clone: G025H7, Cat#: 353741, Lot# B271260, Dil: 1:100)
Anti-human-CD95, Alexa-Fluor-700 (Biolegend, Clone: DX2, Cat#: 305647, Lot# B275145, Dil: 1:00)
Anti-human-BAFF-R, PE-Cy7 (Biolegend, Clone: 11C1, Cat#: 316919, Lot# B294228, Dil: 1:125)
Anti-human-IgL, FITC (Biolegend, Clone: MHL-38, Cat#: 316606, Lot# B207106, Dil: 1:200)
Anti-human-IgK, Pacific Blue (Biolegend, Clone: MHK-49, Cat#: 316524, Lot# B160046, Dil: 1:200)

Confocal Microscopy:
Anti-human-CD4, Alexa-Fluor-488 (Abcam, Clone: EPR6855, Cat#: ab196372, Dil: 1:100)
Anti-human-CD19, Alexa-Fluor-555 (Abcam, Clone: EPR5906, Cat#: ab274888, Dil: 1:100)
Anti-human-T-bet, Alexa-Fluor-647 (Abcam, Clone: EPR9301, Cat#: ab225198, Dil: 1:100)
Anti-human-PD-1, Alexa-Fluor-647 (Abcam, Clone: EPR4877(2), Cat#: ab201825, Dil: 1:100)

B cell enrichment:
Anti-human CD20 magnetic beads (Miltenyi Biotec, Cat# 130-091-104)

ELISA:
9G4 Human - Purified rat anti-idiotypic antibody to VH4-34 gene-encoded (IgM Biosciences, Dil: 1:500)
Goat-anti-Human IgM Secondary Antibody, HRP, (Thermo Fisher Scientific, Cat# 31415, Lot#: OL1793879, Dil: 1:4000)
Goat-anti-Human IgG F(ab')2 Secondary Antibody, HRP, , (Thermo Fisher Scientific, Cat# 31414, Lot#: TH2622475, Dil: 1:5000)

Immunohistochemistry:
Anti-human-CD21 (Cell Marque Tissue Diagnostics, 2G9 Mouse Monoclonal, Cat# 760-4245, conc. 0.5 µg/mL)
Anti-human-CD20 (Roche Diagnostics, L26 Mouse Monoclonal, Cat# 760-2531, conc. 0.3 µg/mL)
Anti-human-BCL-2 (Roche Diagnostics, I24 Mouse Monoclonal, Cat# 790-4464, conc. 2.62 µg/mL)
Anti-human-CD4 (Roche Diagnostics, SP35 Rabbit Monoclonal, Cat# 790-4423, conc. 2.5 µg/mL.)

Validation

In the study we only used commercially available antibodies validated by the manufacturer. All antibody validation is provided on supplier website. In case of flow cytometry, optimal dilution for each antibody used was determined by single staining of the target lymphocytes (human B or T cells) with serial dilution of the given antibody. Optimal dilutions for HRP-conjugated secondary antibodies were determined experimentally with ELISA.

Eukaryotic cell lines

Policy information about cell lines

Cell line source(s)

HEK293T cell line was purchased (ATCC, #CRL-3216) and kindly provided by Dr. Donna Eason (University of South Florida).

Authentication

HEK293T cell line was authenticated prior to receipt by the commercial vendor.

Mycoplasma contamination

HEK293T cell line was not tested for mycoplasma.

Commonly misidentified lines
(See ICLAC register)

No commonly misidentified cell lines were used.

Palaeontology and Archaeology

Specimen provenance

Provide provenance information for specimens and describe permits that were obtained for the work (including the name of the issuing authority, the date of issue, and any identifying information). Permits should encompass collection and, where applicable, export.

Specimen deposition

Indicate where the specimens have been deposited to permit free access by other researchers.

Dating methods

If new dates are provided, describe how they were obtained (e.g. collection, storage, sample pretreatment and measurement), where they were obtained (i.e. lab name), the calibration program and the protocol for quality assurance OR state that no new dates are provided.

☐ Tick this box to confirm that the raw and calibrated dates are available in the paper or in Supplementary Information.

Ethics oversight	*Identify the organization(s) that approved or provided guidance on the study protocol, OR state that no ethical approval or guidance was required and explain why not.*

Note that full information on the approval of the study protocol must also be provided in the manuscript.

Animals and other organisms

Policy information about studies involving animals; ARRIVE guidelines recommended for reporting animal research

Laboratory animals	*For laboratory animals, report species, strain, sex and age OR state that the study did not involve laboratory animals.*
Wild animals	*Provide details on animals observed in or captured in the field; report species, sex and age where possible. Describe how animals were caught and transported and what happened to captive animals after the study (if killed, explain why and describe method; if released, say where and when) OR state that the study did not involve wild animals.*
Field-collected samples	*For laboratory work with field-collected samples, describe all relevant parameters such as housing, maintenance, temperature, photoperiod and end-of-experiment protocol OR state that the study did not involve samples collected from the field.*
Ethics oversight	*Identify the organization(s) that approved or provided guidance on the study protocol, OR state that no ethical approval or guidance was required and explain why not.*

Note that full information on the approval of the study protocol must also be provided in the manuscript.

Human research participants

Policy information about studies involving human research participants

Population characteristics	Population characteristics are detailed in Supplementary Table 1, RAG1/2 variants are detailed in Supplementary Table 2.
Recruitment	16 patients carrying pathogenic or likely pathogenic hypomorphic RAG1 or 2 gene variants were recruited to our study. All except an asymptomatic infant (P1), displayed clinical phenotype of combined immune deficiency with granuloma/ autoimmunity phenotype according to the enrollment criteria described in the Methods. Peripheral blood sample was available from 15 patients; spleen histology sections were available from one patient. No self-selection bias occurred. A patient was enrolled to the study after referral from collaborating physicians, if met the enrollment criteria.<br>27 healthy subjects representing age groups corresponding to patient samples were enrolled in the study according to the enrollment criteria described in the Methods.
Ethics oversight	The study was approved by the local ethics committee of the University of South Florida (USF-Pro00035468, USF-Pro00025693) and Johns Hopkins Medical Institute / Johns Hopkins All Children's Hospital (JHMI-IRB00175372, JHMI-IRB00097062, JHMI-IRB00097938). All protocols followed local ethics recommendations and informed, written consent was obtained from all participants, parents or legally authorized representative.

Note that full information on the approval of the study protocol must also be provided in the manuscript.

Clinical data

Policy information about clinical studies

All manuscripts should comply with the ICMJE guidelines for publication of clinical research and a completed CONSORT checklist must be included with all submissions.

Clinical trial registration	*Provide the trial registration number from ClinicalTrials.gov or an equivalent agency.*
Study protocol	*Note where the full trial protocol can be accessed OR if not available, explain why.*
Data collection	*Describe the settings and locales of data collection, noting the time periods of recruitment and data collection.*
Outcomes	*Describe how you pre-defined primary and secondary outcome measures and how you assessed these measures.*

Dual use research of concern

Policy information about dual use research of concern

Hazards

Could the accidental, deliberate or reckless misuse of agents or technologies generated in the work, or the application of information presented in the manuscript, pose a threat to:

No | Yes
- [ ] [ ] Public health
- [ ] [ ] National security
- [ ] [ ] Crops and/or livestock
- [ ] [ ] Ecosystems
- [ ] [ ] Any other significant area

Experiments of concern

Does the work involve any of these experiments of concern:

No | Yes
- [ ] [ ] Demonstrate how to render a vaccine ineffective
- [ ] [ ] Confer resistance to therapeutically useful antibiotics or antiviral agents
- [ ] [ ] Enhance the virulence of a pathogen or render a nonpathogen virulent
- [ ] [ ] Increase transmissibility of a pathogen
- [ ] [ ] Alter the host range of a pathogen
- [ ] [ ] Enable evasion of diagnostic/detection modalities
- [ ] [ ] Enable the weaponization of a biological agent or toxin
- [ ] [ ] Any other potentially harmful combination of experiments and agents

ChIP-seq

Data deposition

- [ ] Confirm that both raw and final processed data have been deposited in a public database such as GEO.
- [ ] Confirm that you have deposited or provided access to graph files (e.g. BED files) for the called peaks.

Data access links<br>*May remain private before publication.*	*For "Initial submission" or "Revised version" documents, provide reviewer access links. For your "Final submission" document, provide a link to the deposited data.*
Files in database submission	*Provide a list of all files available in the database submission.*
Genome browser session<br>(e.g. UCSC)	*Provide a link to an anonymized genome browser session for "Initial submission" and "Revised version" documents only, to enable peer review. Write "no longer applicable" for "Final submission" documents.*

Methodology

Replicates	*Describe the experimental replicates, specifying number, type and replicate agreement.*
Sequencing depth	*Describe the sequencing depth for each experiment, providing the total number of reads, uniquely mapped reads, length of reads and whether they were paired- or single-end.*
Antibodies	*Describe the antibodies used for the ChIP-seq experiments; as applicable, provide supplier name, catalog number, clone name, and lot number.*
Peak calling parameters	*Specify the command line program and parameters used for read mapping and peak calling, including the ChIP, control and index files used.*
Data quality	*Describe the methods used to ensure data quality in full detail, including how many peaks are at FDR 5% and above 5-fold enrichment.*
Software	*Describe the software used to collect and analyze the ChIP-seq data. For custom code that has been deposited into a community repository, provide accession details.*

Flow Cytometry

Plots

Confirm that:

☒ The axis labels state the marker and fluorochrome used (e.g. CD4-FITC).

☒ The axis scales are clearly visible. Include numbers along axes only for bottom left plot of group (a 'group' is an analysis of identical markers).

☒ All plots are contour plots with outliers or pseudocolor plots.

☒ A numerical value for number of cells or percentage (with statistics) is provided.

Methodology

Sample preparation	Sample preparation is described in the Methods section.
Instrument	FACSAria II (BD Biosciences)
Software	FACSDiva software version 10 was used for data acquisition. The analysis of flow cytometry data was performed using FlowJo software (version 10.7.1) and plugins FlowSOM (v2.5), tSNE (v2.0), Hyperfinder (v0.6.2) and DownSample (v3.0) FlowJo.
Cell population abundance	Bulk cell sorts were performed using 4-Way Purity precision mode. For BCR repertoire sequencing, 25-50K specific B cells were sorted from each subject and used for RNA isolation. For in vitro B cell activation and B cell – Tfh cell co-culture assays, 100K-200K B and Tfh cells were sorted from each subject and cultured as described in the Methods. Purity of post-sort fractions were determined by flow cytometry ensuring a purity of >95% defined as the ratio of target cells and total cells. Single cell sorts were performed using Single Cell precision mode.
Gating strategy	In all experiments human peripheral blood-derived B or T lymphocytes or in vitro cultured B lymphocytes were used. Pre-gating was performed on single cells using FSC-A/FSC-H, then on lymphocytes using FSC-H/SSC-H, followed by cell debris and dead cell exclusion using fixable viability dye eFluor-780 (ThermoFisher Scientific). Total B cells were defined by the mean of CD19 expression, CD4 helper T cells were defined by the mean of CD4 expression. For specific B and T subset identification, individual gating strategies are described in the related Methods sections and the figure legends for each experiment. The detailed gating strategy for the sixteen B cell subsets defined in the manuscript is shown in Supplementary Figure 3.

☒ Tick this box to confirm that a figure exemplifying the gating strategy is provided in the Supplementary Information.

Magnetic resonance imaging

Experimental design

Design type	*Indicate task or resting state; event-related or block design.*
Design specifications	*Specify the number of blocks, trials or experimental units per session and/or subject, and specify the length of each trial or block (if trials are blocked) and interval between trials.*
Behavioral performance measures	*State number and/or type of variables recorded (e.g. correct button press, response time) and what statistics were used to establish that the subjects were performing the task as expected (e.g. mean, range, and/or standard deviation across subjects).*

Acquisition

Imaging type(s)	*Specify: functional, structural, diffusion, perfusion.*
Field strength	*Specify in Tesla*
Sequence & imaging parameters	*Specify the pulse sequence type (gradient echo, spin echo, etc.), imaging type (EPI, spiral, etc.), field of view, matrix size, slice thickness, orientation and TE/TR/flip angle.*
Area of acquisition	*State whether a whole brain scan was used OR define the area of acquisition, describing how the region was determined.*

Diffusion MRI ☐ Used ☐ Not used

Preprocessing

Preprocessing software	*Provide detail on software version and revision number and on specific parameters (model/functions, brain extraction, segmentation, smoothing kernel size, etc.).*
Normalization	*If data were normalized/standardized, describe the approach(es): specify linear or non-linear and define image types used for transformation OR indicate that data were not normalized and explain rationale for lack of normalization.*

Normalization template	*Describe the template used for normalization/transformation, specifying subject space or group standardized space (e.g. original Talairach, MNI305, ICBM152) OR indicate that the data were not normalized.*
Noise and artifact removal	*Describe your procedure(s) for artifact and structured noise removal, specifying motion parameters, tissue signals and physiological signals (heart rate, respiration).*
Volume censoring	*Define your software and/or method and criteria for volume censoring, and state the extent of such censoring.*

Statistical modeling & inference

Model type and settings	*Specify type (mass univariate, multivariate, RSA, predictive, etc.) and describe essential details of the model at the first and second levels (e.g. fixed, random or mixed effects; drift or auto-correlation).*
Effect(s) tested	*Define precise effect in terms of the task or stimulus conditions instead of psychological concepts and indicate whether ANOVA or factorial designs were used.*

Specify type of analysis: ☐ Whole brain ☐ ROI-based ☐ Both

Statistic type for inference<br>(See Eklund et al. 2016)	*Specify voxel-wise or cluster-wise and report all relevant parameters for cluster-wise methods.*
Correction	*Describe the type of correction and how it is obtained for multiple comparisons (e.g. FWE, FDR, permutation or Monte Carlo).*

Models & analysis

n/a	Involved in the study
☐	☐ Functional and/or effective connectivity
☐	☐ Graph analysis
☐	☐ Multivariate modeling or predictive analysis

Functional and/or effective connectivity	*Report the measures of dependence used and the model details (e.g. Pearson correlation, partial correlation, mutual information).*
Graph analysis	*Report the dependent variable and connectivity measure, specifying weighted graph or binarized graph, subject- or group-level, and the global and/or node summaries used (e.g. clustering coefficient, efficiency, etc.).*
Multivariate modeling and predictive analysis	*Specify independent variables, features extraction and dimension reduction, model, training and evaluation metrics.*
