# Supplementary figures and images for: Partial RAG deficiency in humans induces dysregulated peripheral lymphocyte development and humoral tolerance defect with accumulation of T-bet+ B cells
Source: Nat Immunol. 2022 Jul 28;23(8):1256–72. doi: 10.1038/s41590-022-01271-6 (PMC9355881; doi:10.1038/s41590-022-01271-6)

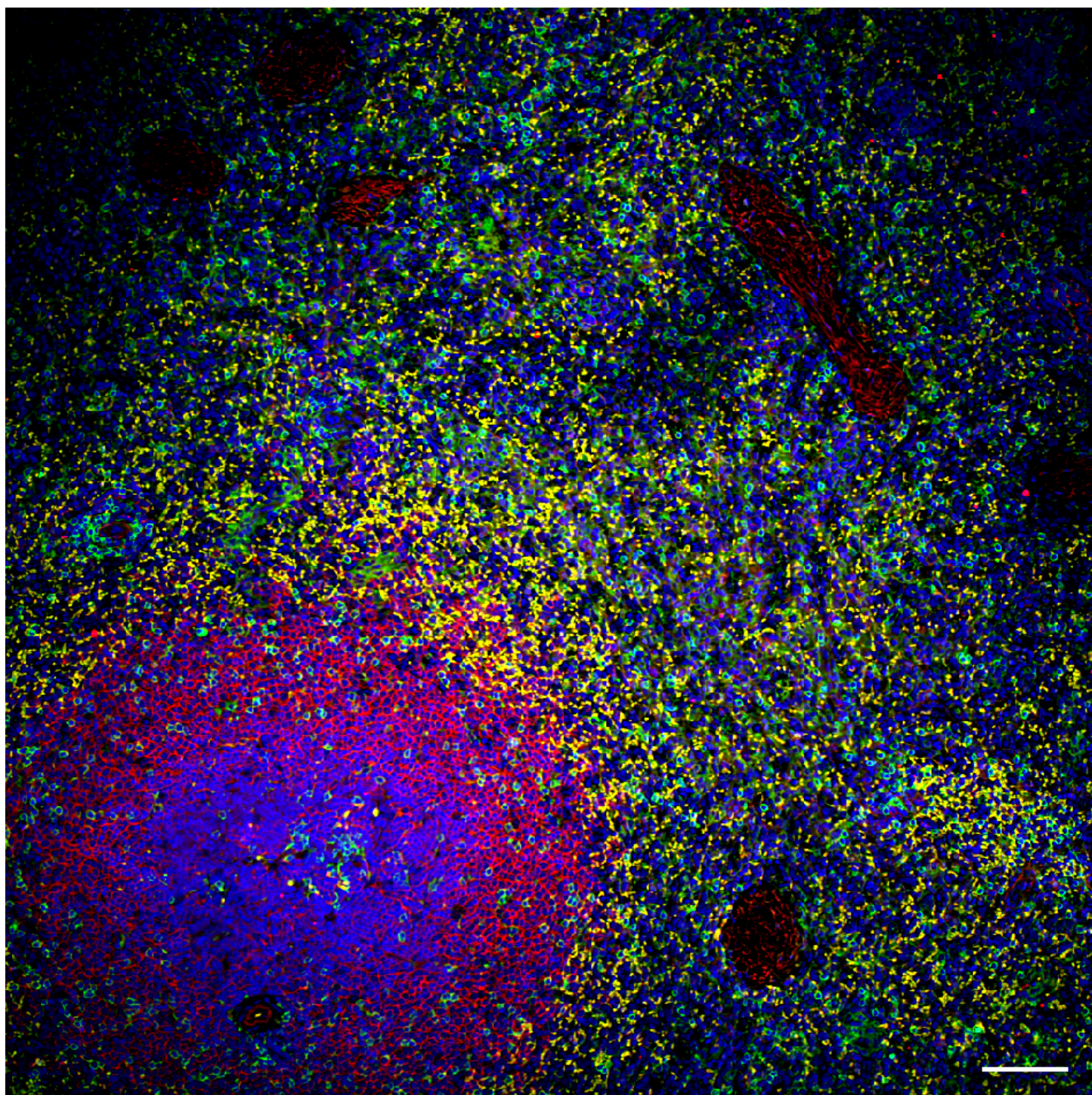

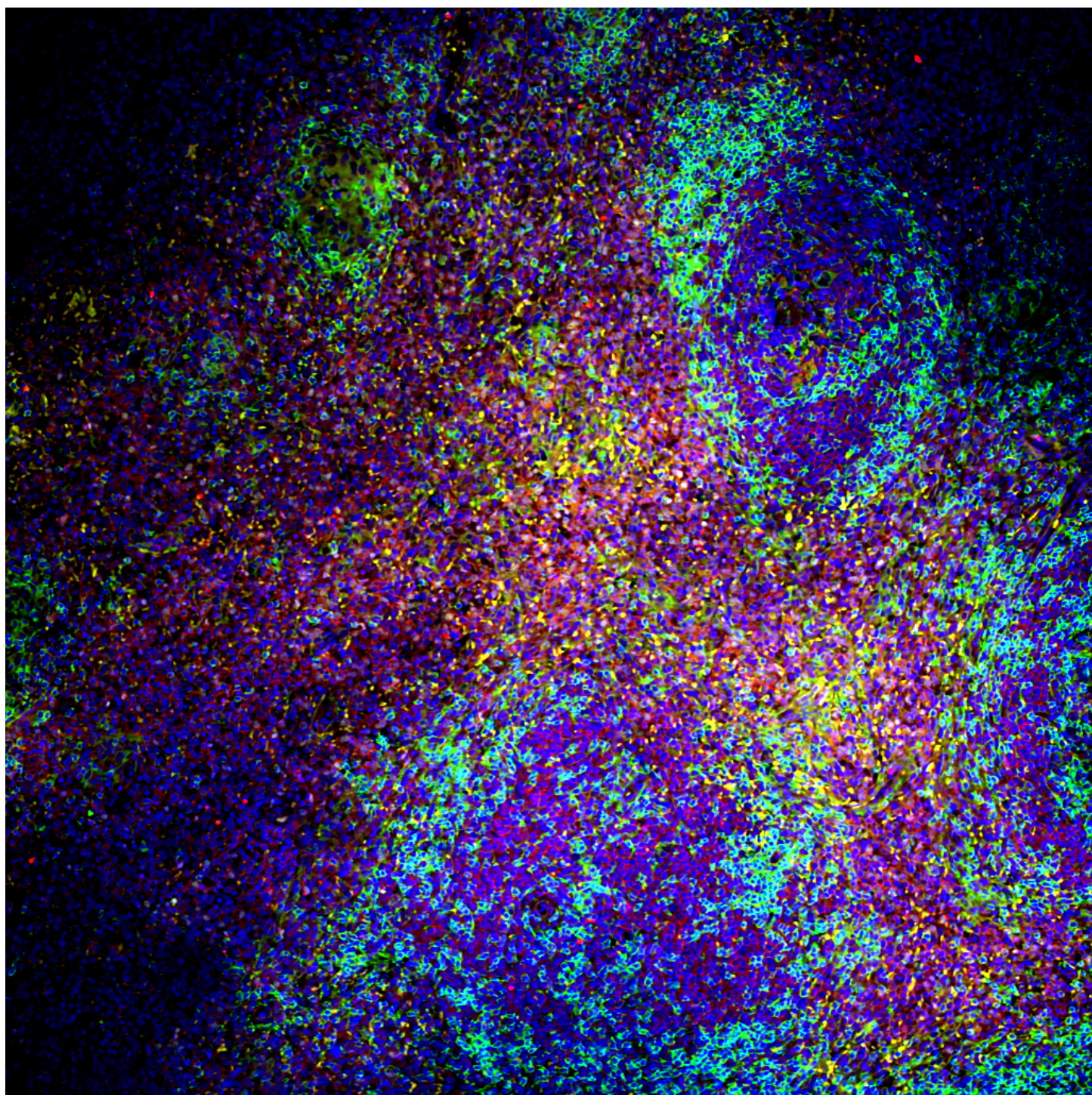

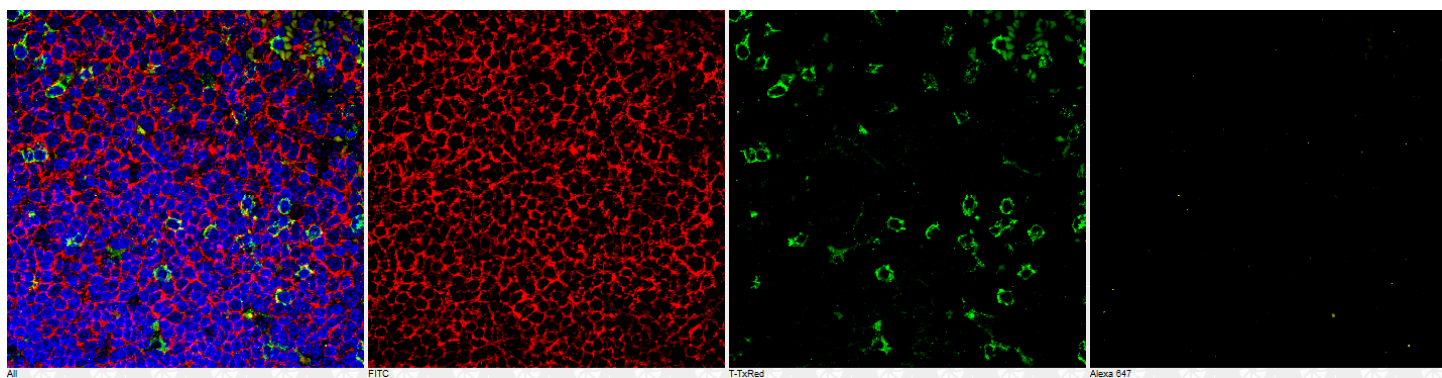

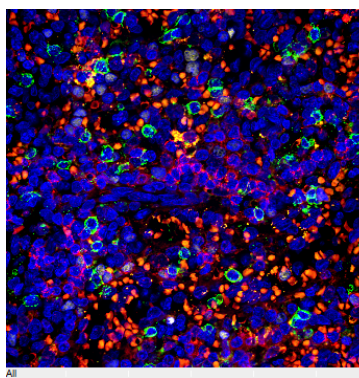

All

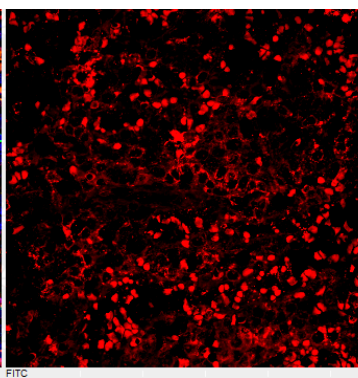

FITC

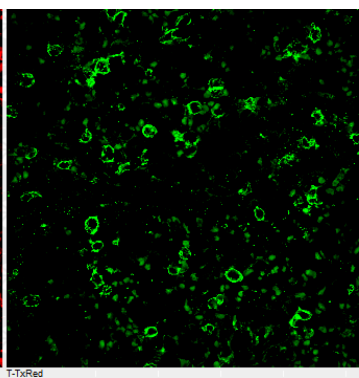

T-TxRed

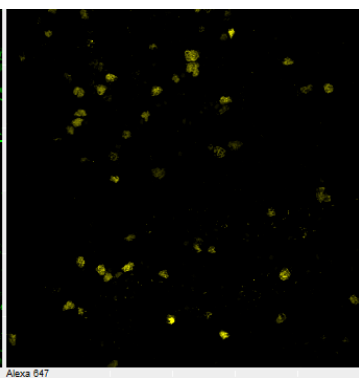

Alexa 647

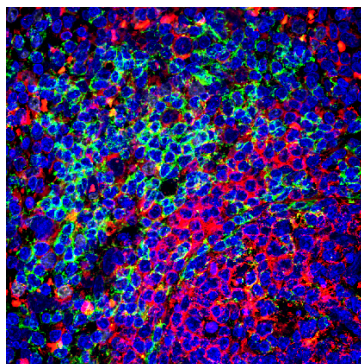

All

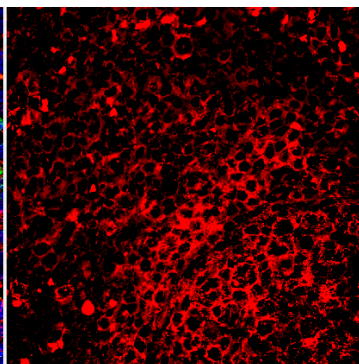

FITC

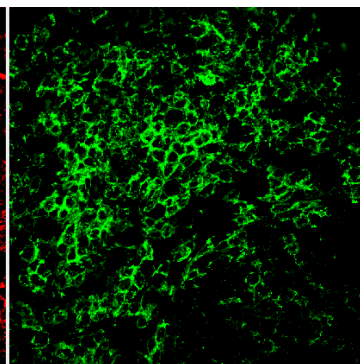

T-1aRed

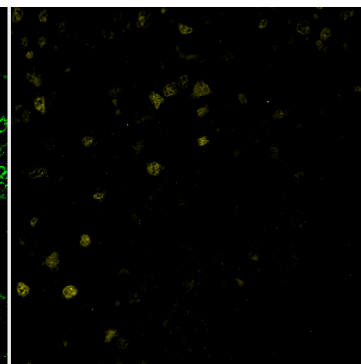

Alexa 547

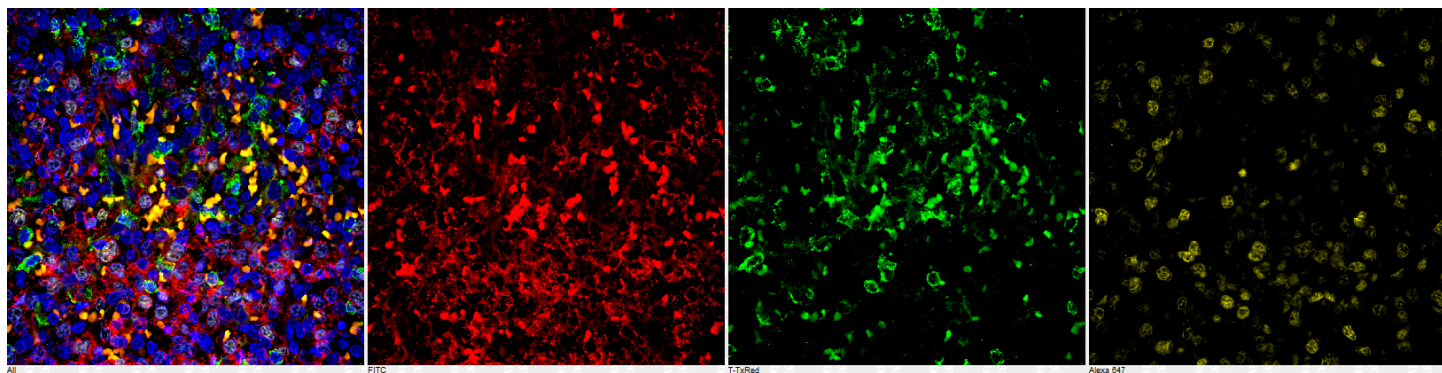

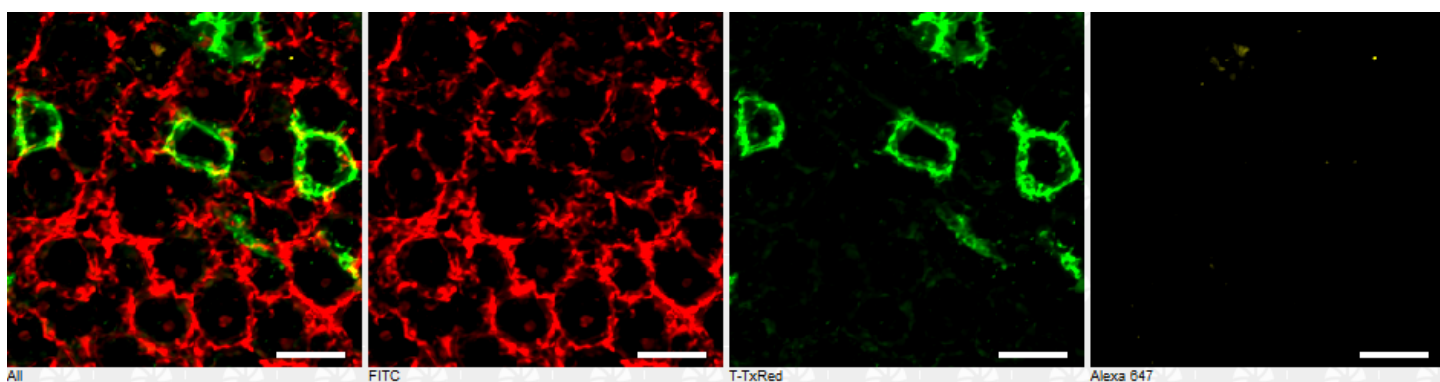

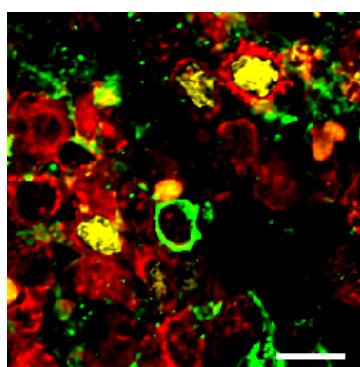

All

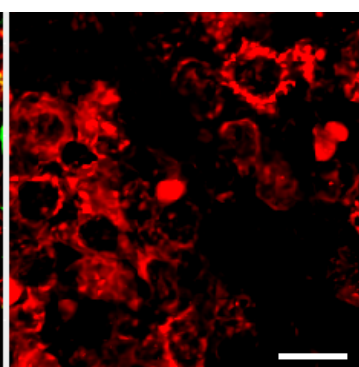

FITC

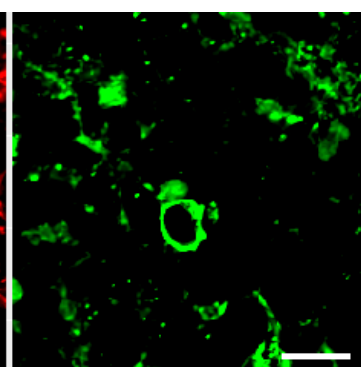

T-TxRed

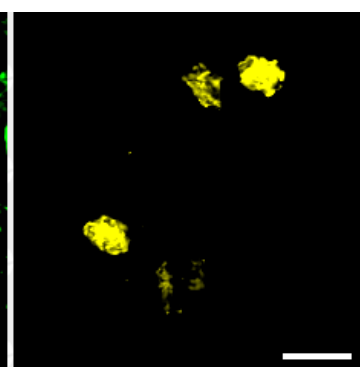

Alexa 647

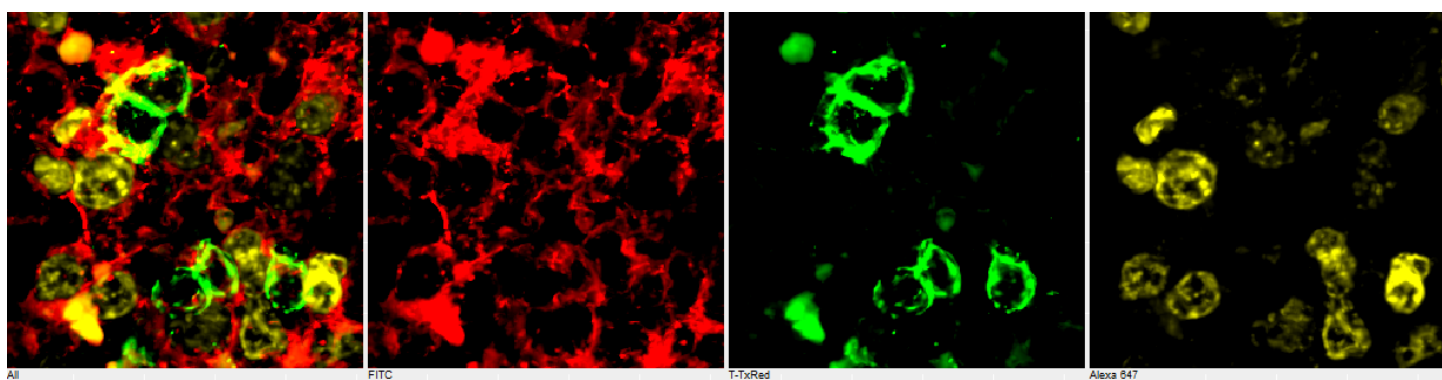

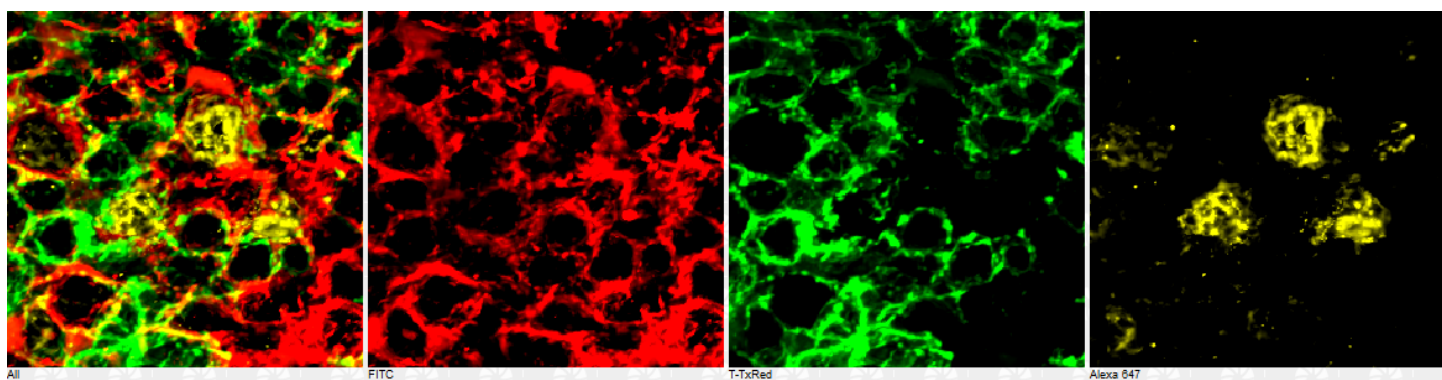

Supplement: Source Data Fig. 2 — Image Source Data. [file 41590_2022_1271_MOESM5_ESM.pdf]

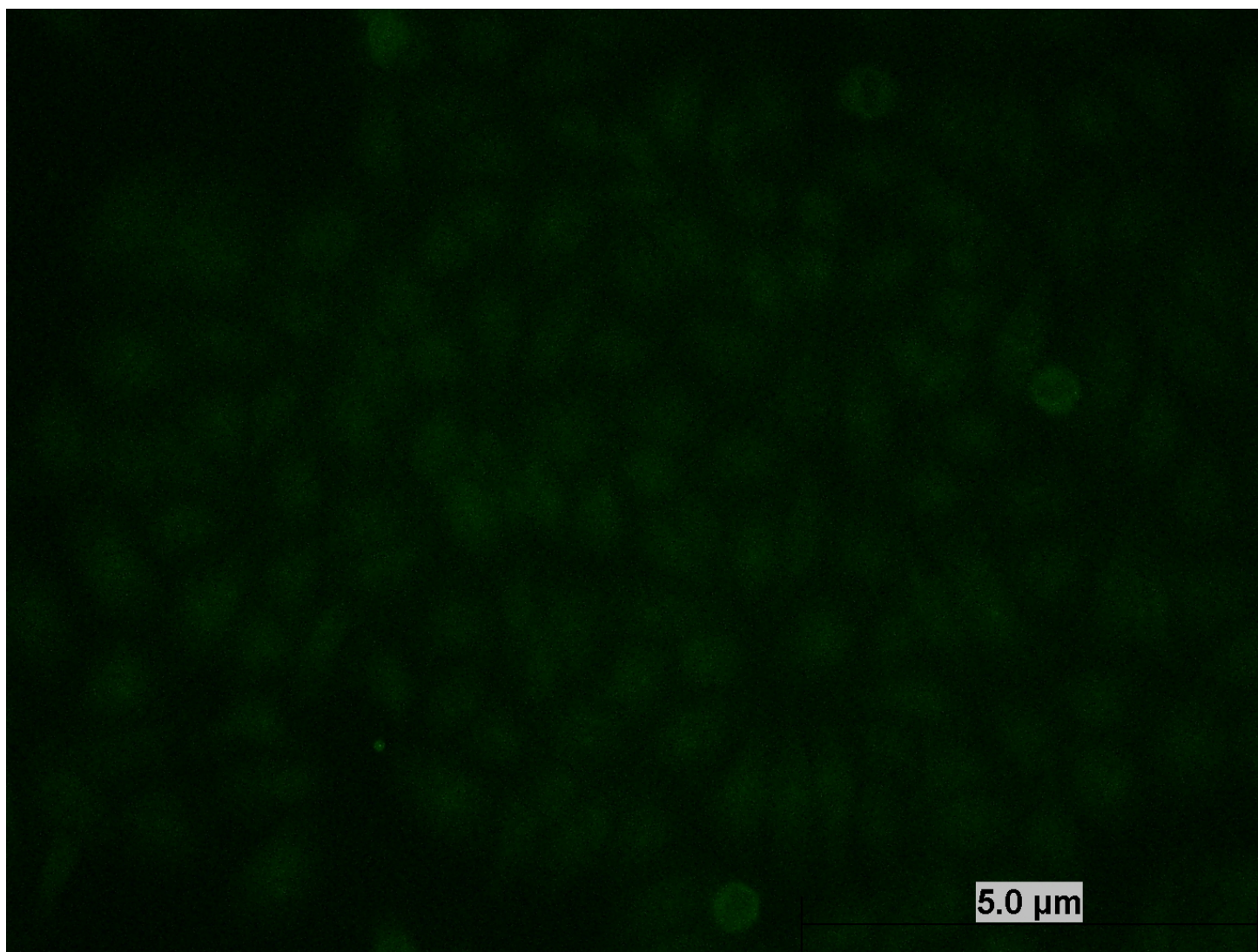

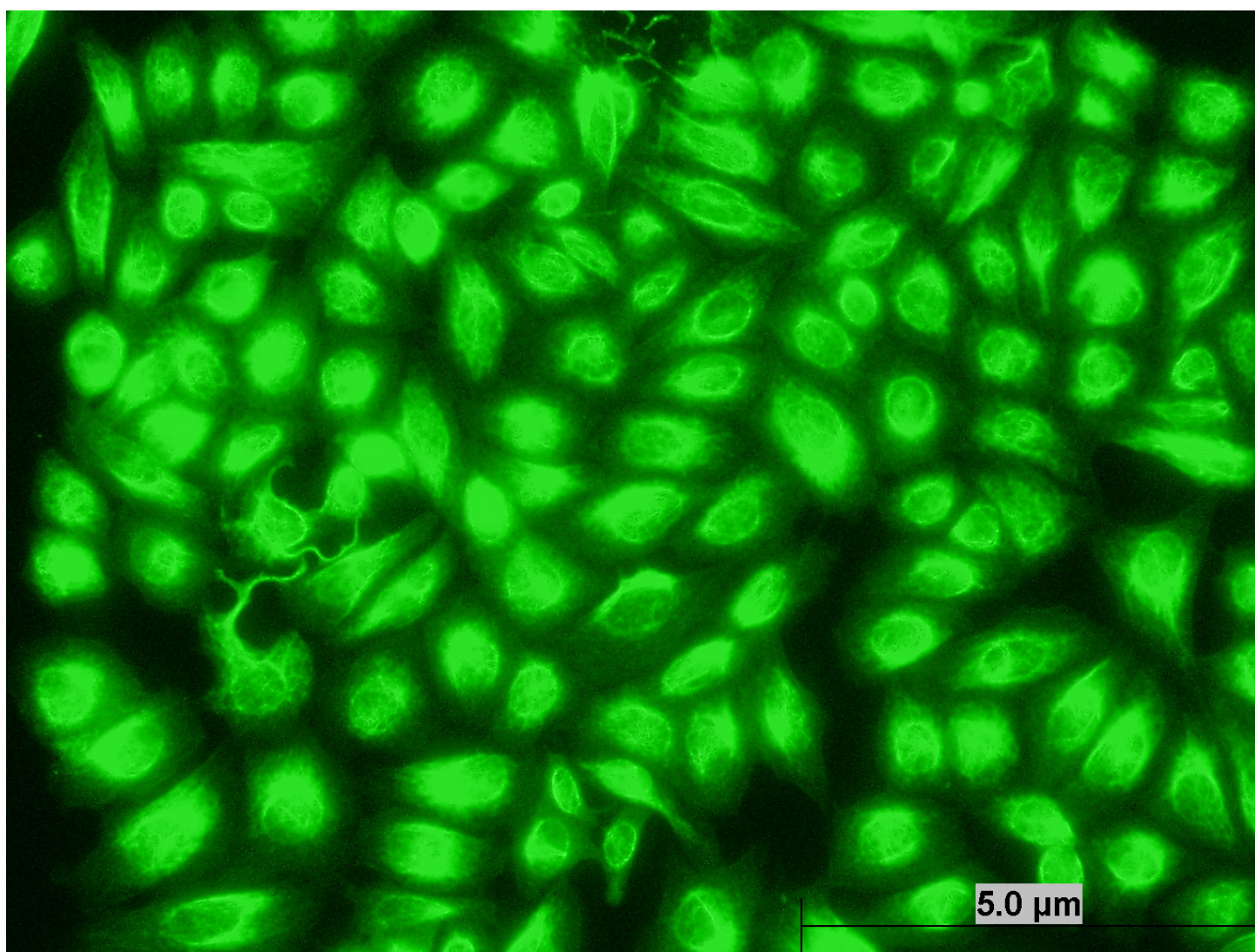

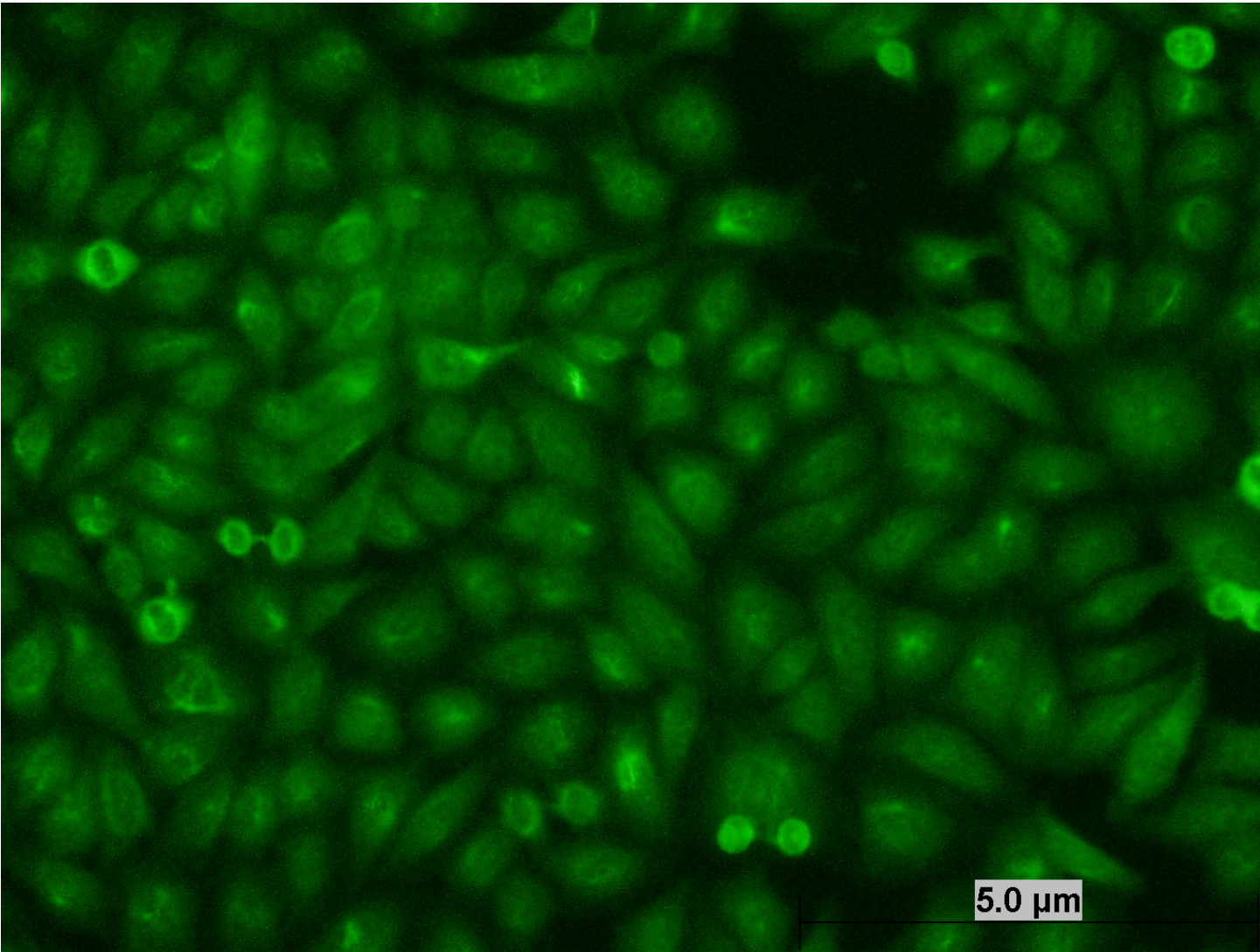

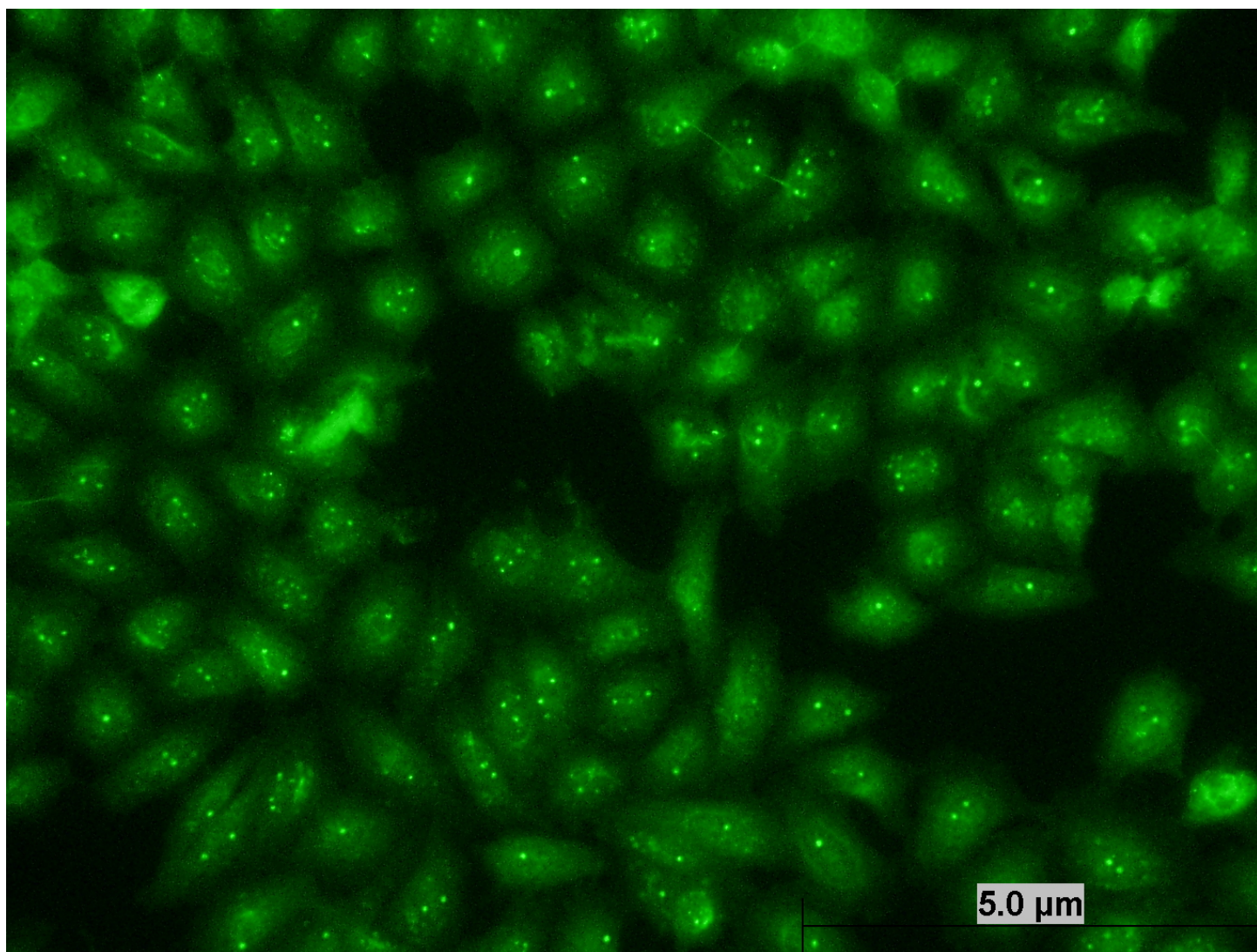

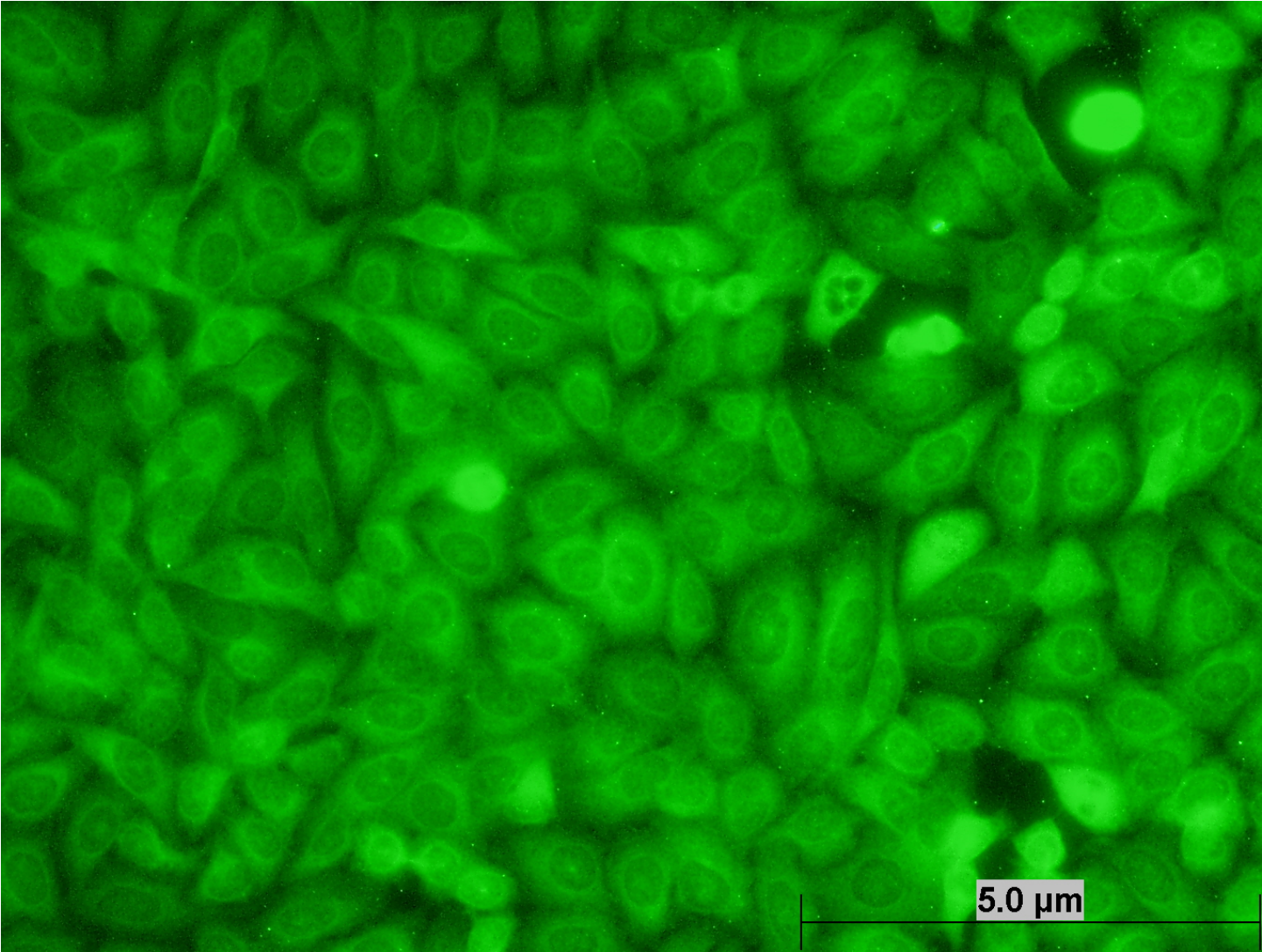

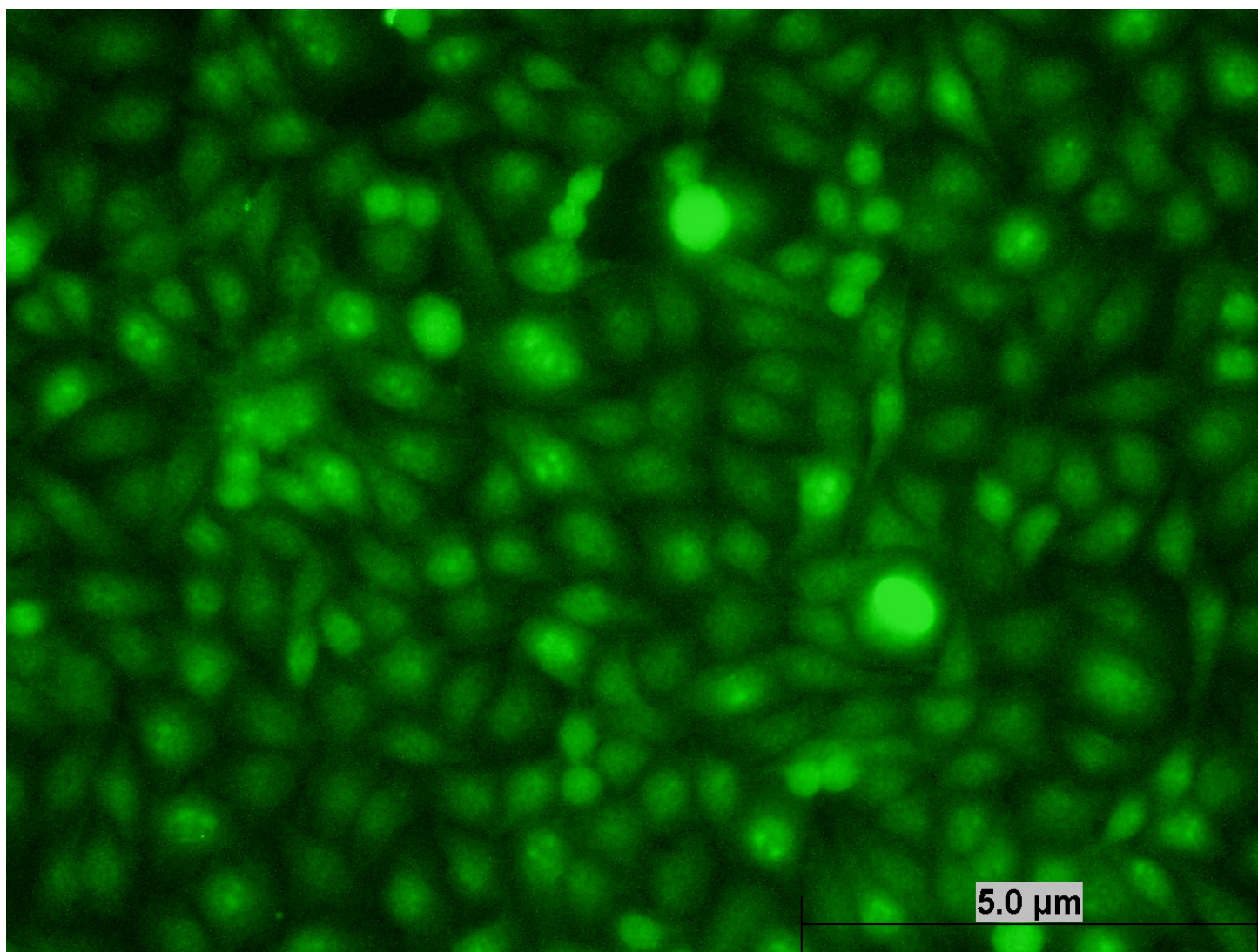

Supplement: Source Data Fig. 4 — Image Source Data. [file 41590_2022_1271_MOESM8_ESM.pdf]

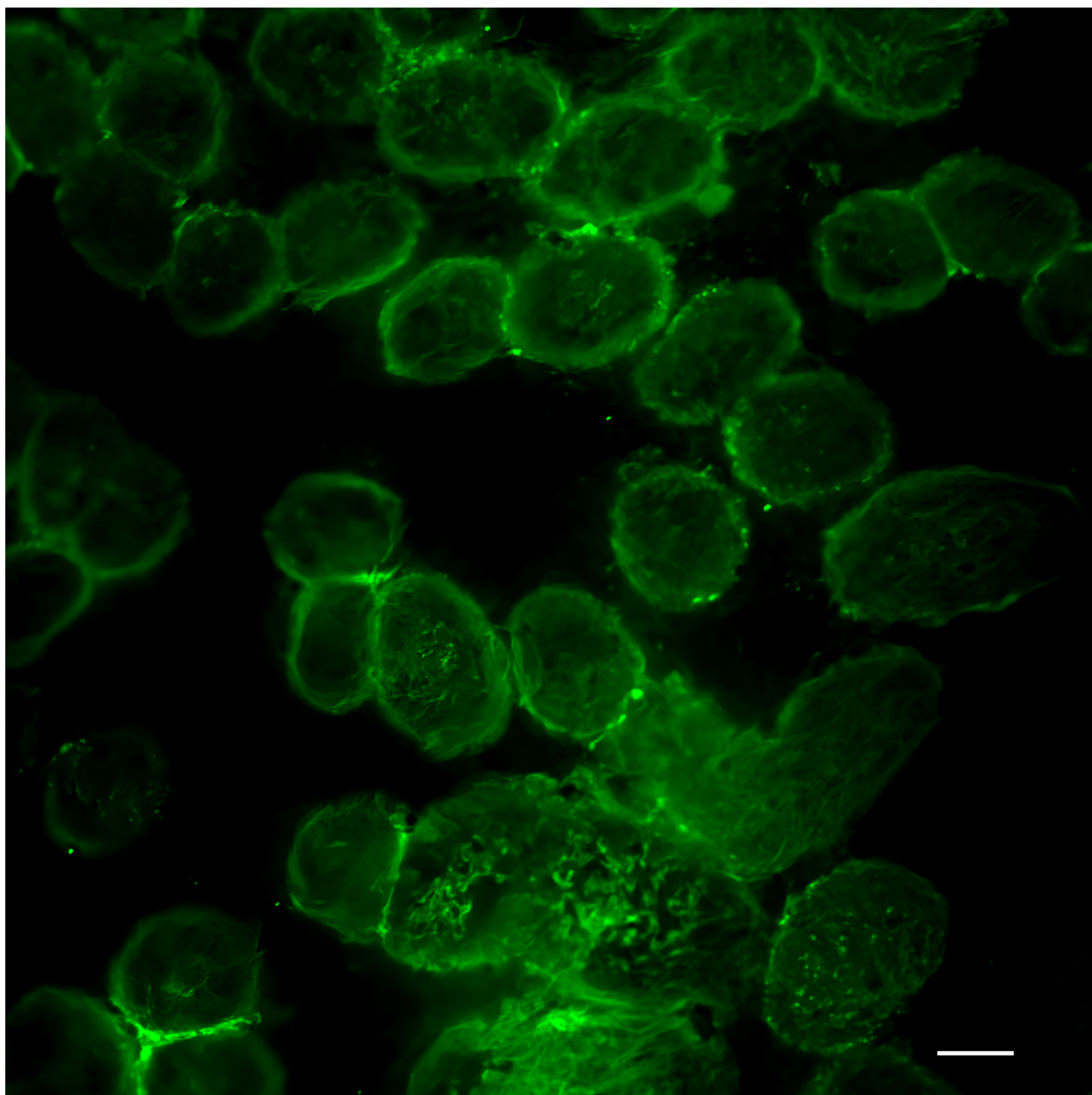

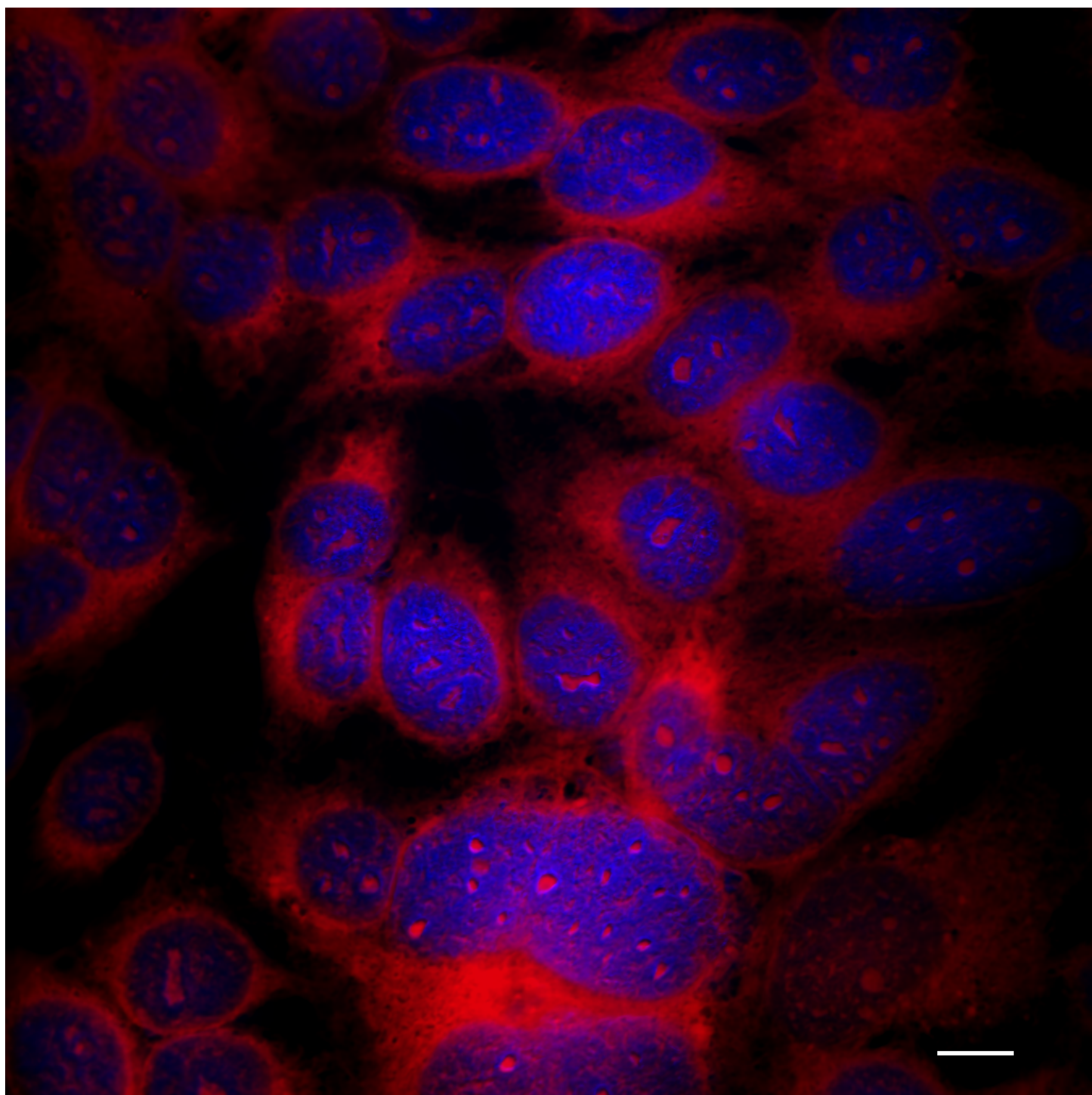

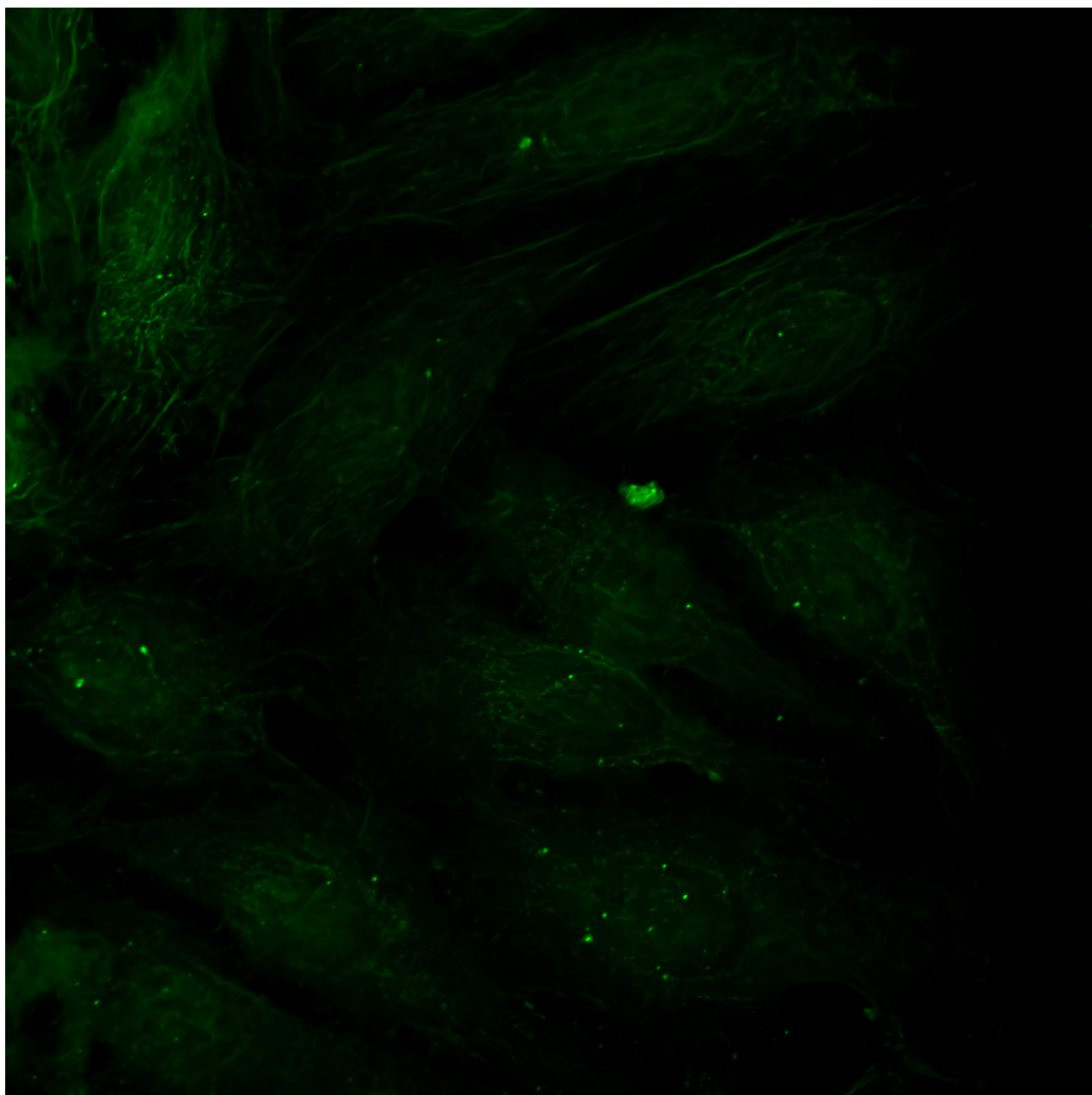

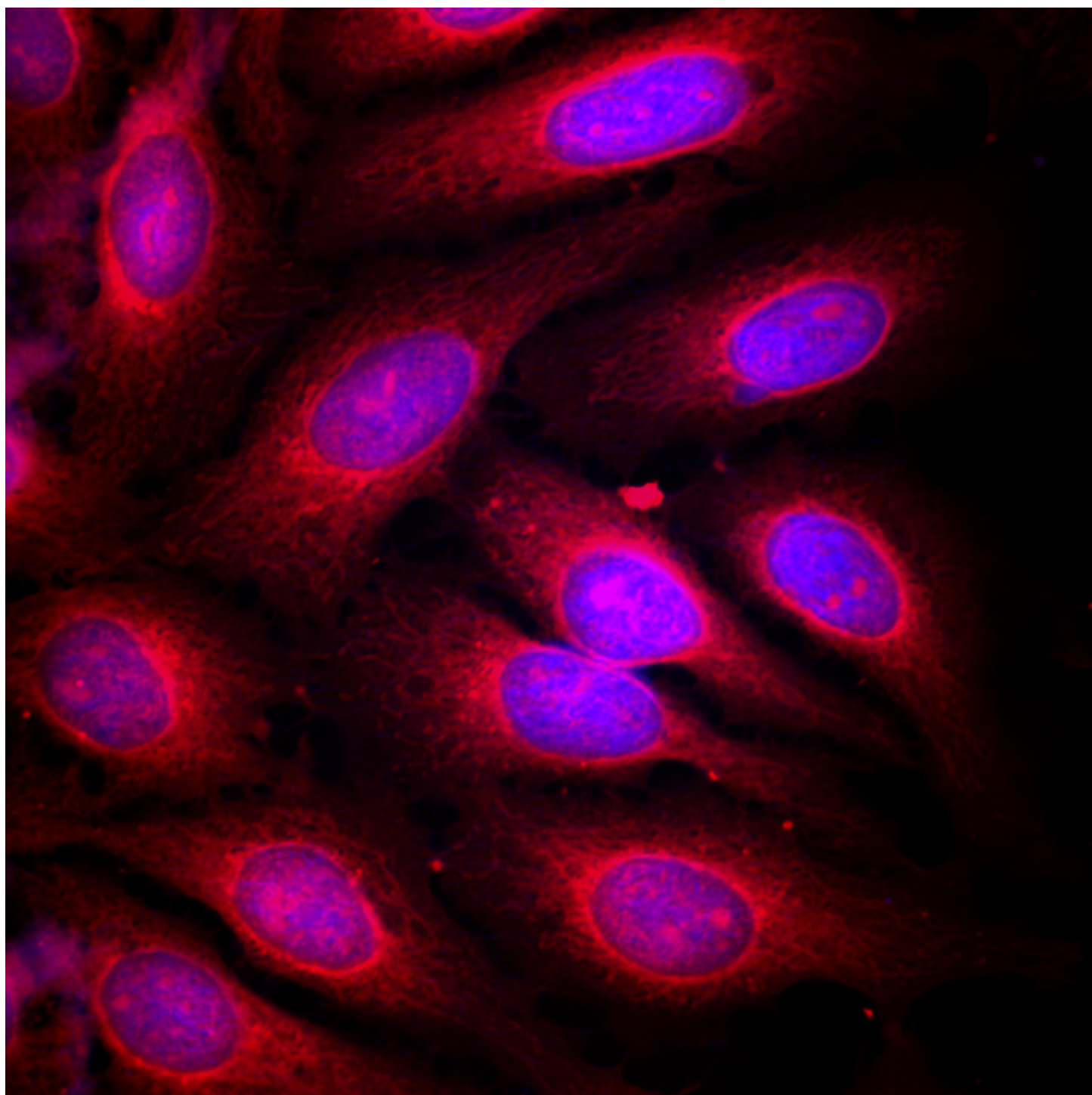

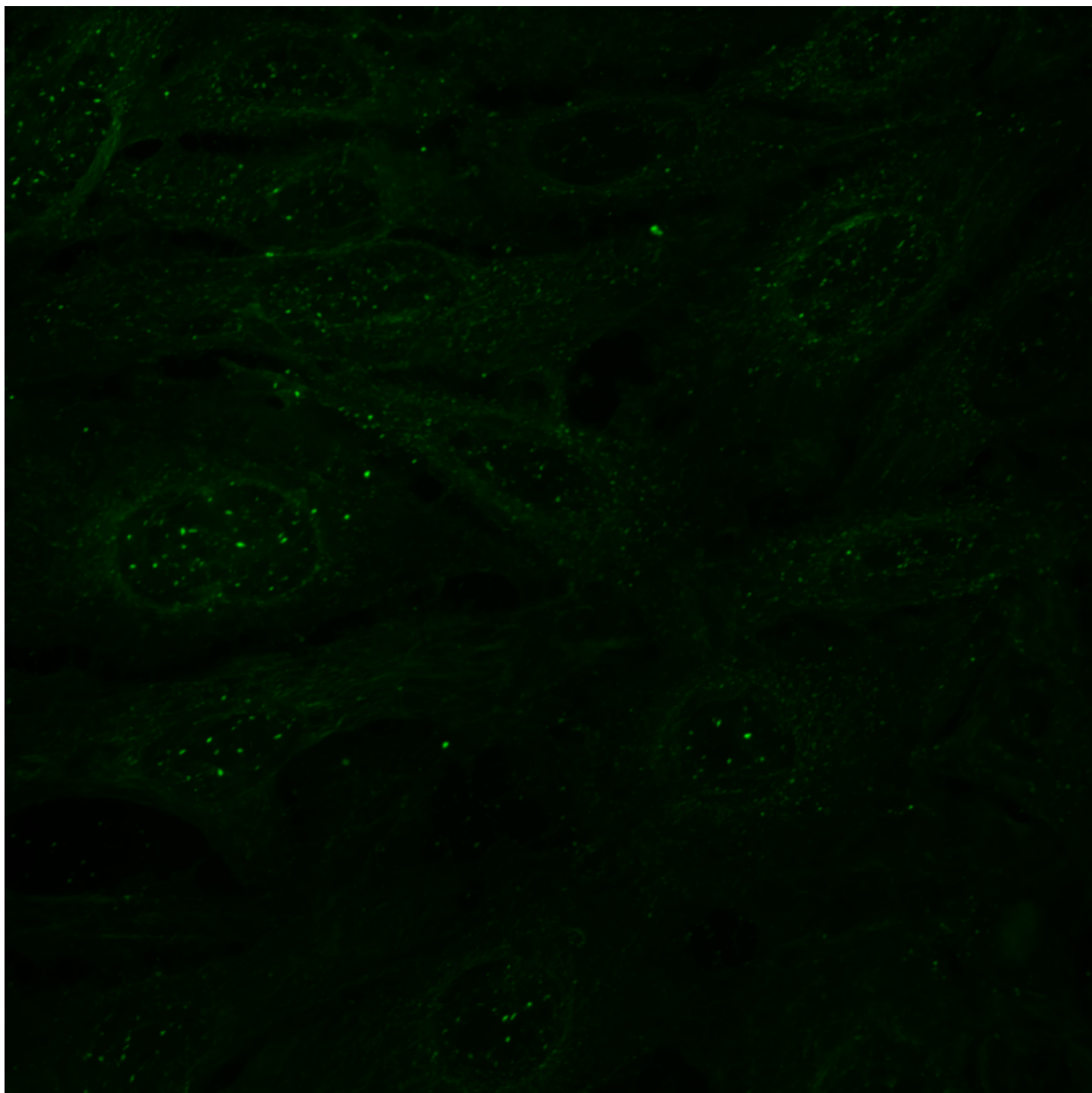

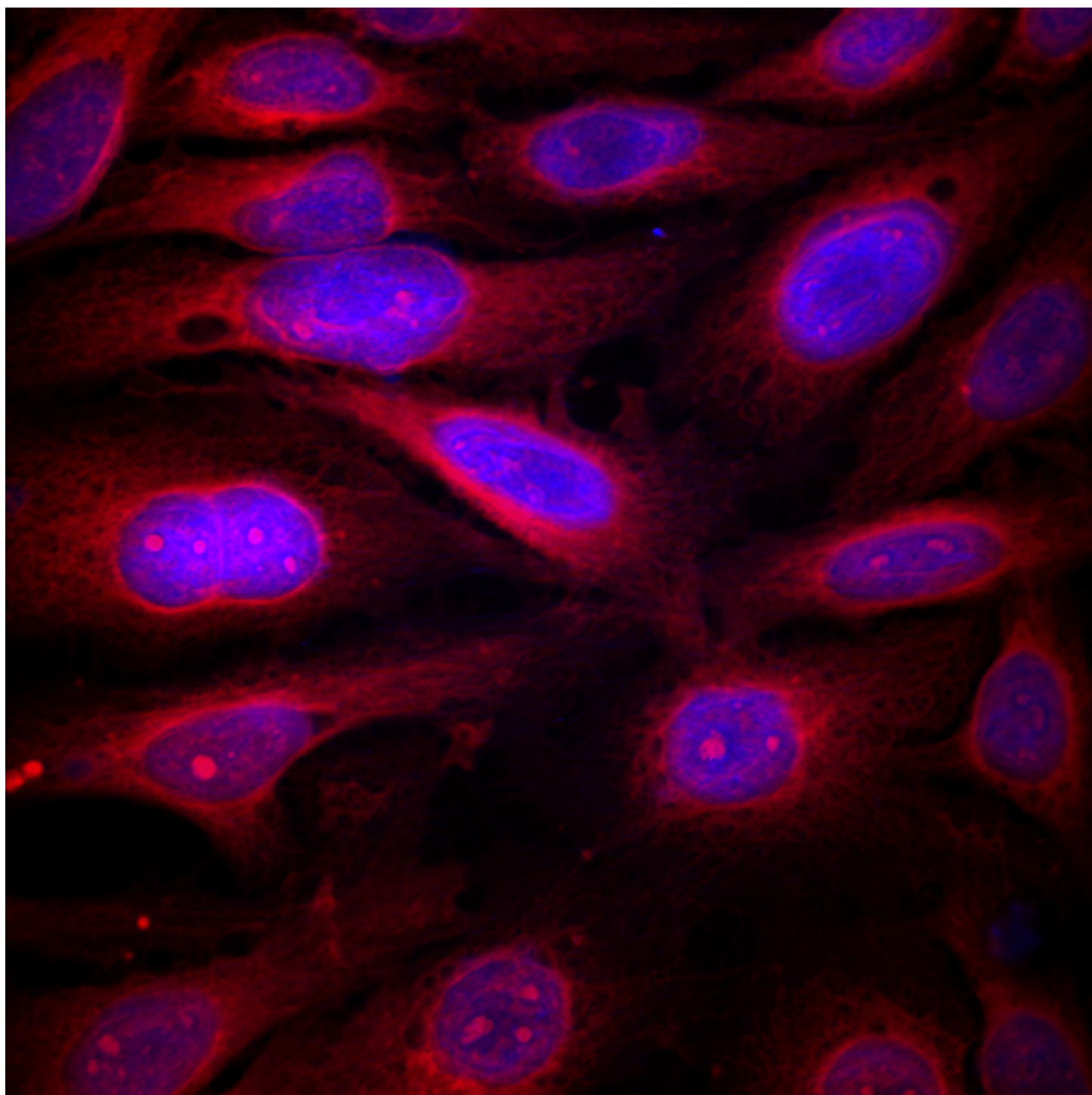

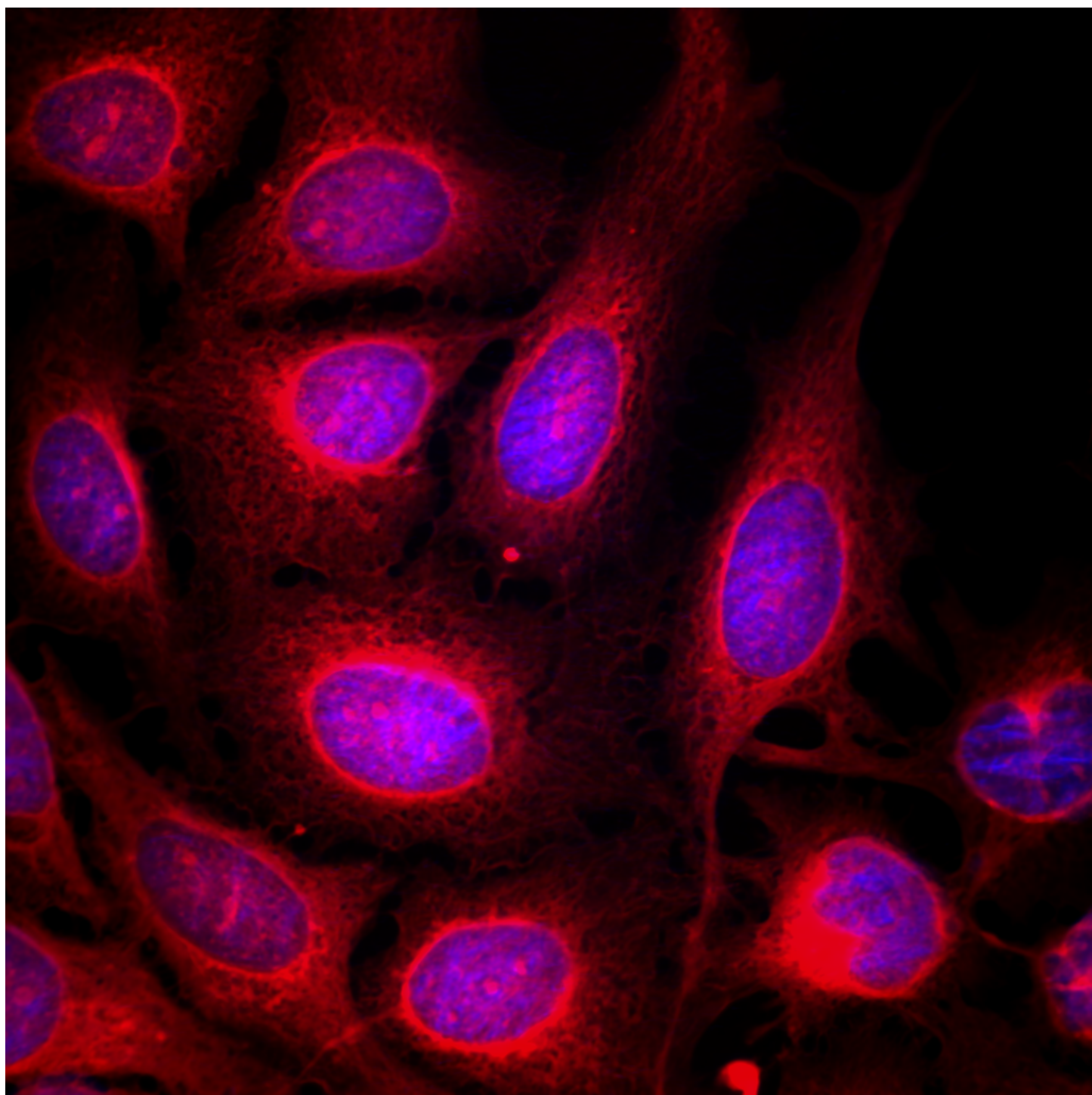

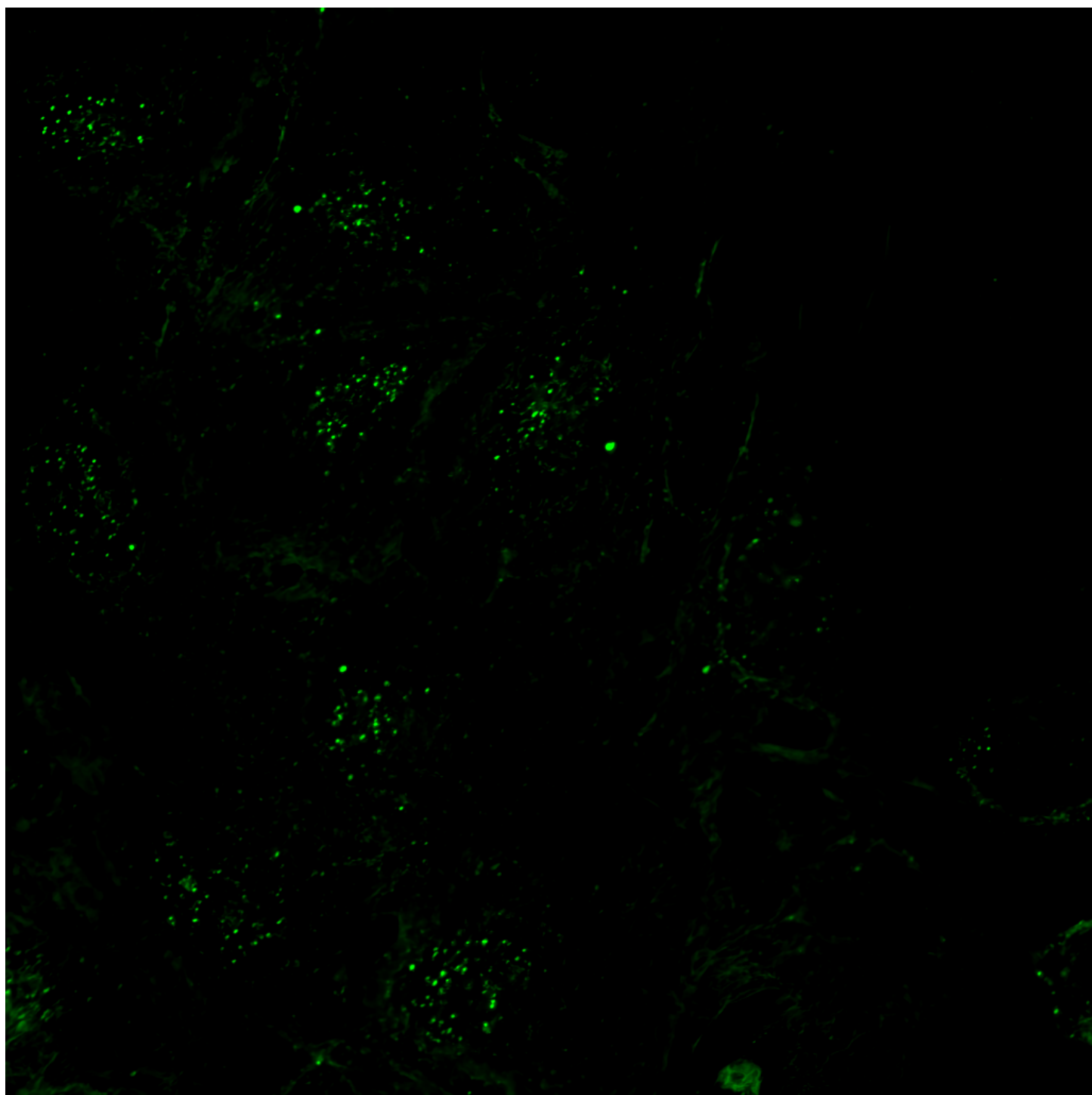

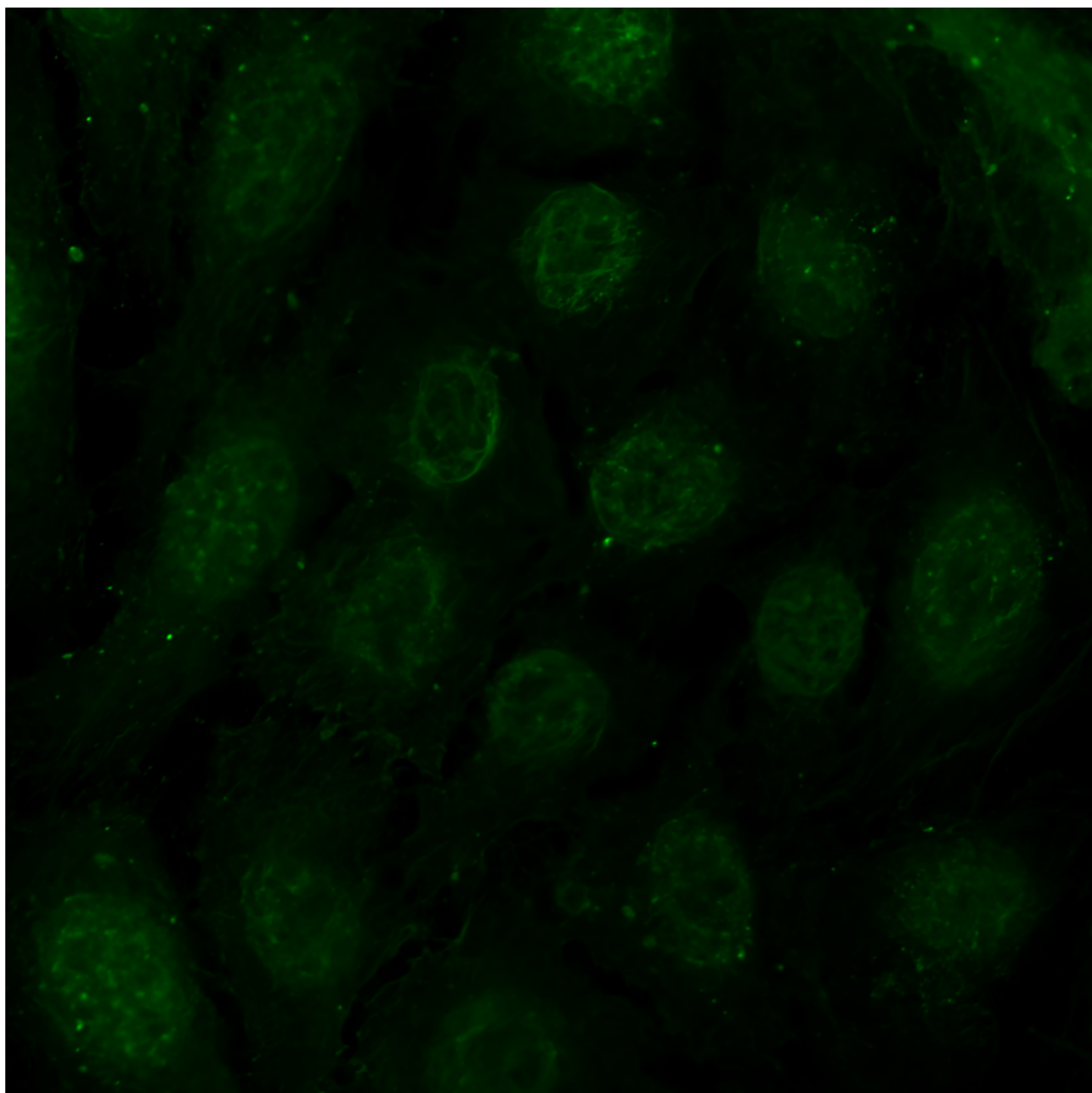

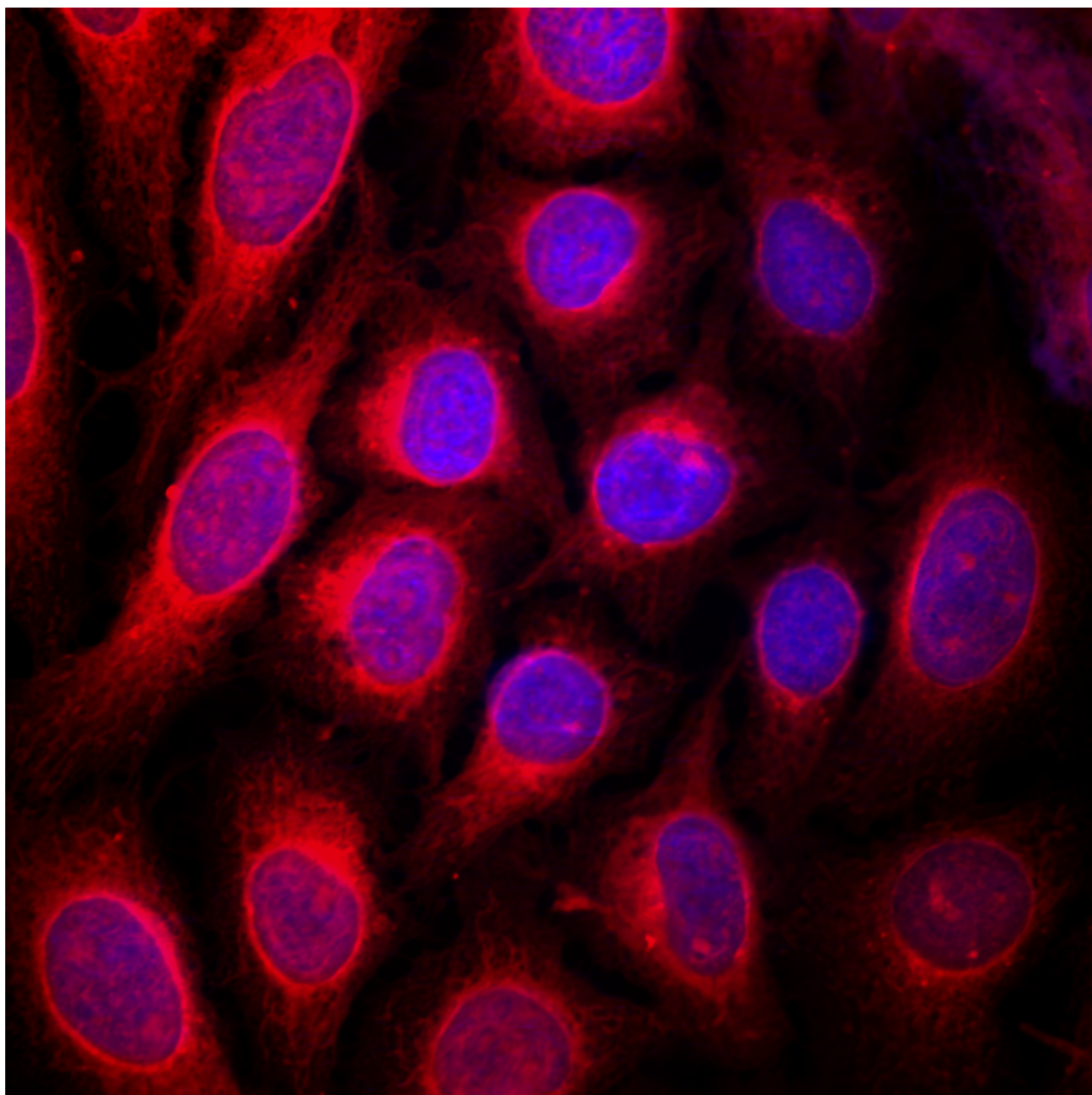

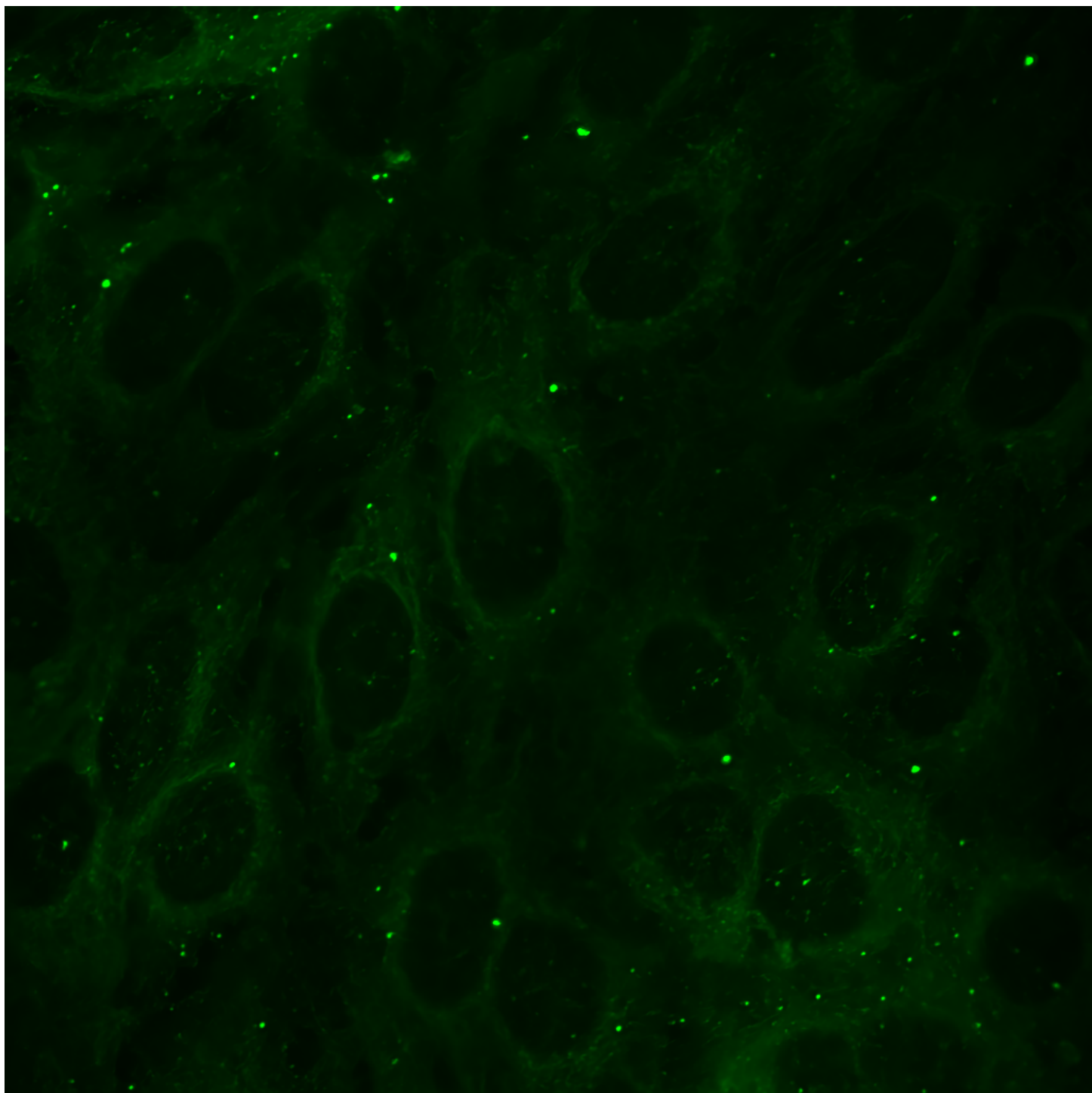

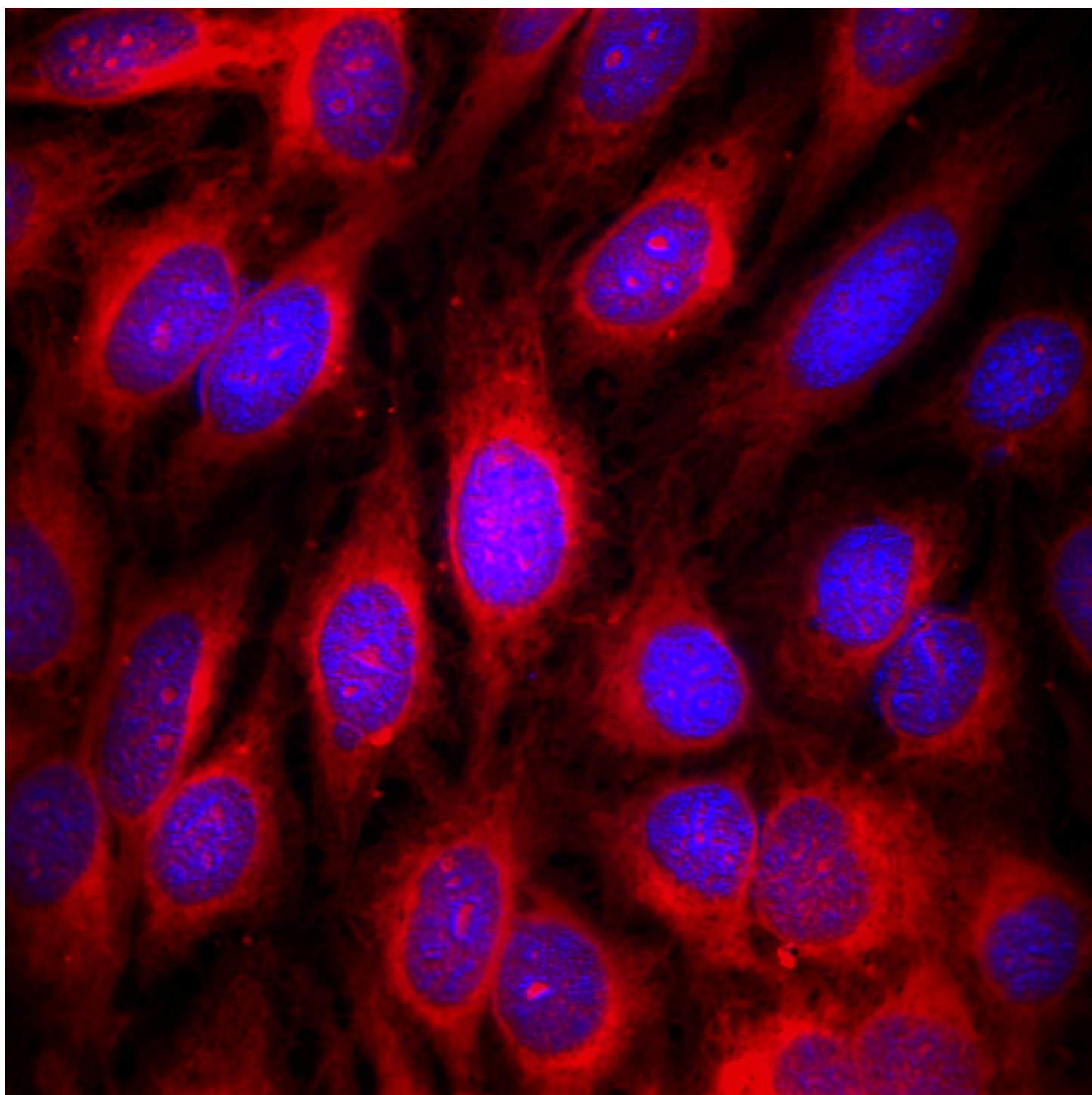

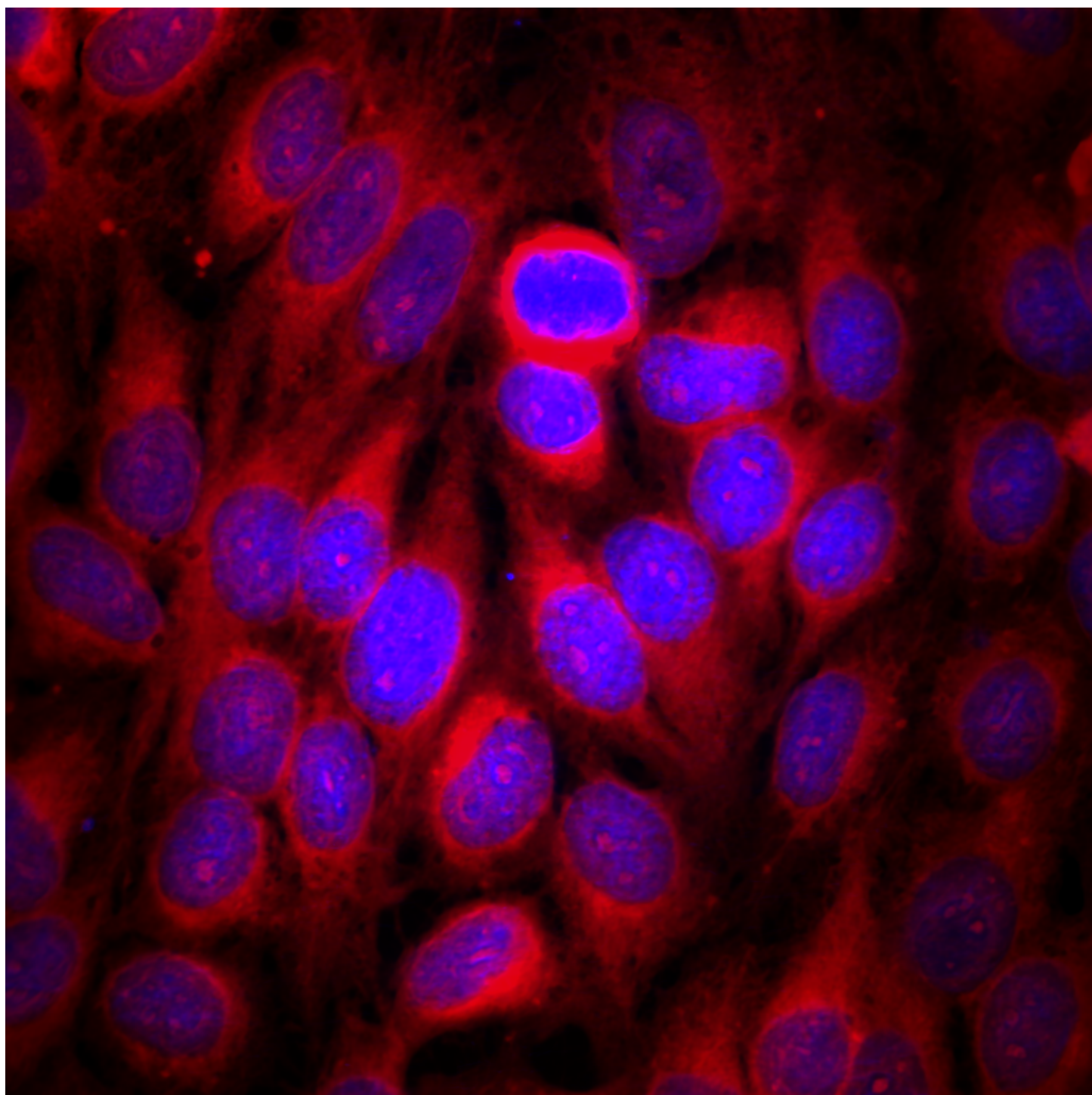

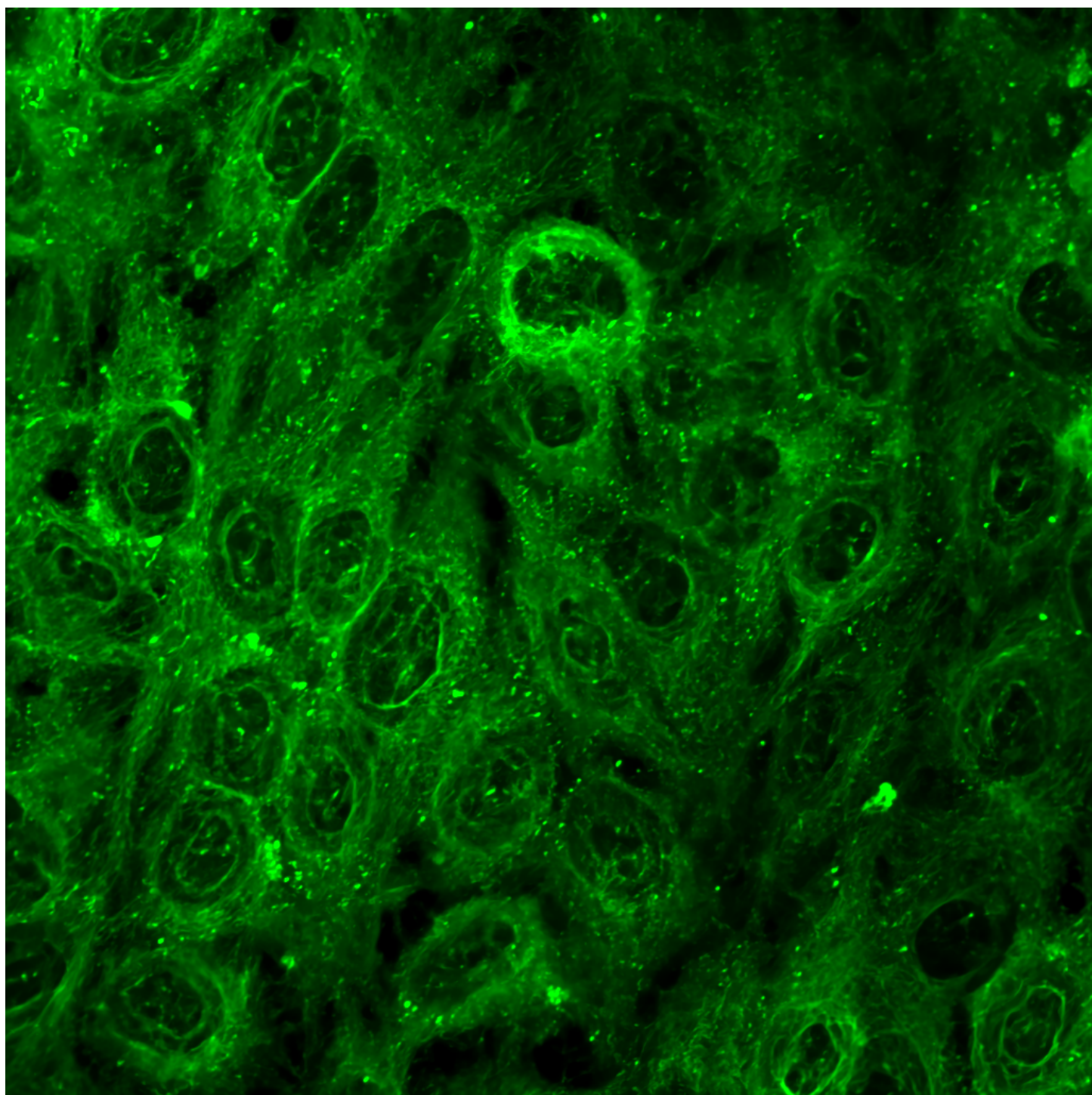

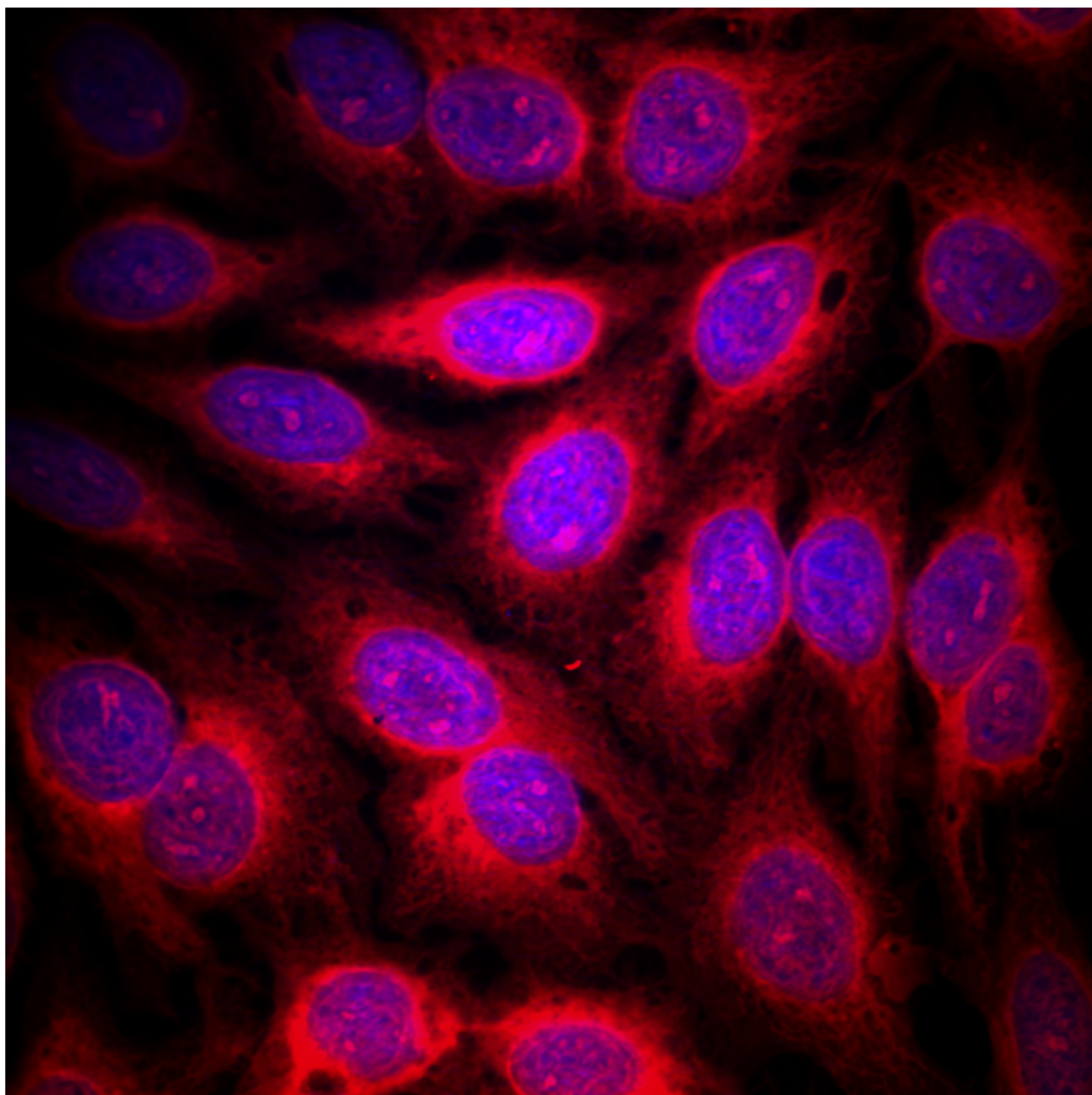

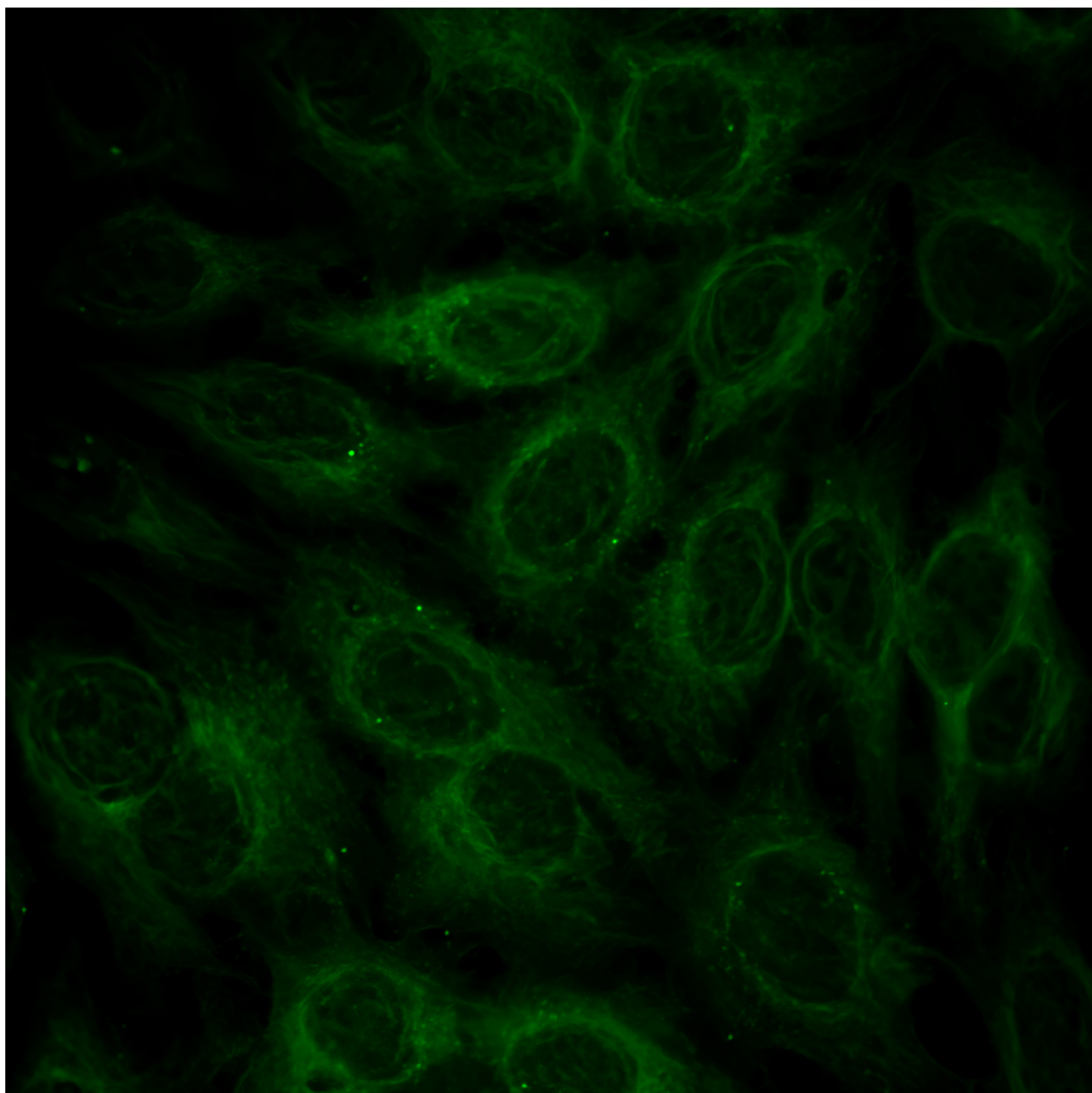

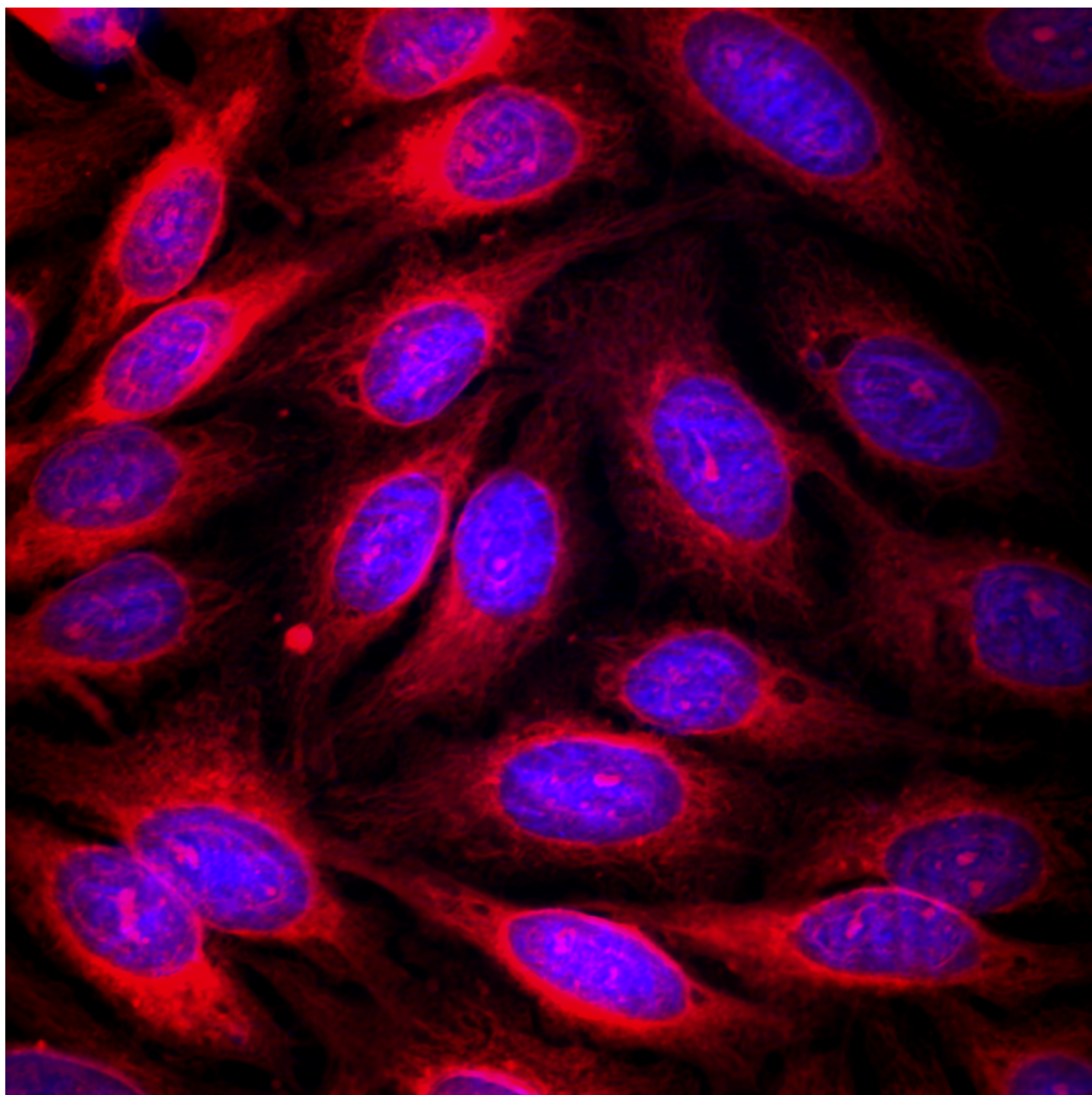

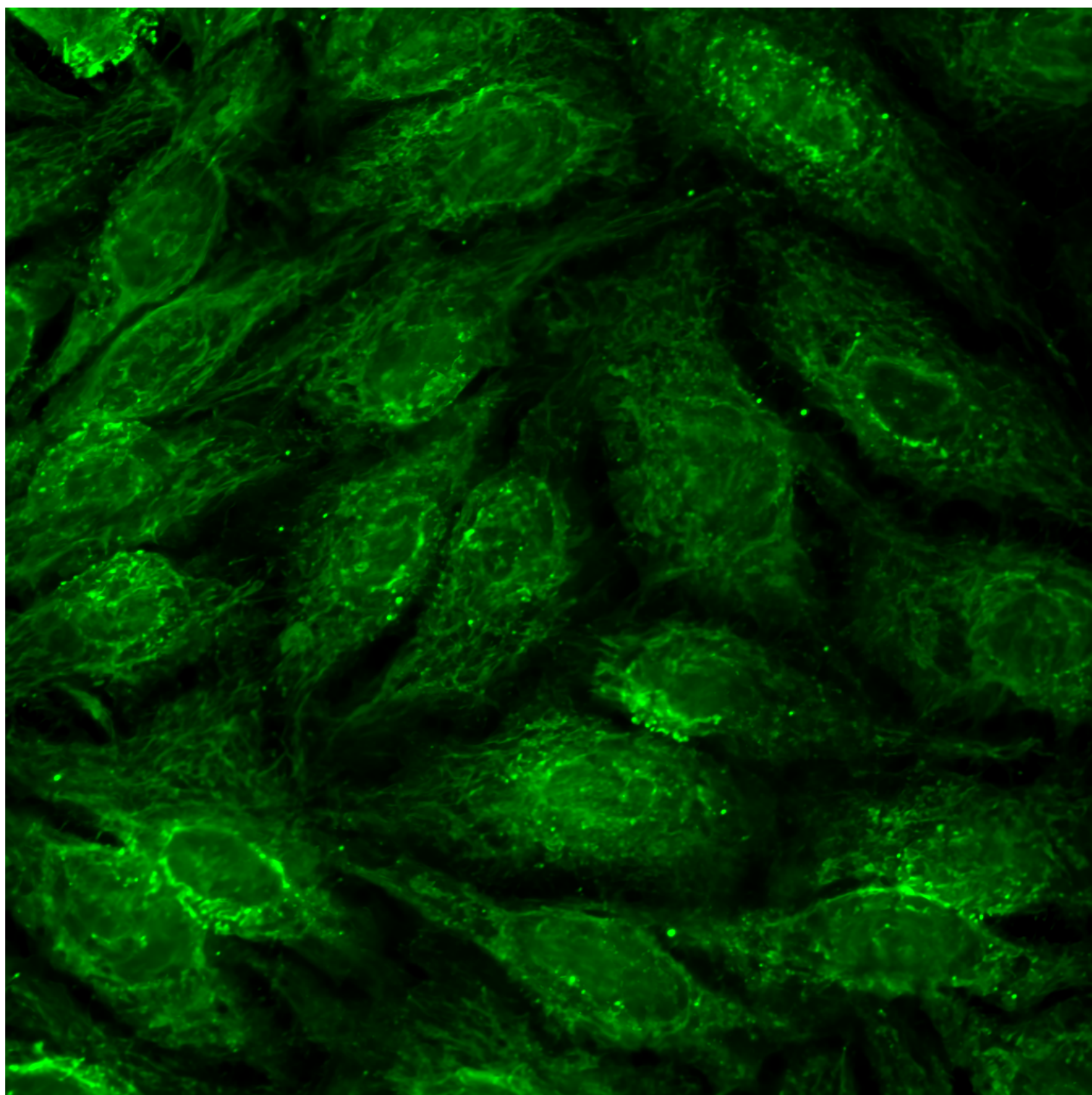

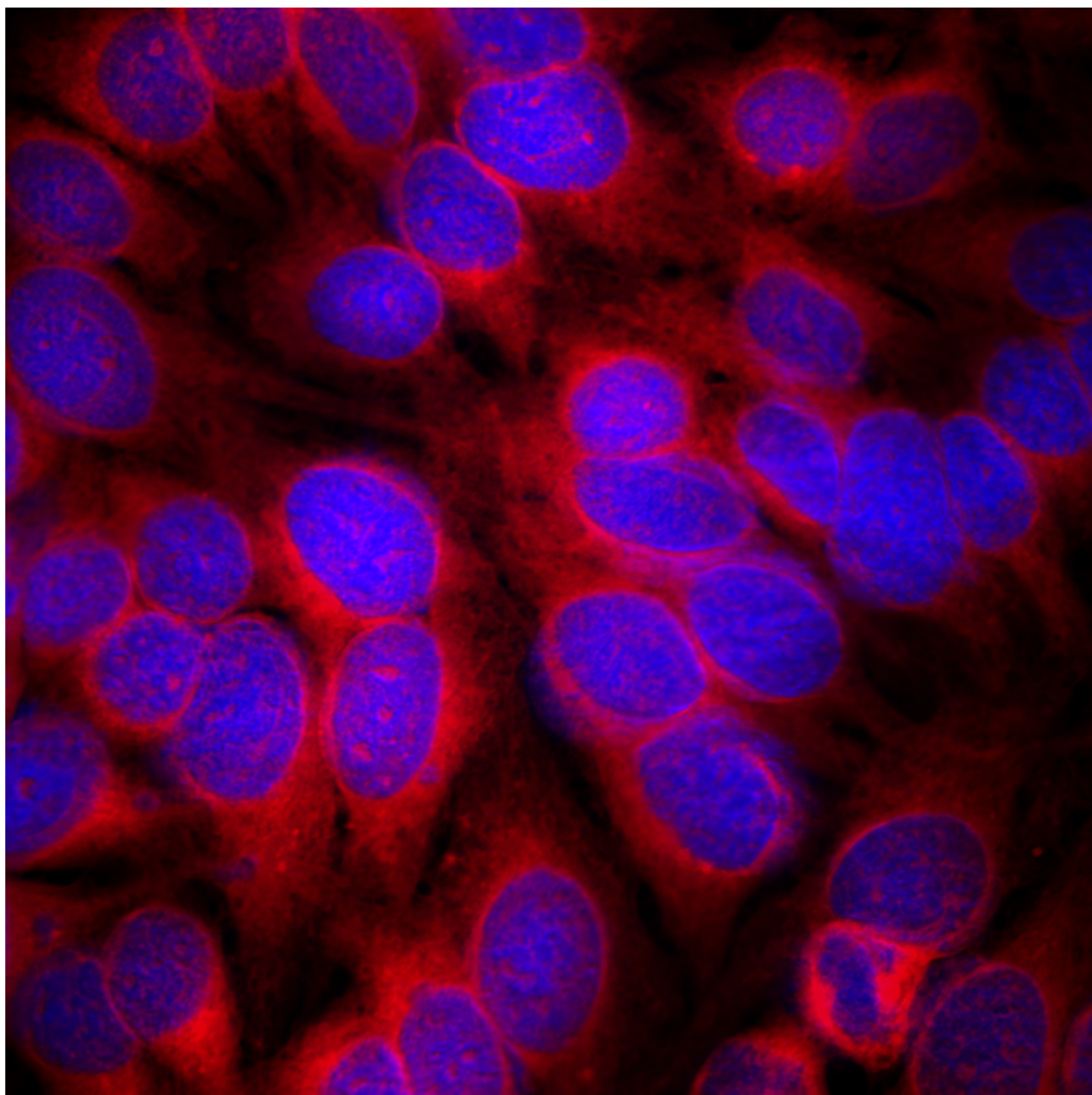

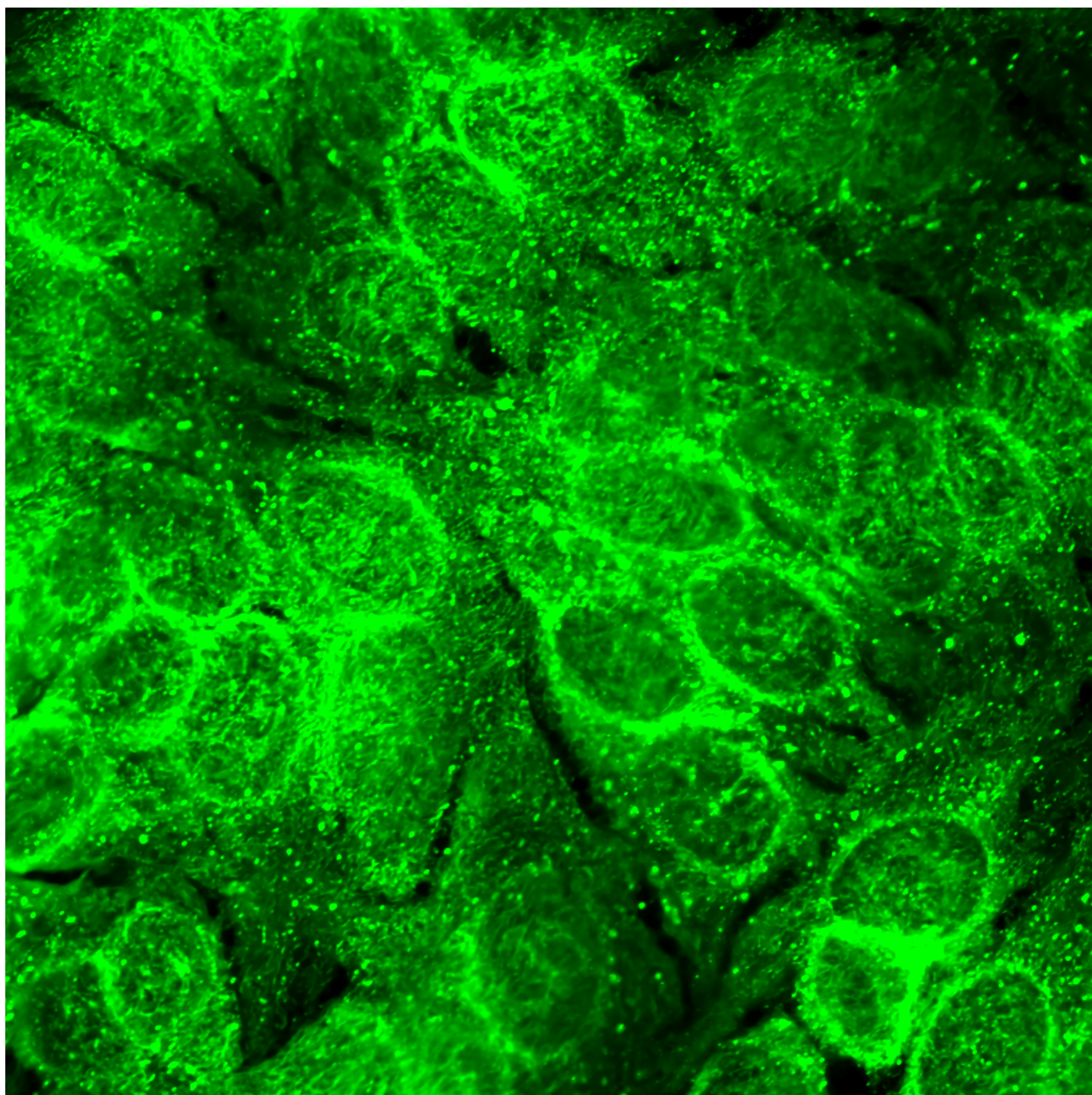

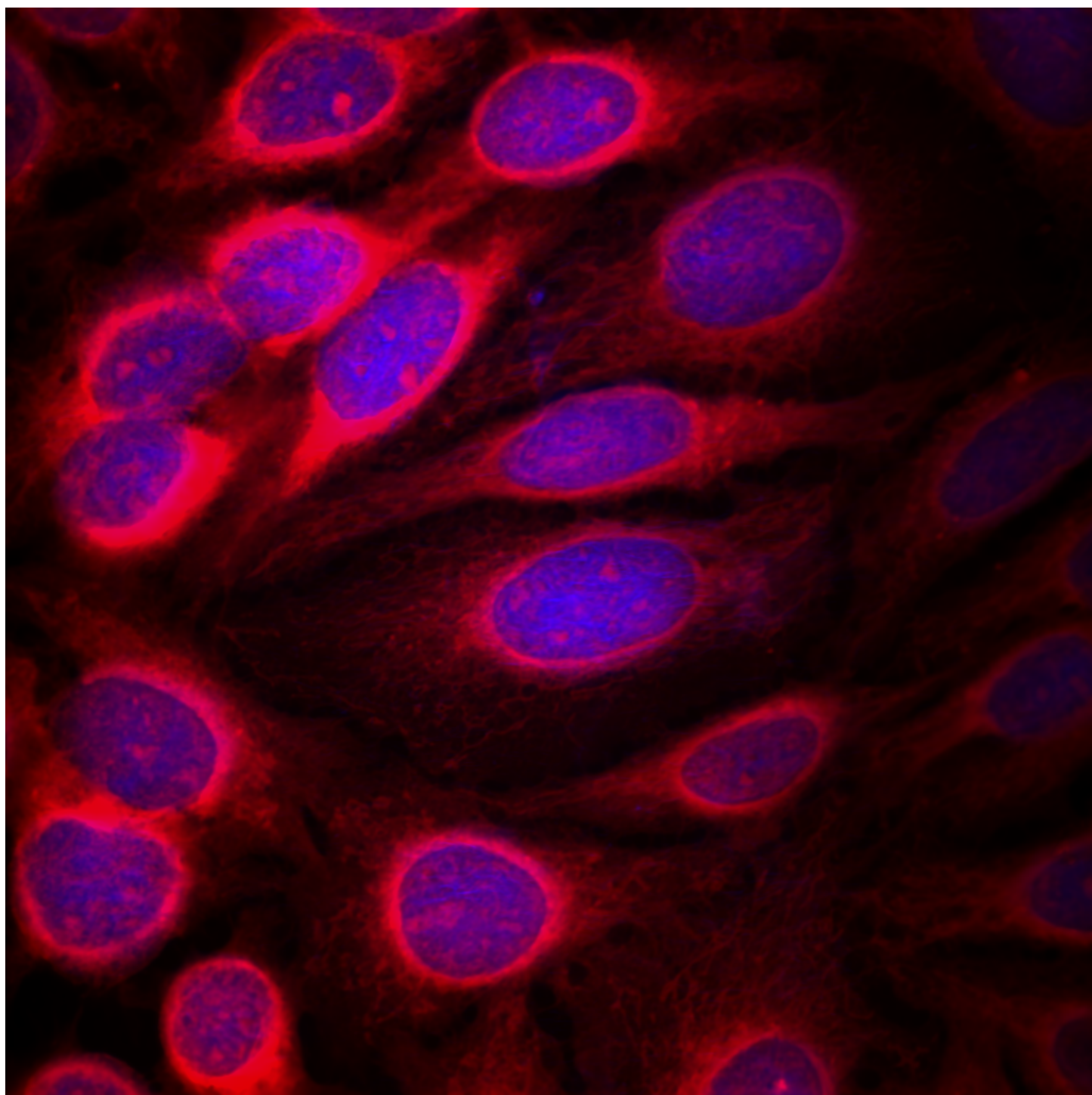

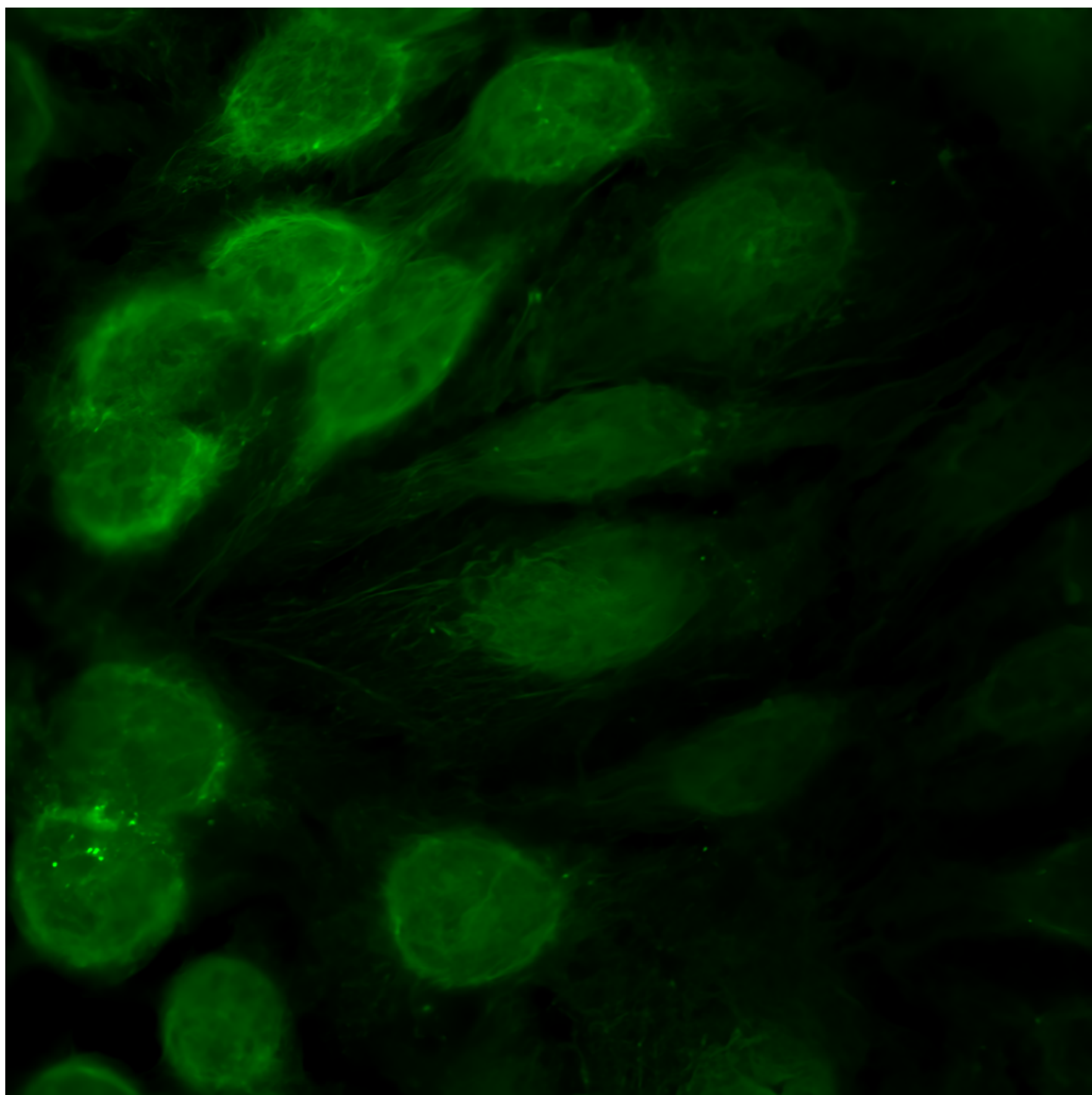

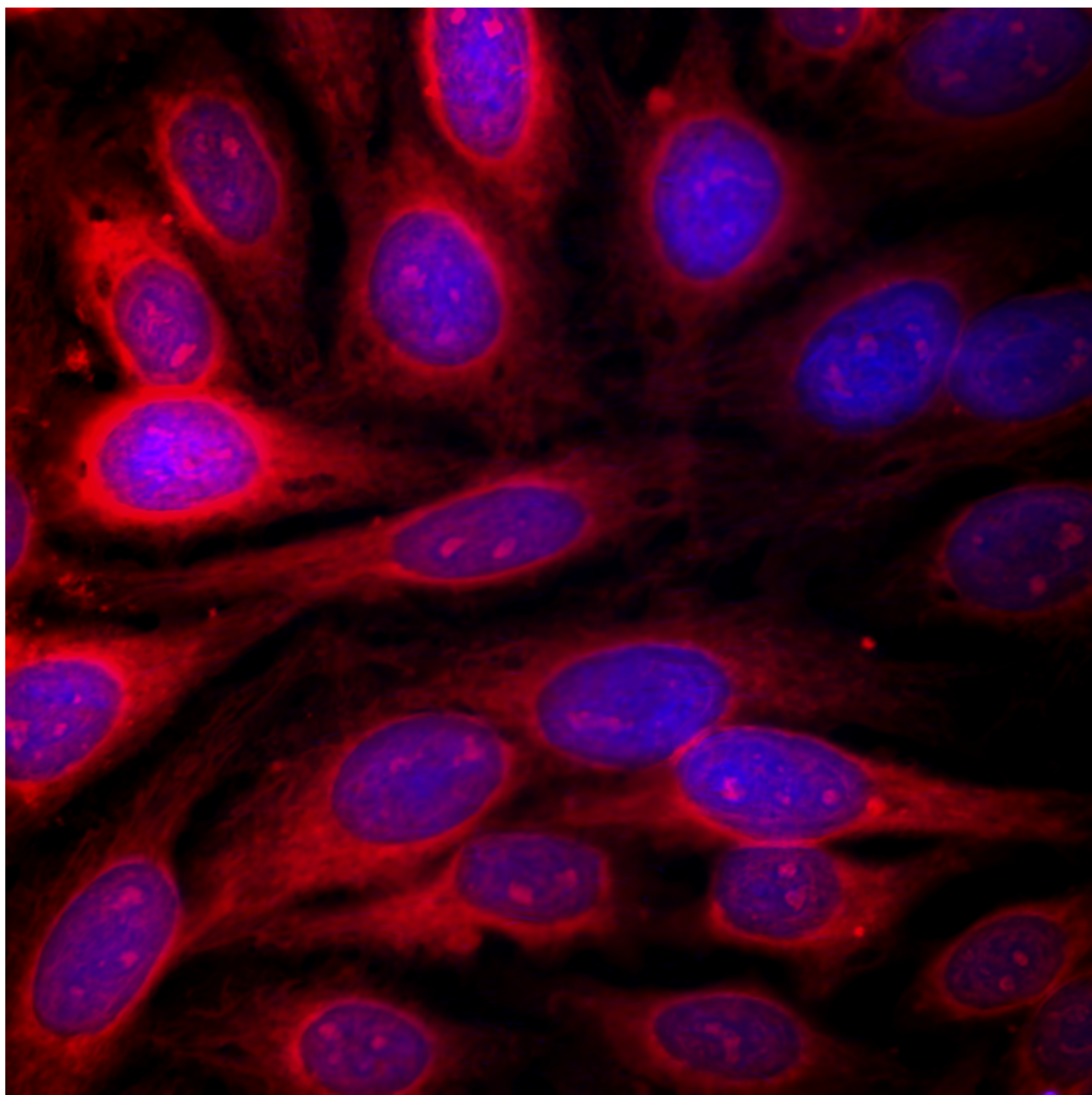

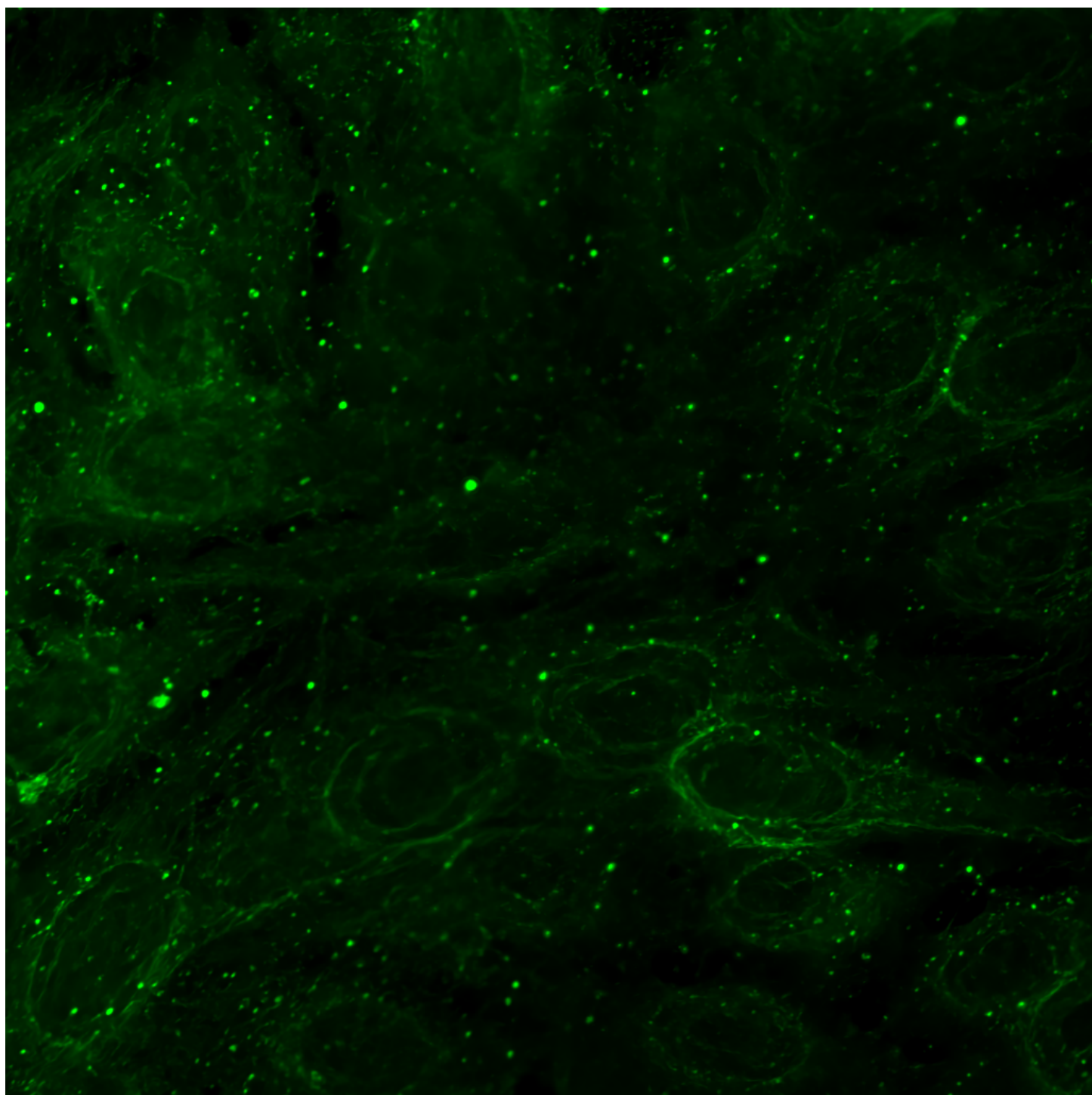

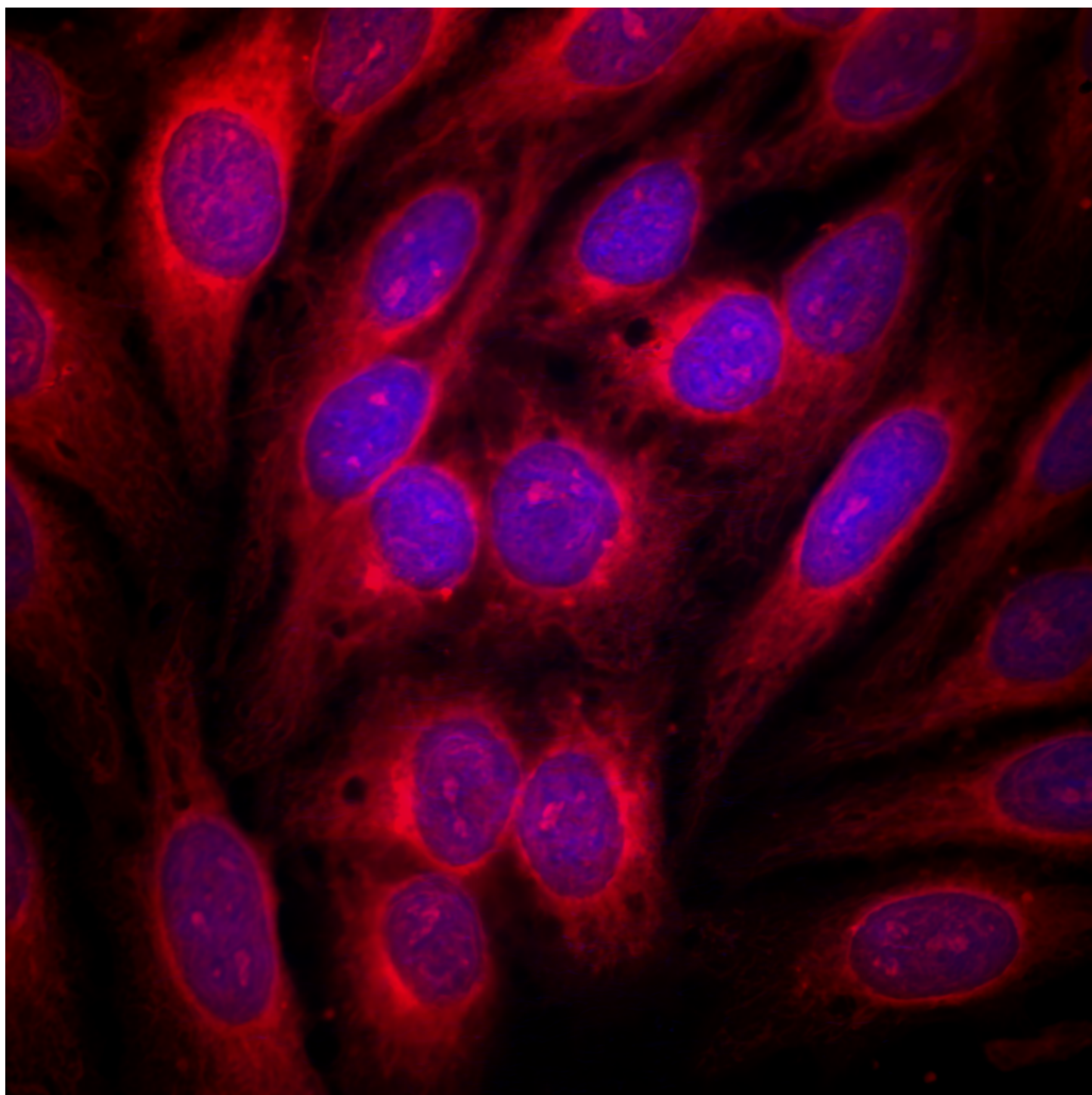

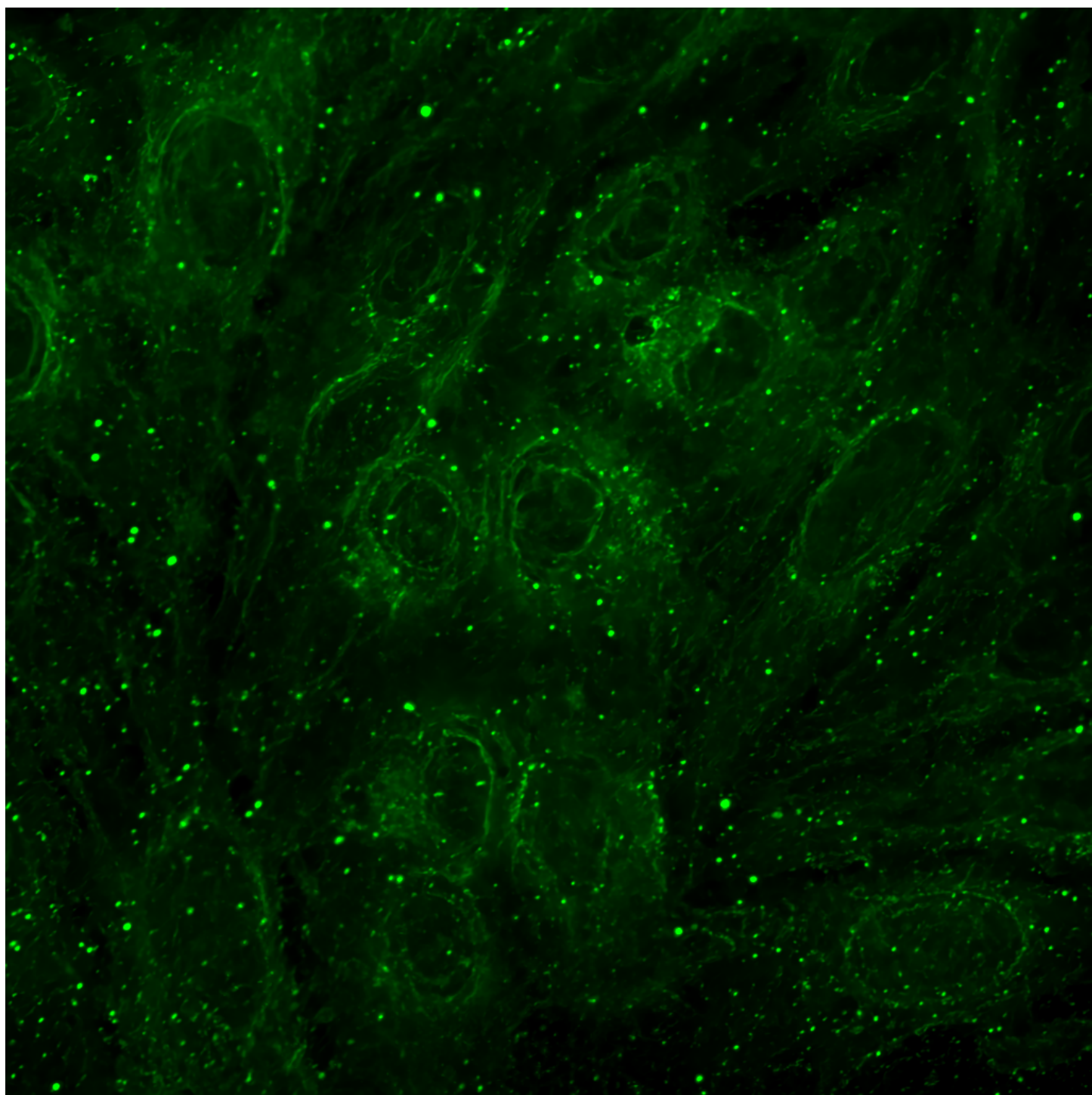

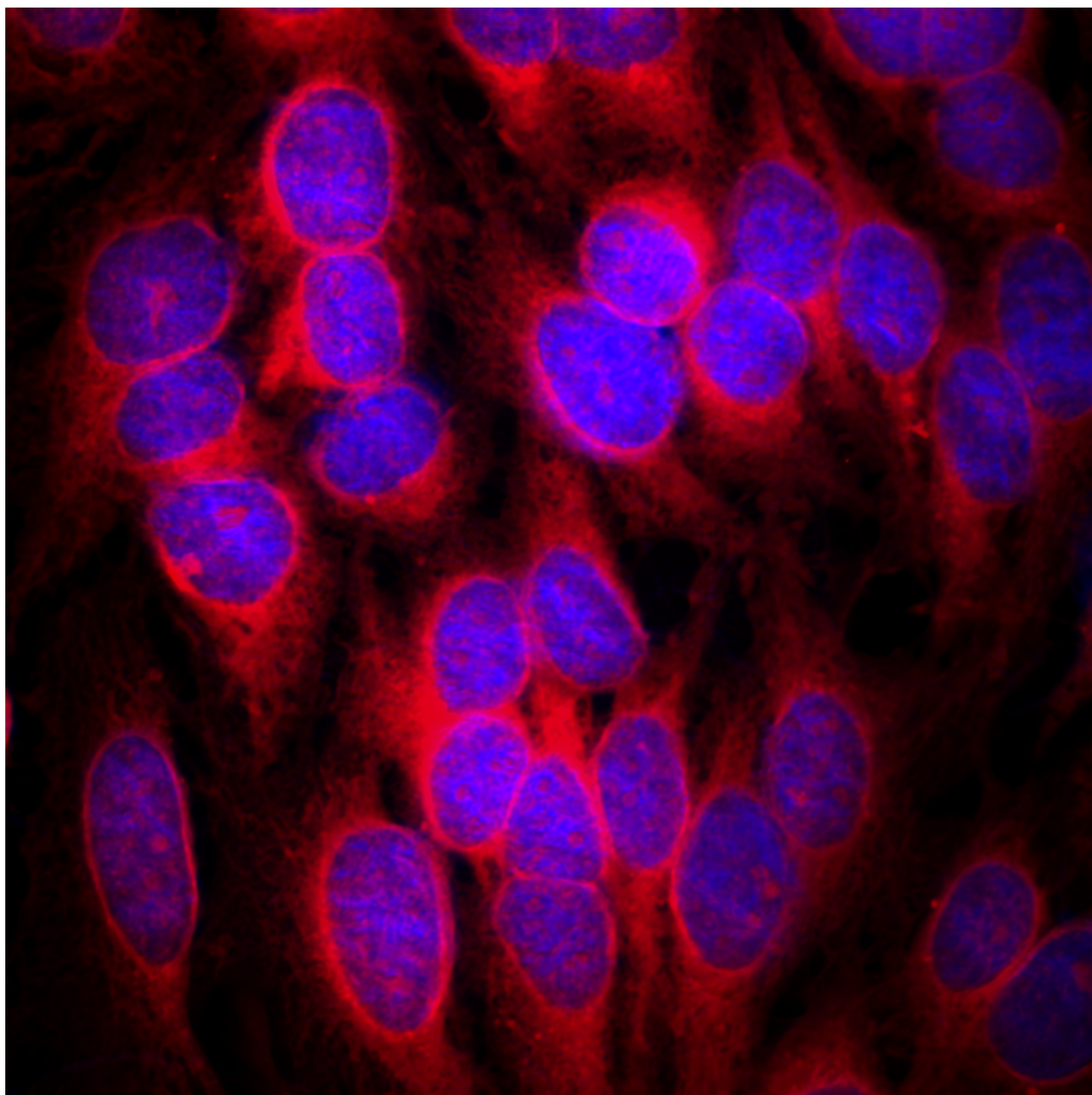

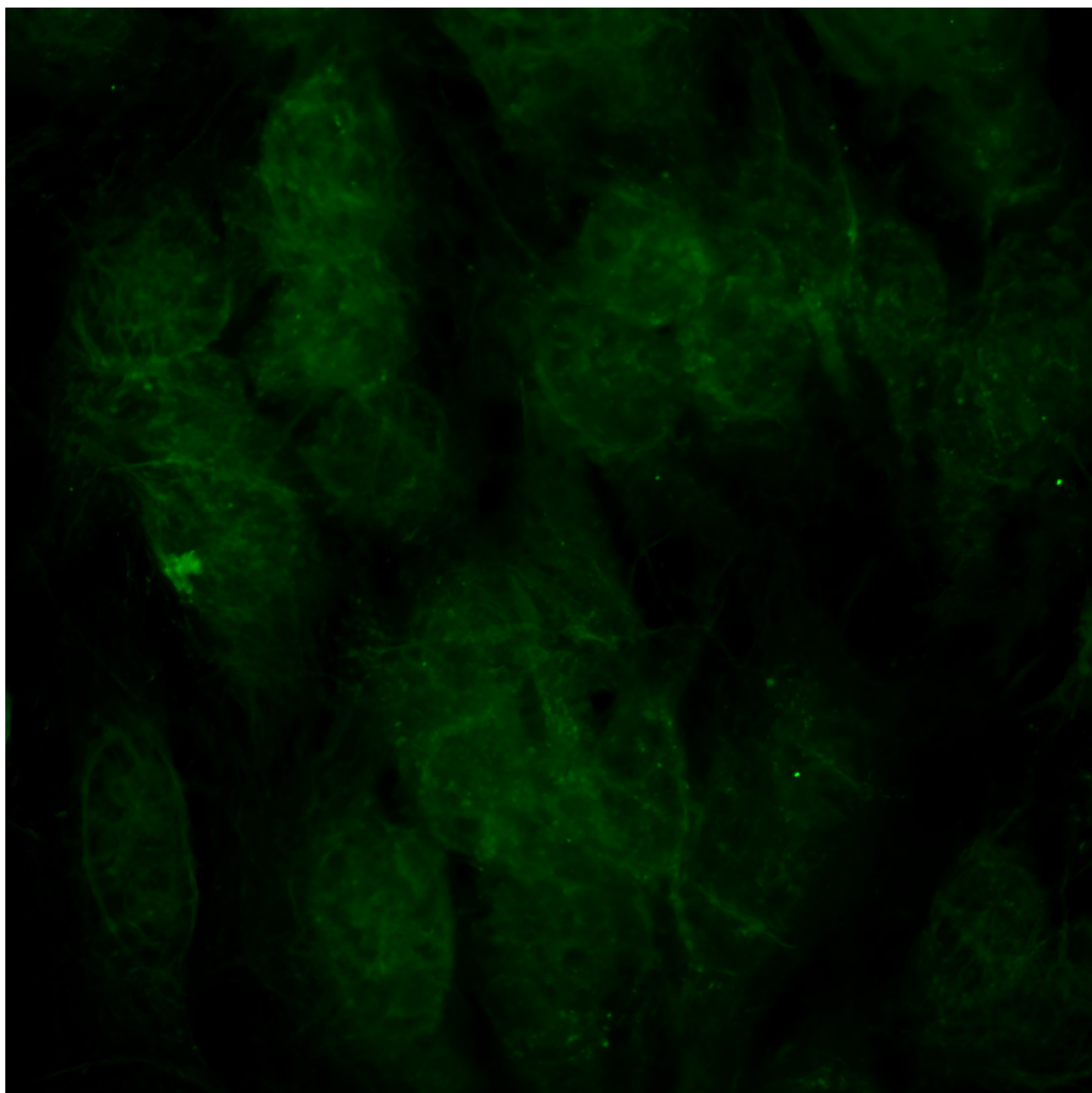

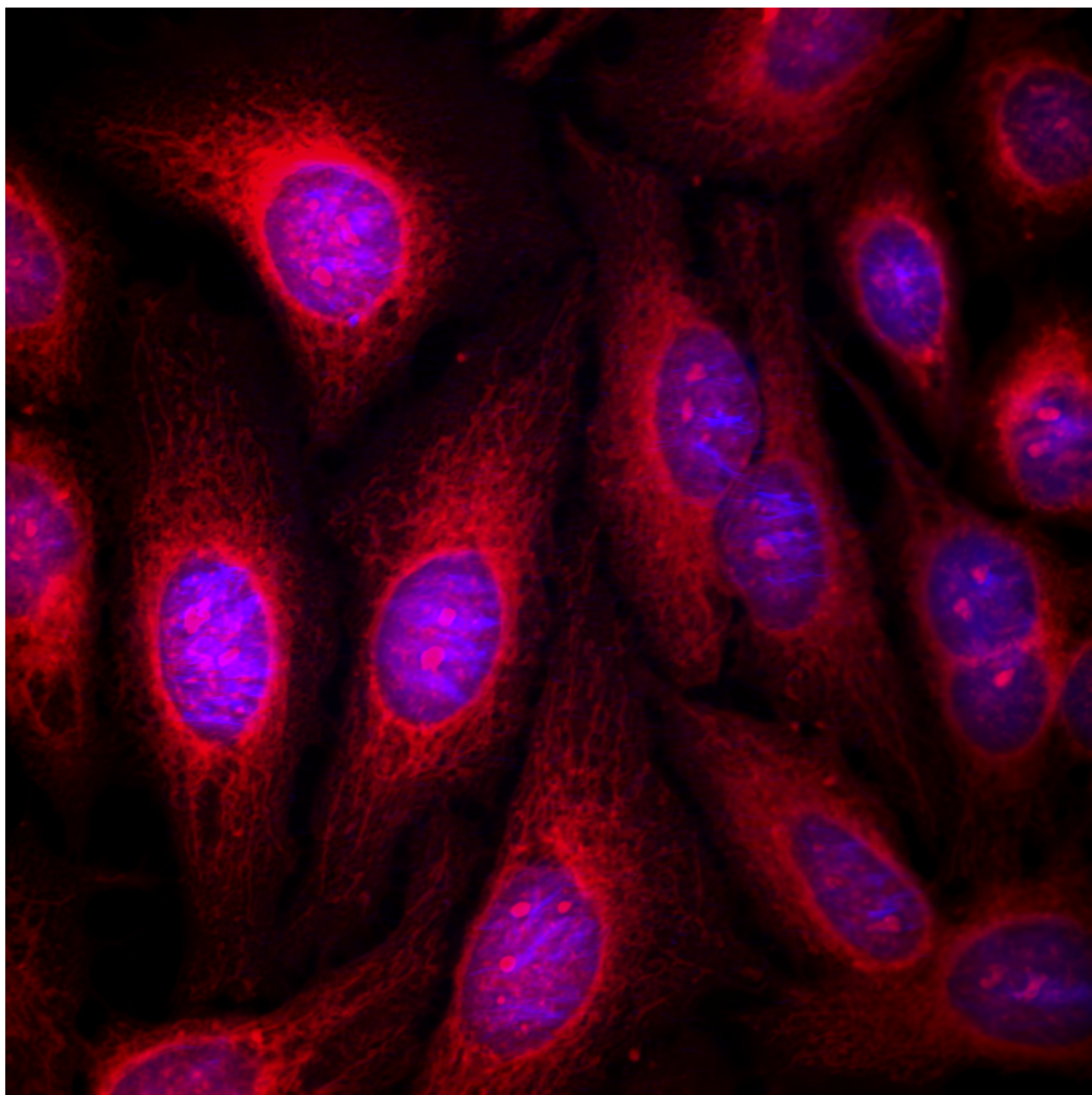

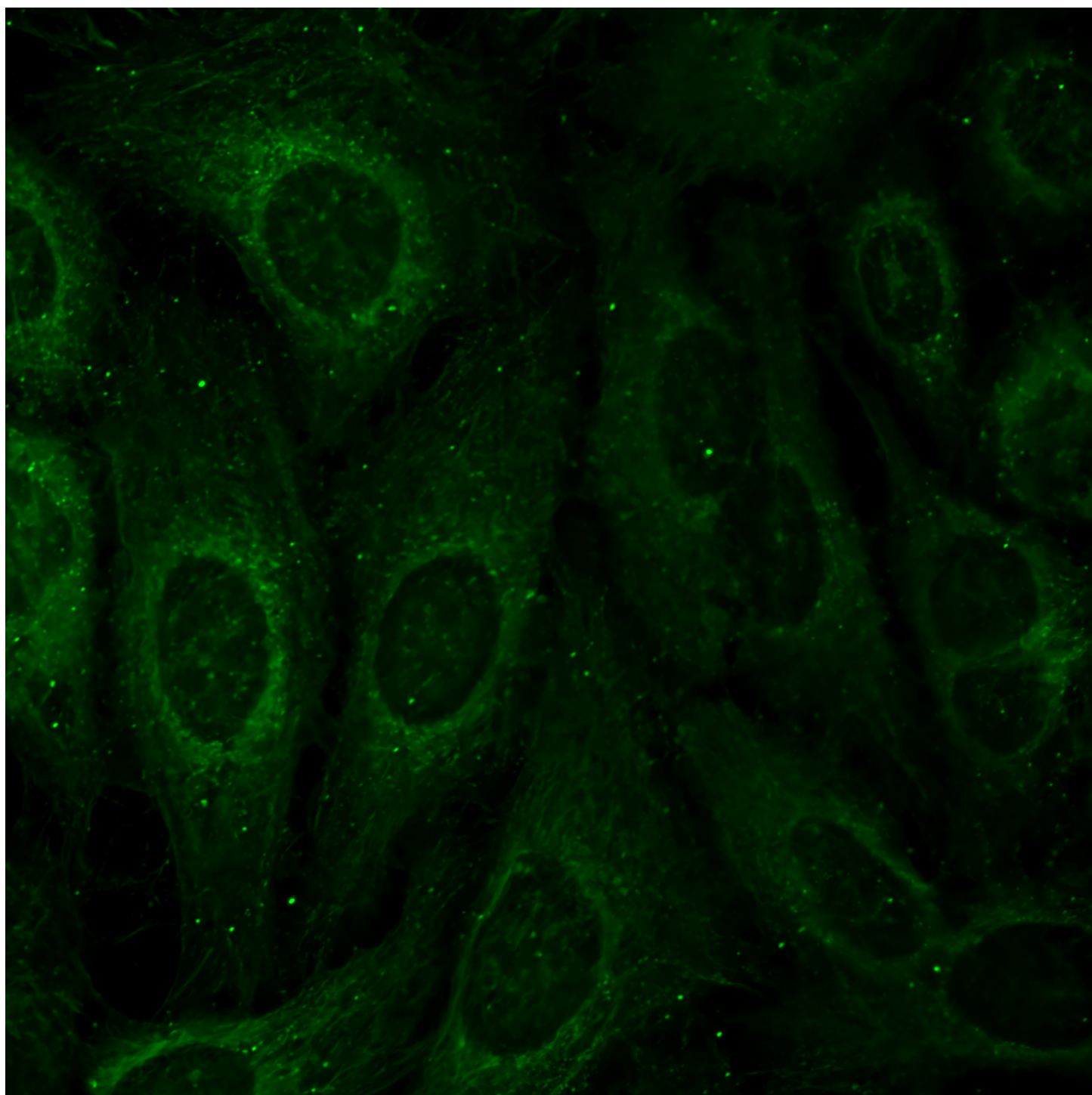

Supplement: Source Data Extended Data Fig. 2 — Image Source Data. [file 41590_2022_1271_MOESM15_ESM.pdf]
