# Supplementary material for: Partial RAG deficiency in humans induces dysregulated peripheral lymphocyte development and humoral tolerance defect with accumulation of T-bet+ B cells
Source: Nat Immunol. 2022 Jul 28;23(8):1256–72. doi: 10.1038/s41590-022-01271-6 (PMC9355881; doi:10.1038/s41590-022-01271-6)
Supplement: Source Data Extended Data Fig. 5 — Image Source Data. [file 41590_2022_1271_MOESM18_ESM.pdf]

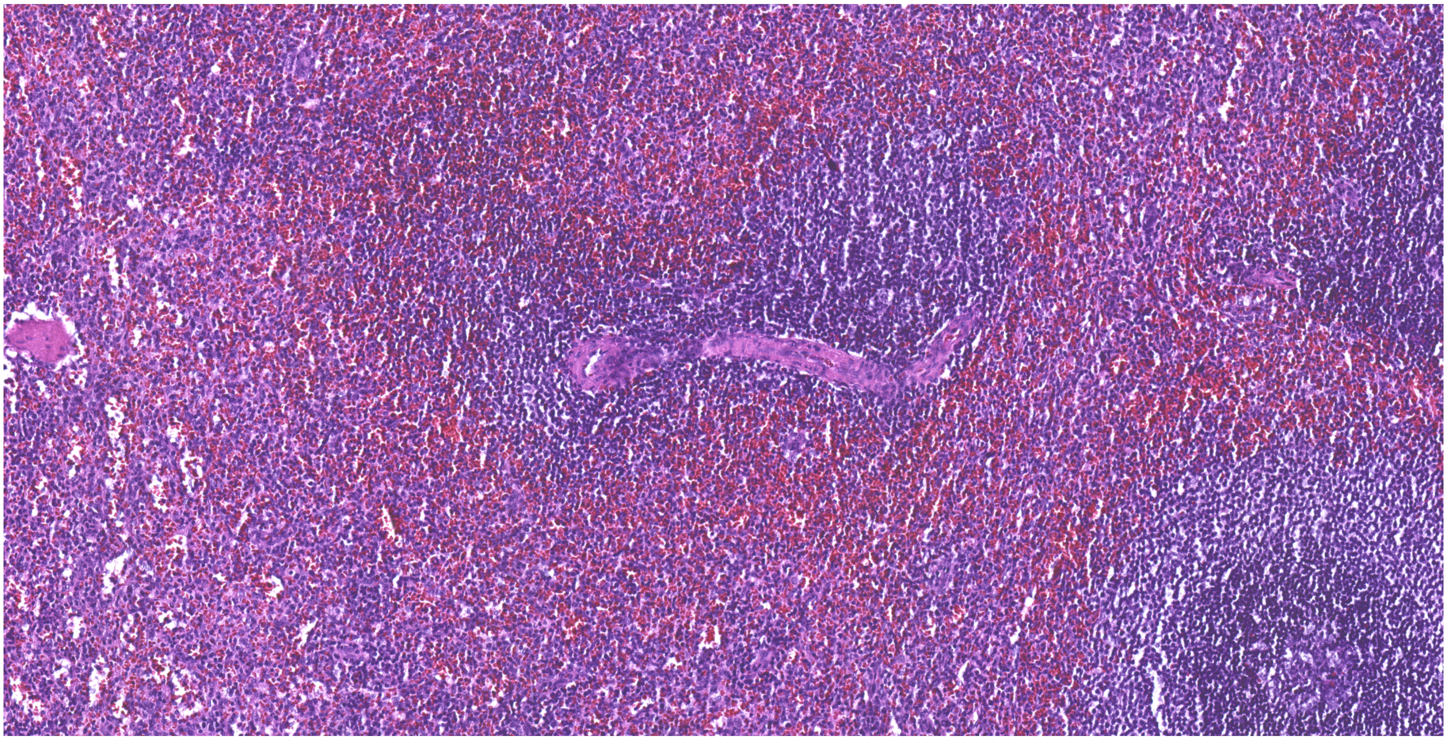

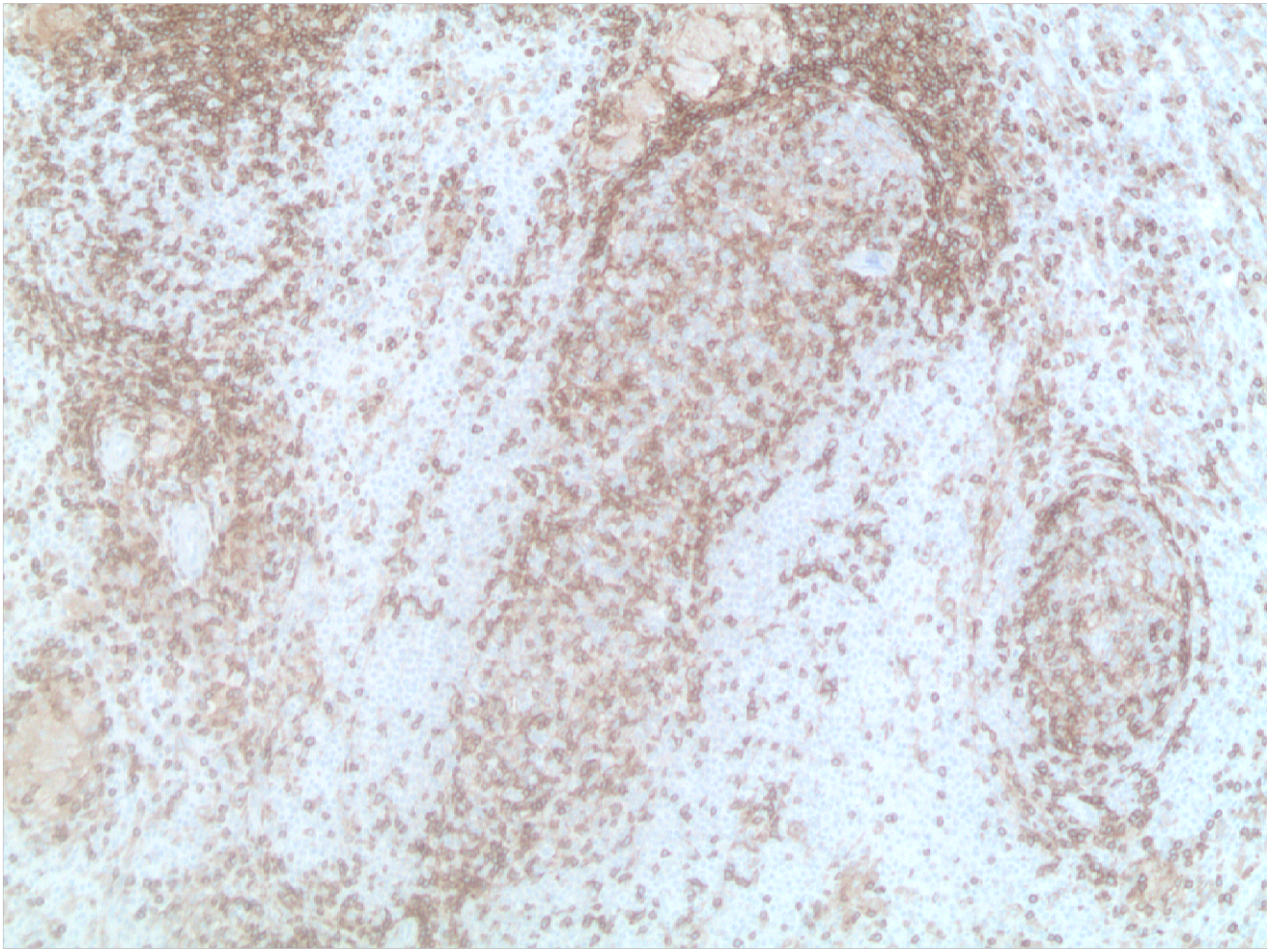

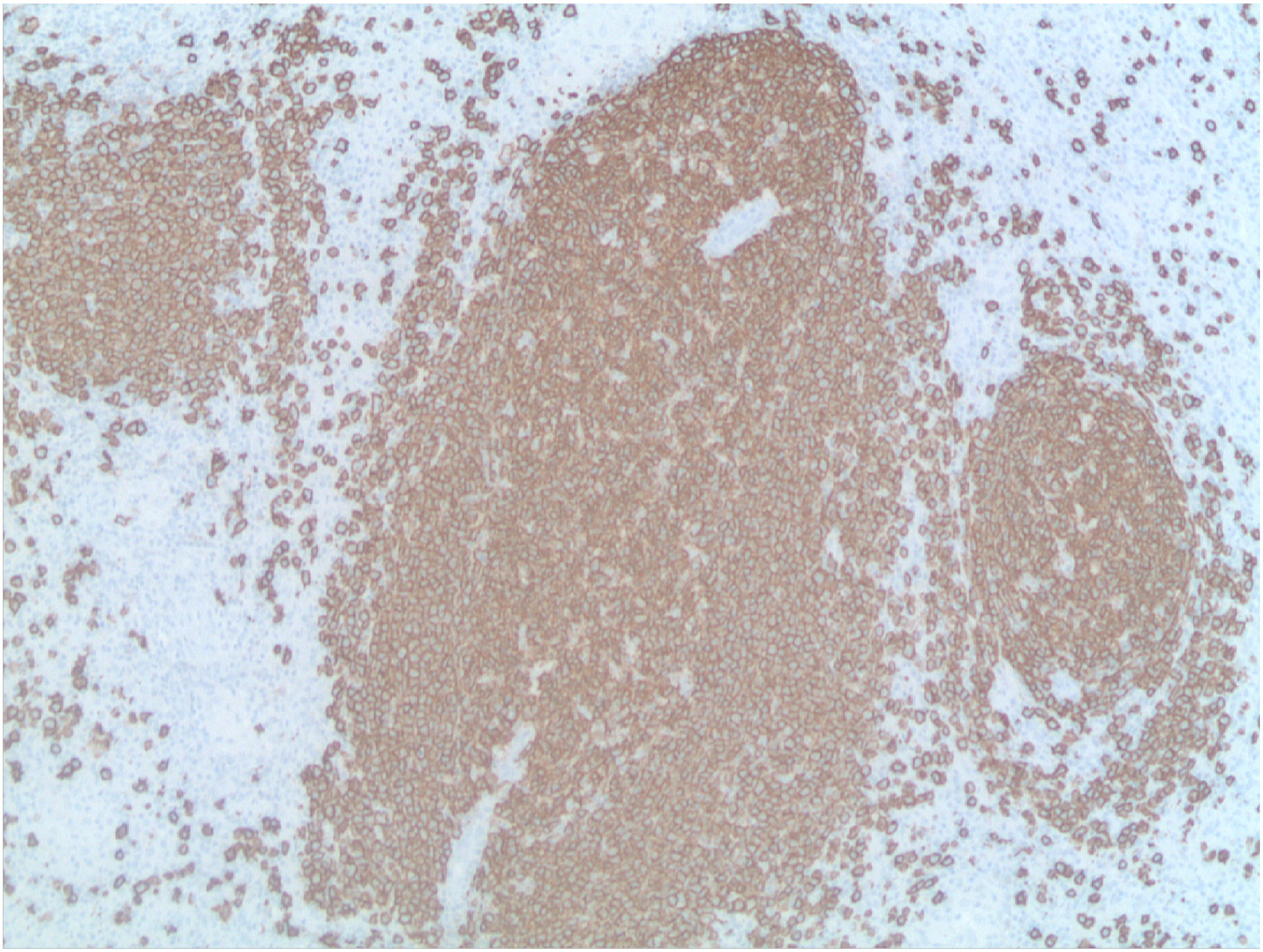

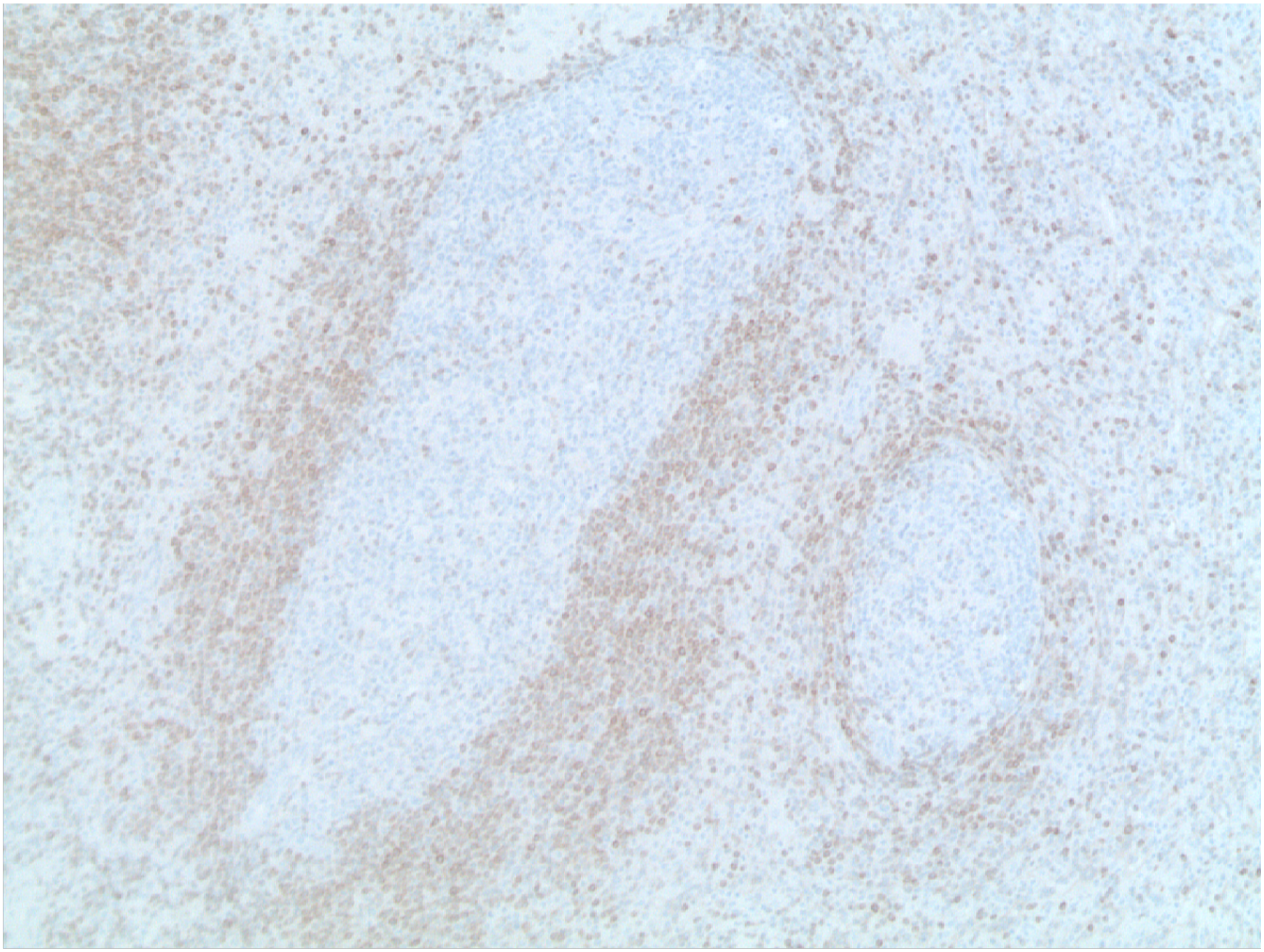

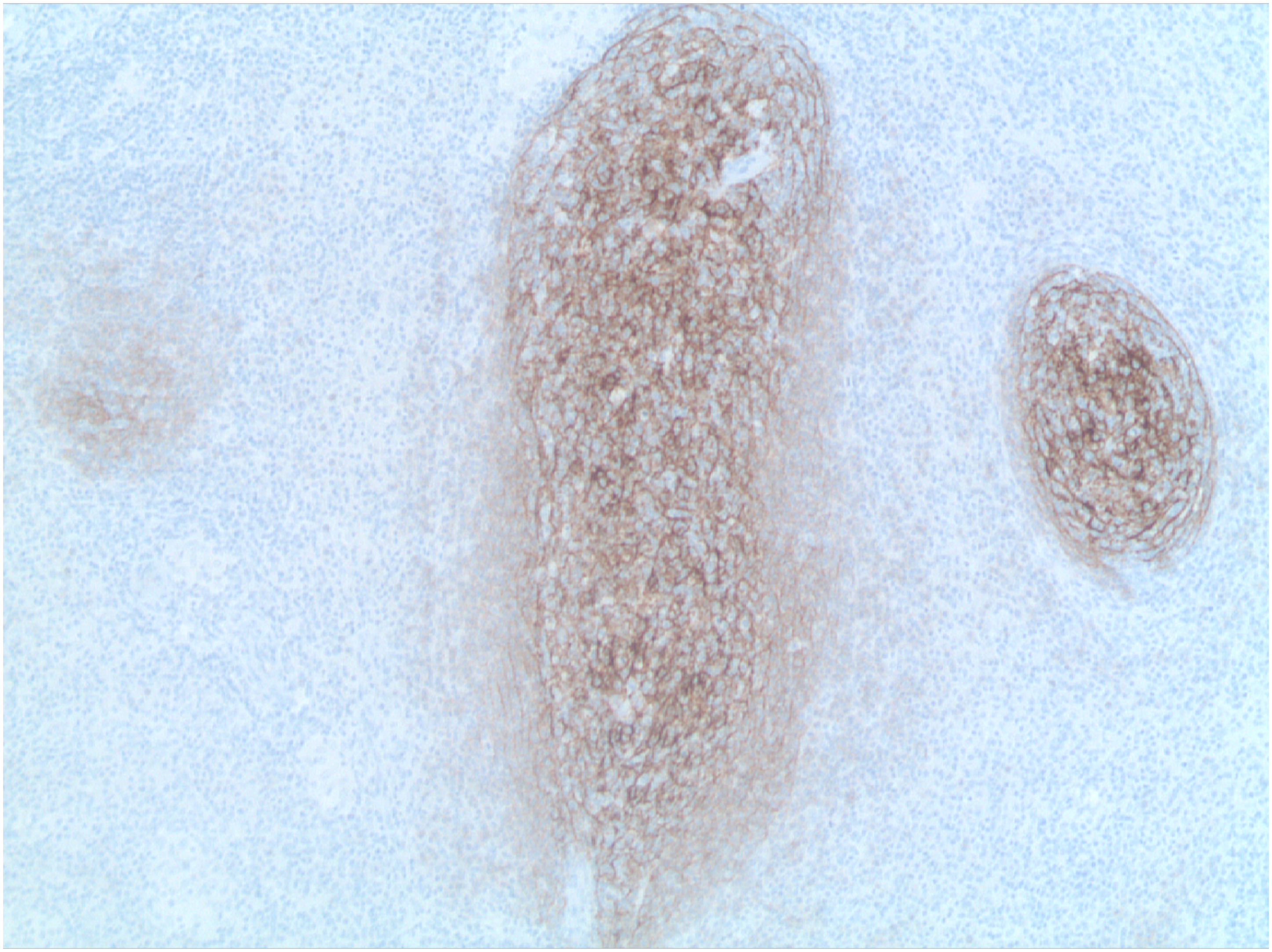

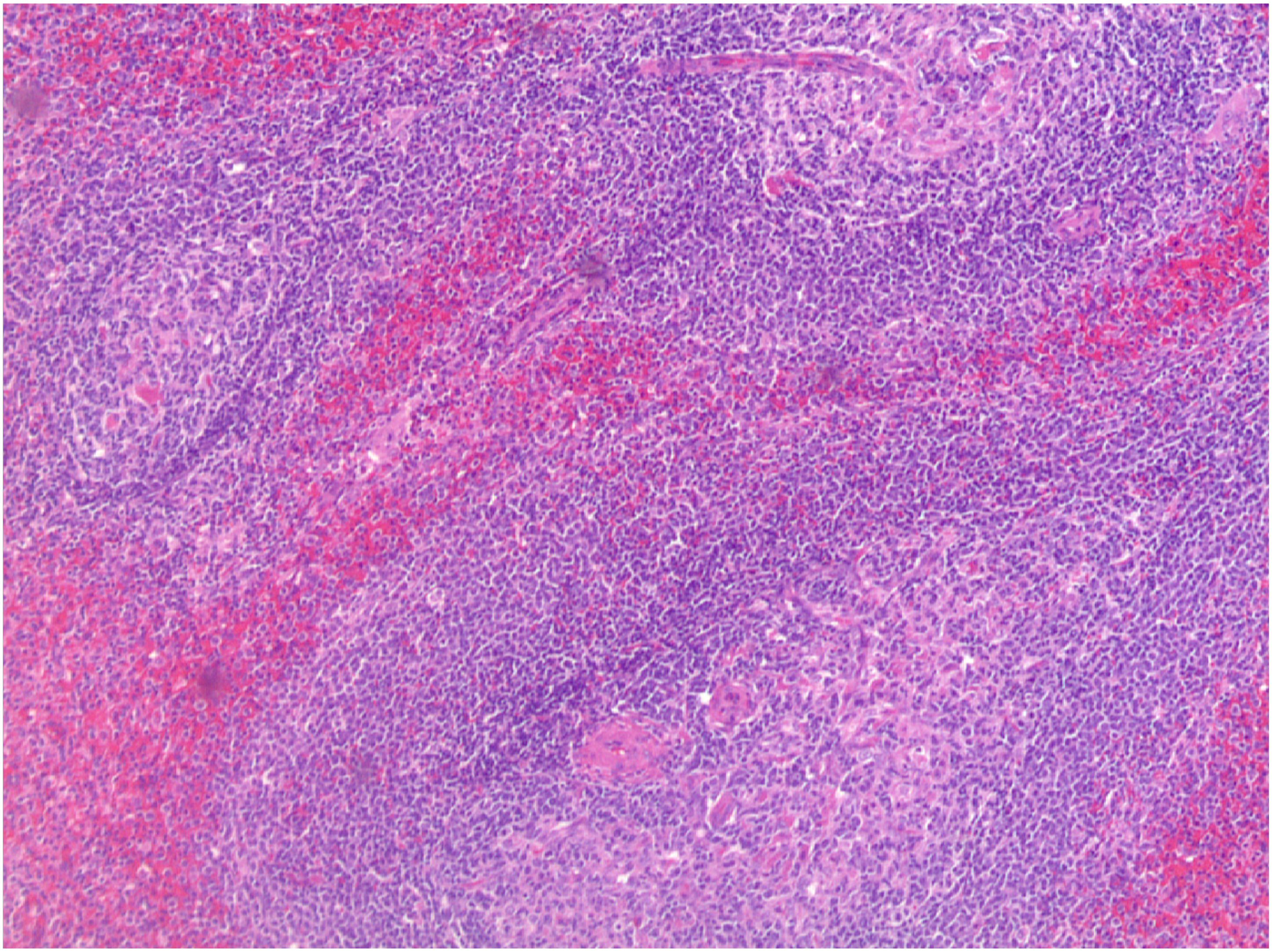

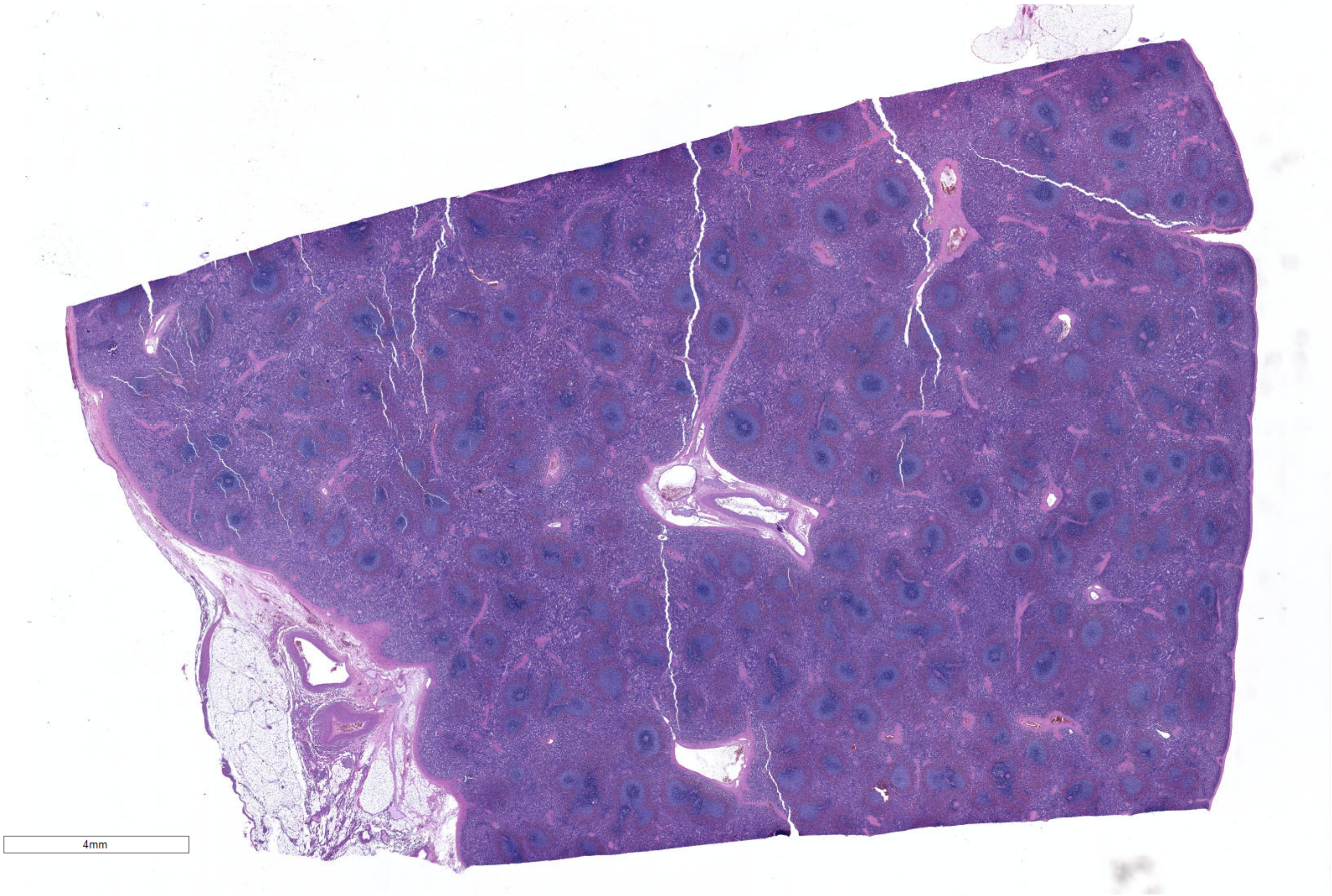
4mm

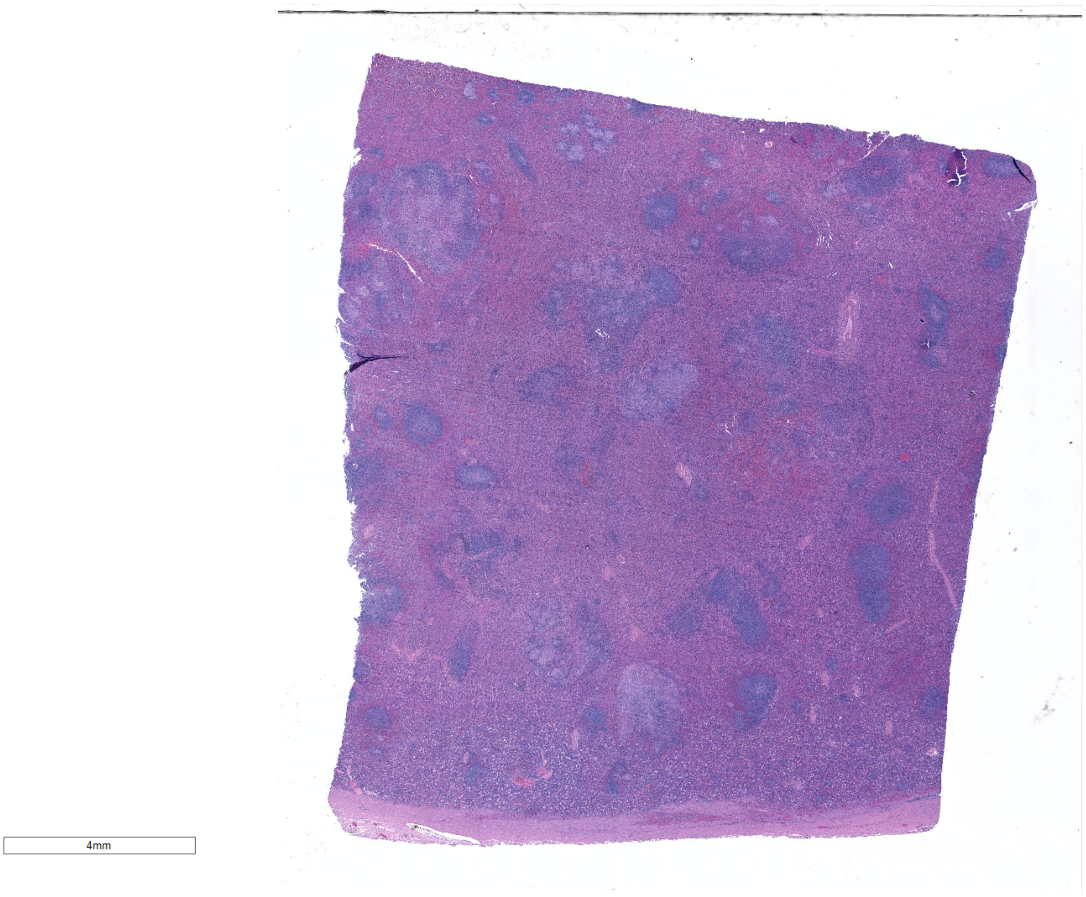
4mm

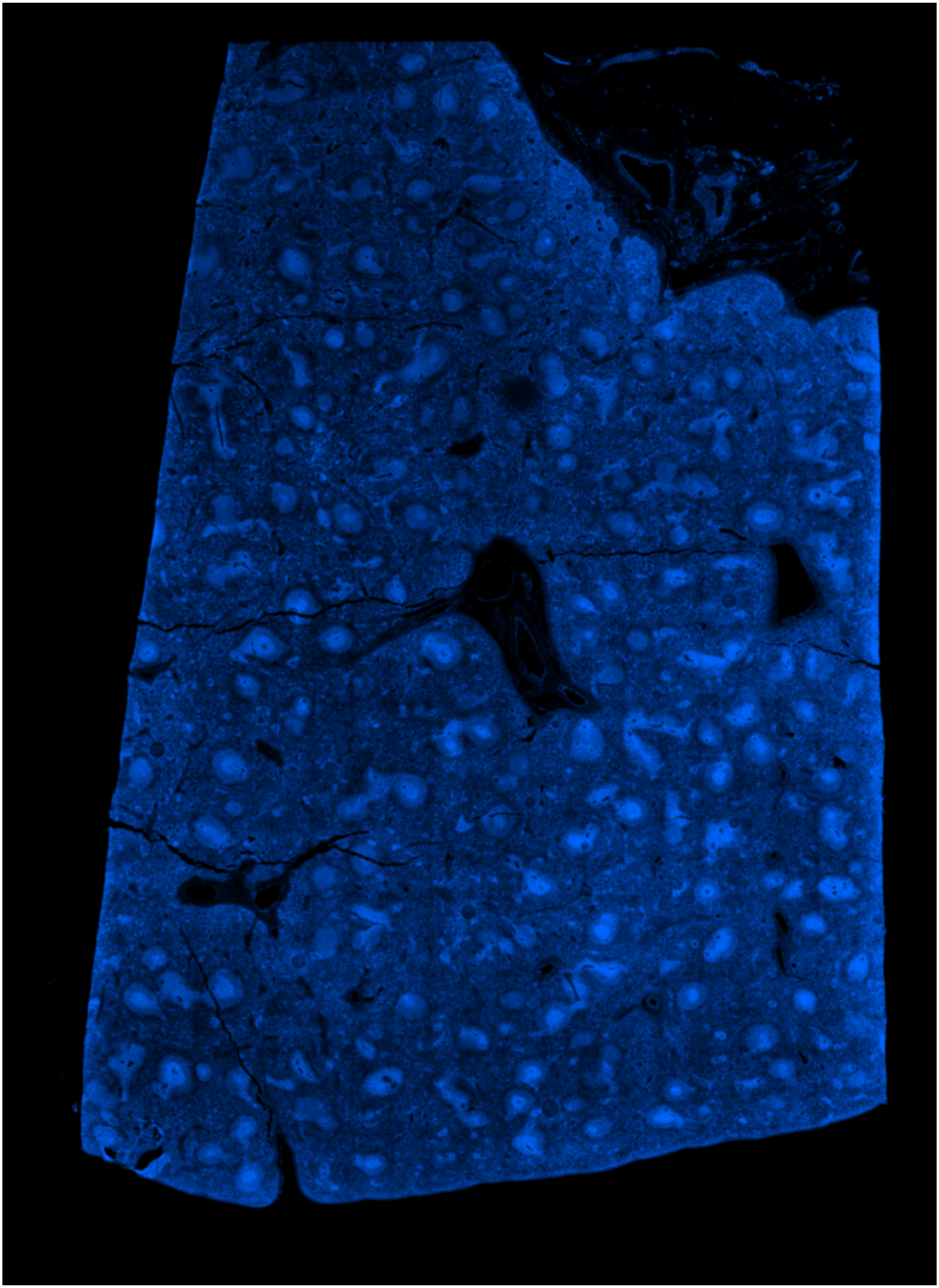

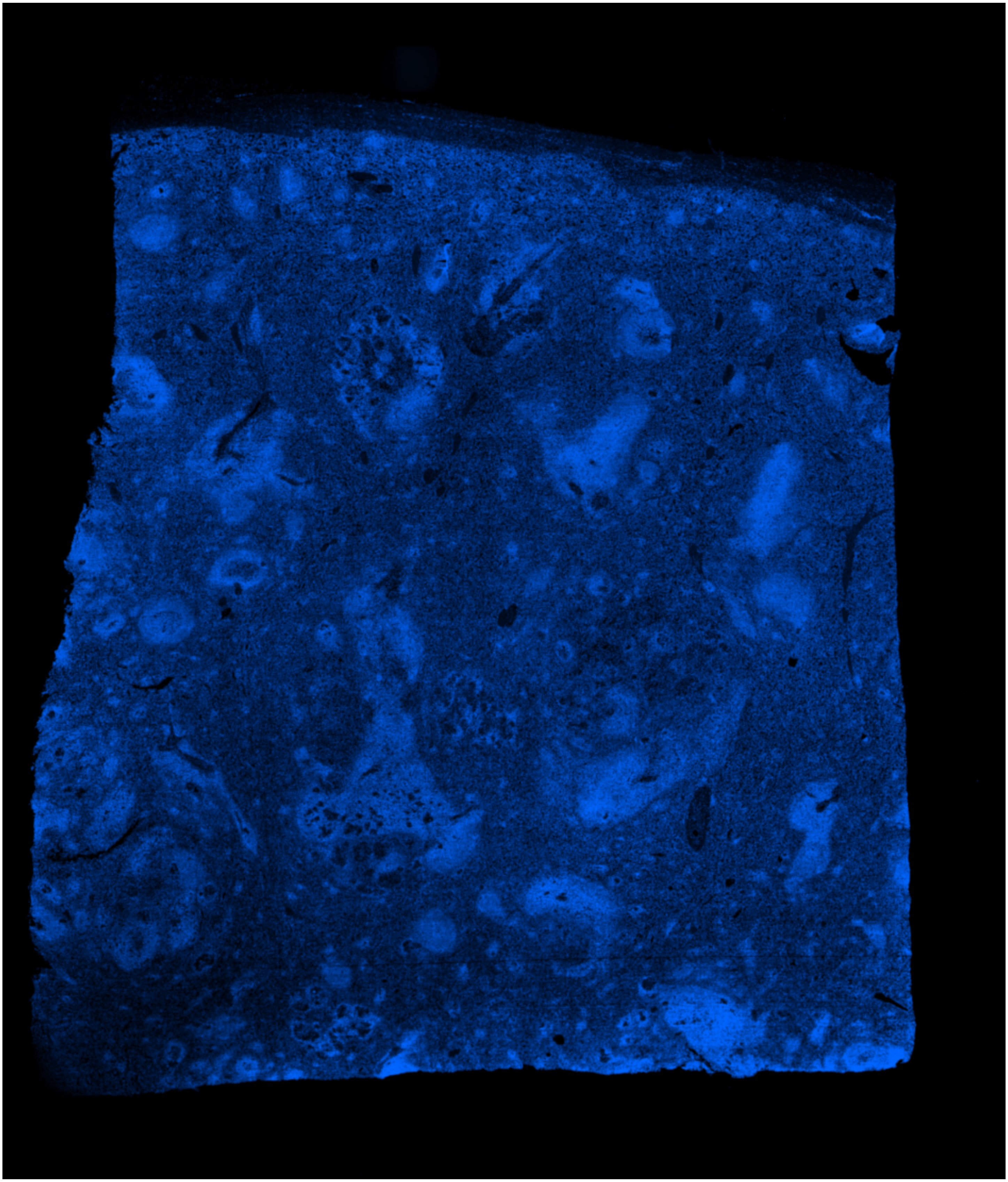

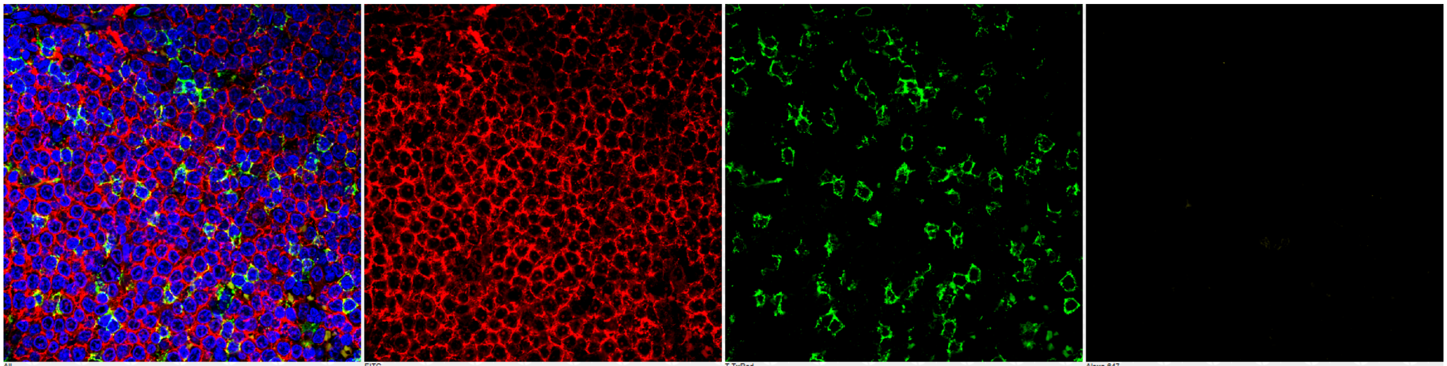

All FITC T-TxRed Alexa 647

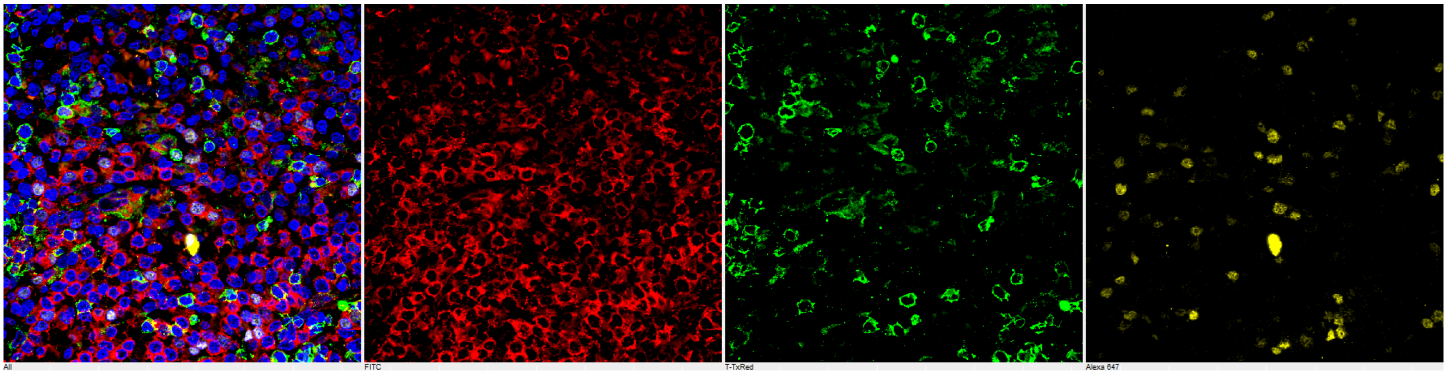
All
FITC
T-TxRed
Alexa 647

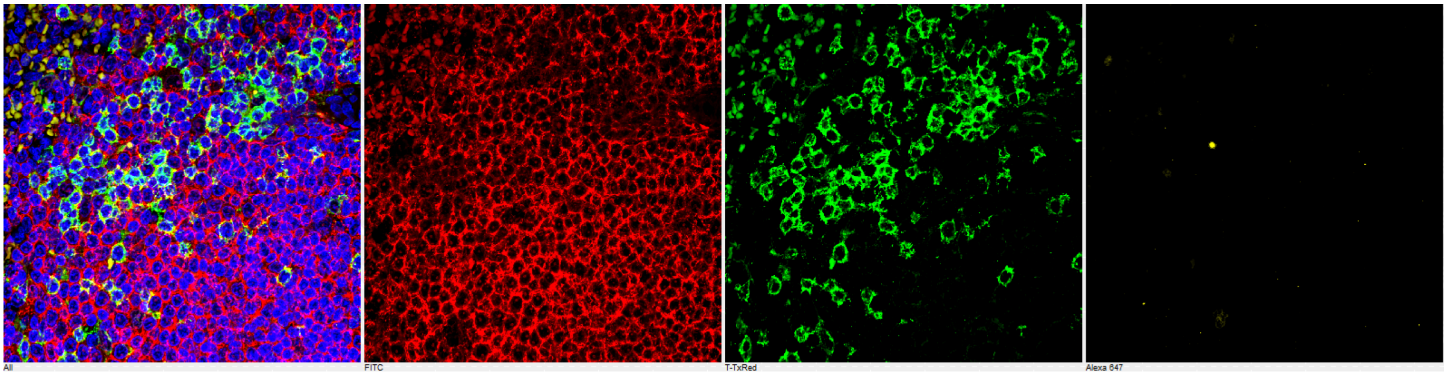
All
FITC
T-TxRed
Alexa 647

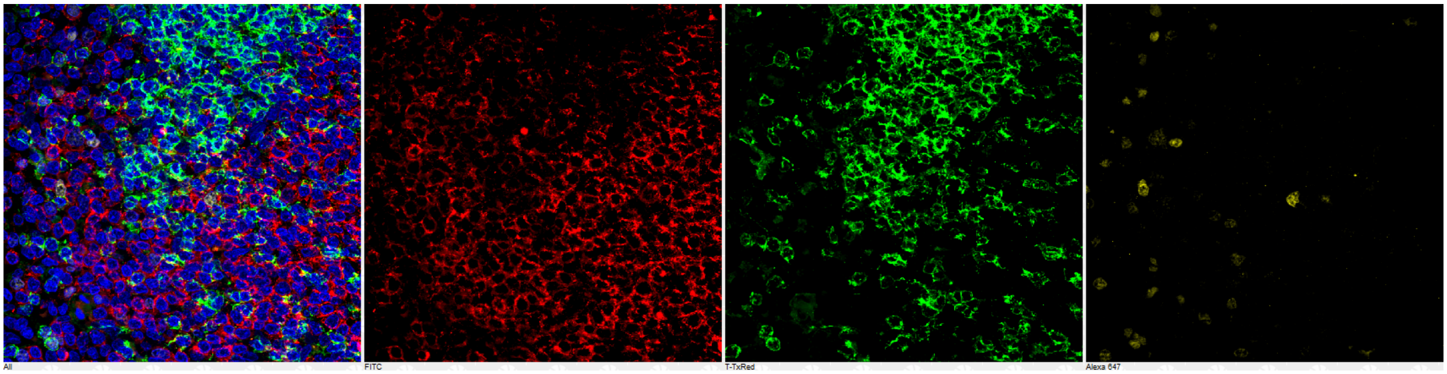
All
FITC
T-TxRed
Alexa 647

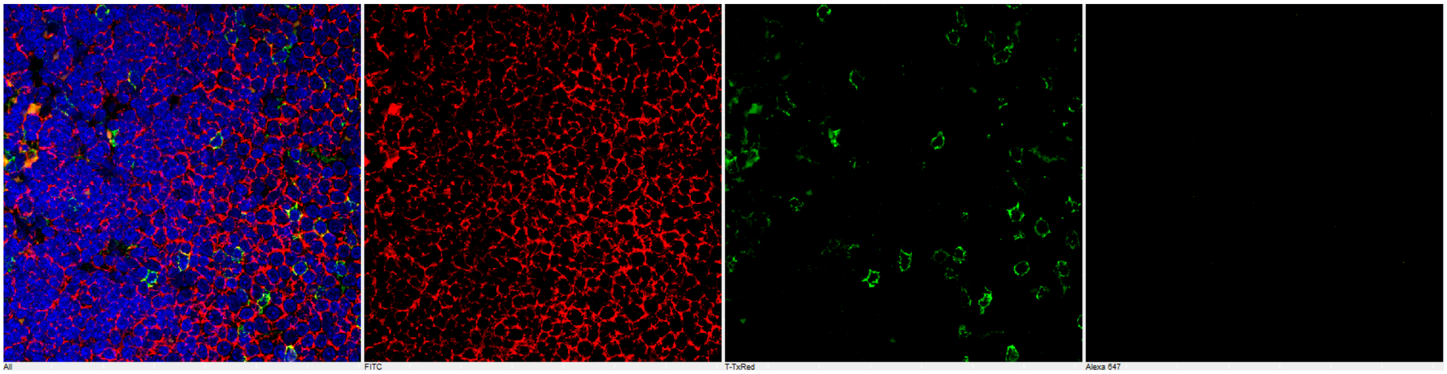
All
FITC
T-TxRed
Alexa 647

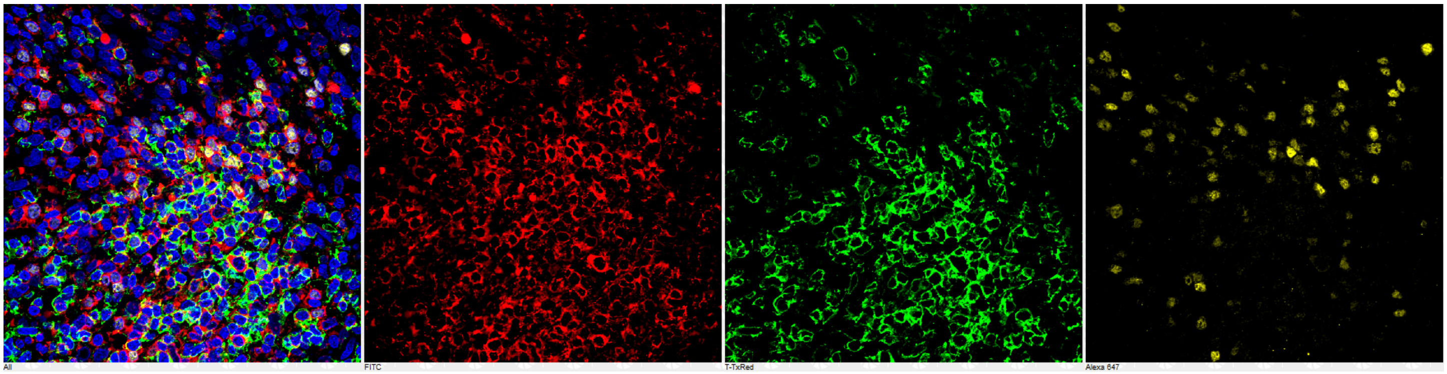

All
FITC
T-TxRed
Alexa 647

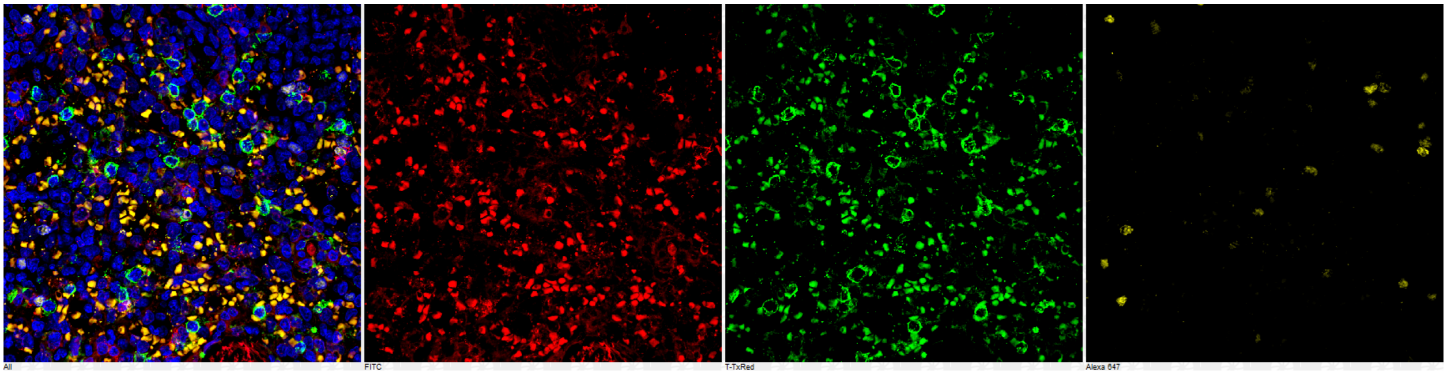
All
FITC
T-TxRed
Alexa 647

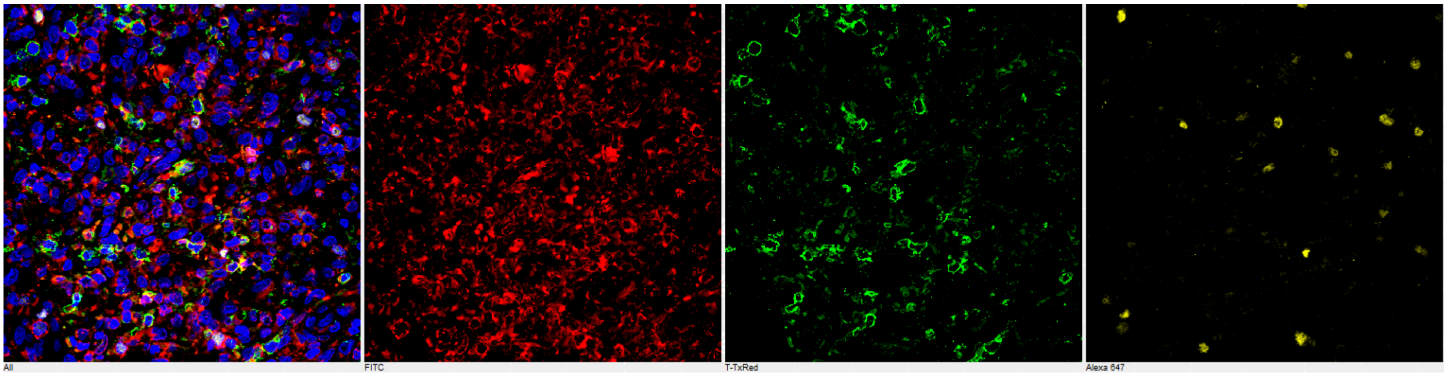
All
FITC
T-TxRed
Alexa 647

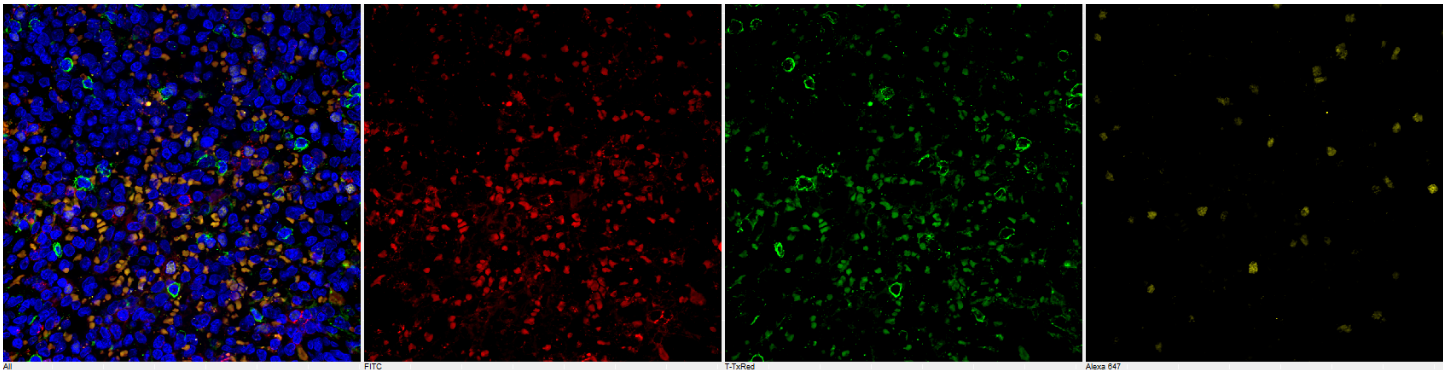

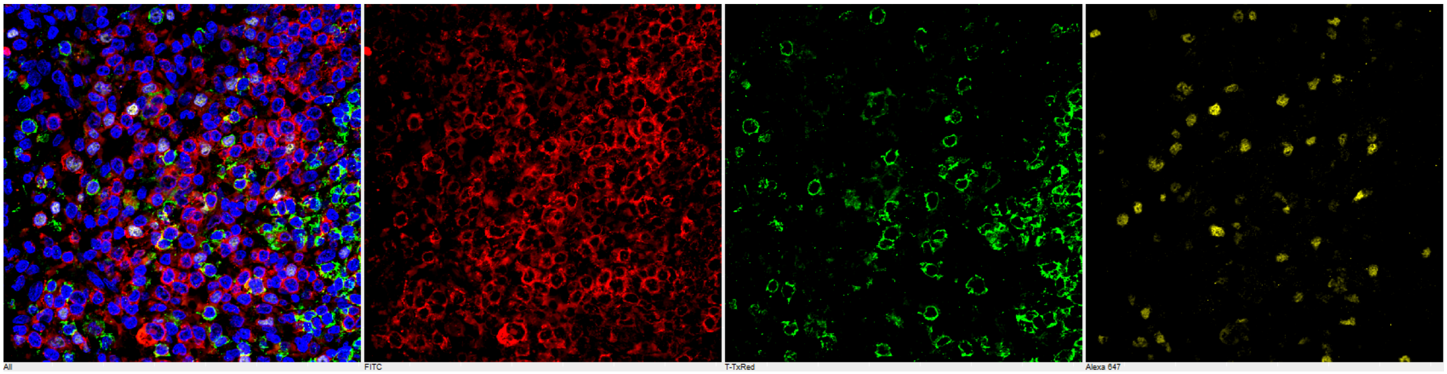
All
FITC
T-TxRed
Alexa 647

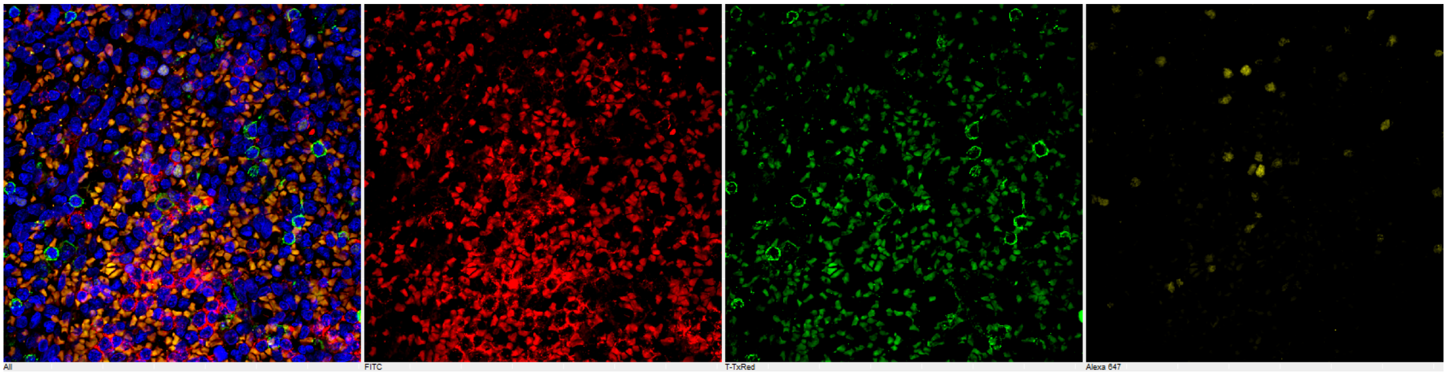
All
FITC
T-TxRed
Alexa 647

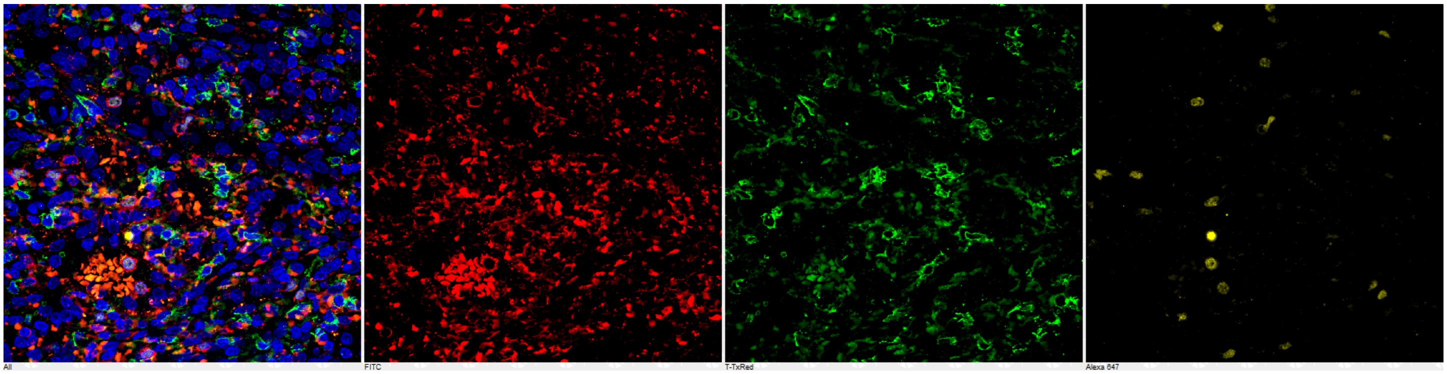

All
FITC
T-TxRed
Alexa 647
